# The roles of IRF8 in nonspecific orbital inflammation: an integrated analysis by bioinformatics and machine learning

**DOI:** 10.1186/s12348-024-00410-4

**Published:** 2024-06-20

**Authors:** Zixuan Wu, Jinfeng Xu, Yi Hu, Xin Peng, Zheyuan Zhang, Xiaolei Yao, Qinghua Peng

**Affiliations:** 1 Hunan University of Traditional Chinese Medicine, Changsha, 410208 Hunan Province China; 2 Department of Ophthalmology, the First Affiliated Hospital of Hunan University of Traditional Chinese Medicine, Changsha, 410007 Hunan Province China; 3 Dongying People’s Hospital (Dongying Hospital of Shandong Provincial Hospital Group), Dongying, Shandong 257091 PR China; 4 Ophthalmology Department, The First Hospital of Hunan University of Chinese Medicine, Changsha, 410011 China

**Keywords:** Nonspecific orbital inflammation (NSOI), IRF8, Lasso regression, SVM-RFE, Autoimmune inflammatory disorder

## Abstract

**Background:**

Nonspecific Orbital Inflammation (NSOI) represents a persistent and idiopathic proliferative inflammatory disorder, characterized by polymorphous lymphoid infiltration within the orbit. The transcription factor Interferon Regulatory Factor 8 (IRF8), integral to the IRF protein family, was initially identified as a pivotal element for the commitment and differentiation of myeloid cell lineage. Serving as a central regulator of innate immune receptor signaling, IRF8 orchestrates a myriad of functions in hematopoietic cell development. However, the intricate mechanisms underlying IRF8 production remain to be elucidated, and its potential role as a biomarker for NSOI is yet to be resolved.

**Methods:**

IRF8 was extracted from the intersection analysis of common DEGs of GSE58331 and GSE105149 from the GEO and immune- related gene lists in the ImmPort database using The Lasso regression and SVM-RFE analysis. We performed GSEA and GSVA with gene sets coexpressed with IRF8, and observed that gene sets positively related to IRF8 were enriched in immune-related pathways. To further explore the correlation between IRF8 and immune-related biological process, the CIBERSORT algorithm and ESTIMATE method were employed to evaluate TME characteristics of each sample and confirmed that high IRF8 expression might give rise to high immune cell infiltration. Finally, the GSE58331 was utilized to confirm the levels of expression of IRF8.

**Results:**

Among the 314 differentially expressed genes (DEGs), some DEGs were found to be significantly different. With LASSO and SVM-RFE algorithms, we obtained 15 hub genes. For biological function analysis in IRF8, leukocyte mediated immunity, leukocyte cell-cell adhesion, negative regulation of immune system process were emphasized. B cells naive, Macrophages M0, Macrophages M1, T cells CD4 memory activated, T cells CD4 memory resting, T cells CD4 naive, and T cells gamma delta were shown to be positively associated with IRF8. While, Mast cells resting, Monocytes, NK cells activated, Plasma cells, T cells CD8, and T cells regulatory (Tregs) were shown to be negatively linked with IRF8. The diagnostic ability of the IRF8 in differentiating NSOI exhibited a good value.

**Conclusions:**

This study discovered IRF8 that are linked to NSOI. IRF8 shed light on potential new biomarkers for NSOI and tracking its progression.

**Supplementary Information:**

The online version contains supplementary material available at 10.1186/s12348-024-00410-4.

## Introduction

Non-Specific Orbital Inflammation (NSOI) is characterized as a benign, non-infectious inflammatory disorder localized within the orbital region, absent of discernible systemic or localized etiological factors. It accounts for a substantial 6-16% of all ocular abnormalities and 11% of neoplastic conditions within the orbital domain. Predominantly affecting middle-aged individuals, NSOI exhibits a marked female preponderance [1, 2]. Despite its significant prevalence, the intricate pathophysiological mechanisms orchestrating NSOI largely remain shrouded in mystery. Several studies in the literature propose potential associations with a spectrum of conditions including Streptococcal pharyngitis, viral upper respiratory infections, and a diverse array of autoimmune disorders, such as rheumatologic diseases, multifocal fibrosis, and Crohn’s disease [2, 3]. The clinical manifestations of NSOI are notably heterogeneous, featuring a variety of presentations including dacryoadenitis, dacryoadenitis, and myositis impacting one or multiple extraocular muscles, among other unconventional presentations [4]. While systemic corticosteroids are universally acknowledged as the therapeutic cornerstone for presumptive diagnoses, their protracted use is frequently associated with a myriad of well-documented adverse effects [5]. Concerningly, even with successful corticosteroid intervention, the recurrence rates loom over 50% [6]. NSOI remains elusive, primarily hypothesized to be linked to immune or autoimmune mechanisms, infections, or drug reactions. Despite its classification as non-infectious, there are suggestions that subclinical infections or immune responses secondary to such infections could trigger NSOI [7]. Notably, instances following upper respiratory tract infections, influenza vaccinations, and paranasal sinusitis support the hypothesis that preceding infections may induce NSOI. Furthermore, studies implicating Epstein-Barr virus (EBV), a herpesvirus known to infect lymphocytes and epithelial cells and associated with various cancers, suggest it as a potential etiological factor in NSOI [8]. Indeed, research by Jin et al. identified EBV DNA in 16 NSOI tissue samples, with a detection rate of 94% [9].

NSOI is also frequently associated with rheumatologic conditions, suggesting an immune-mediated origin. Mombaerts highlighted that orbital myositis is linked with autoimmune diseases in approximately 10% of cases, and a study in the Netherlands indicated a tentative association between NSOI and autoimmune diseases [10]. This points to a possible genetic predisposition or a dysregulated immune response in patients with concurrent NSOI and autoimmune conditions, where the orbital inflammation is considered a secondary immune response rather than part of a multifocal disorder. NSOI represents a diverse group of disorders and is primarily diagnosed by exclusion [11]. Its classic clinical presentation includes acute or subacute onset of symptoms such as proptosis, periorbital swelling and erythema, pain, diplopia, and visual disturbances, typically responsive to oral corticosteroids. Corticosteroids are the foundational treatment for NSOI, often leading to rapid symptom resolution. Systemic administration of prednisolone usually brings about marked improvement within 48 hours, while local corticosteroid injections offer an effective alternative for targeted relief [12]. When corticosteroids fail, low-dose radiotherapy, typically between 20 to 30 Gy delivered in 2 Gy fractions, is implemented. In cases of resistance or recurrence post-corticosteroid therapy, immunosuppressive agents such as methotrexate, cyclosporine A, mycophenolate mofetil, and cyclophosphamide are utilized. For particularly recalcitrant NSOI, biological agents including infliximab, adalimumab, etanercept, daclizumab, abatacept, tocilizumab, and rituximab are reserved [13]. Despite their efficacy, corticosteroids have a limited long-term cure rate of 37% and a recurrence rate of 52%, with prolonged use associated with systemic side effects like insomnia, hyperglycemia, weight gain, and cataracts. The recent discovery of IRF8's role in NSOI offers new diagnostic and prognostic possibilities [14]. Long-term systemic steroid use, recommended for at least three months to minimize recurrence, underscores the need for more targeted treatments with fewer side effects. In some cases, strabismus surgery or orbital decompression may be necessary to correct residual diplopia due to rectus muscle fibrosis or persistent proptosis [15]. A more prompt and targeted therapeutic approach, similar to those developed for thyroid eye disease (e.g., tocilizumab, teprotumumab, etanercept), might mitigate extraocular muscle fibrosis. Diagnosing NSOI remains challenging due to its unclear etiology and the absence of specific laboratory tests or consensus diagnostic criteria. It heavily relies on the clinician's experience and insight, and the conditions excluded in its diagnosis can vary widely among practitioners [16]. As such, unraveling the molecular pathways intrinsic to NSOI is of paramount importance for the development of innovative therapeutic approaches, aimed at reducing recurrence and enhancing patient prognosis.

Interferon Regulatory Factor 8 (IRF8), also recognized as IFN Consensus Sequence Binding Protein (ICSBP), is a cardinal transcription factor predominantly expressed in hematopoietic cells [17]. It has been meticulously studied across various stages of the hematopoietic system, including multi-, oligo-, and committed progenitors, alongside immature and mature blood and immune cells [18]. IRF8 plays a pivotal role in lineage-committed progenitors, where it selectively curtails neutrophil production whilst augmenting monocyte generation [19]. Consequently, IRF8-null mice exhibit elevated neutrophil counts and diminished monocyte populations in both bone marrow and peripheral blood [20]. Structurally, IRF8 encompasses a DNA Binding Domain at its N-terminus and a middle IRF Association Domain (IAD). Typically, IRF8 forms heterodimers with partner transcription factors via its IAD, binding to specific DNA sequences, thus acting either as an activator or a repressor to facilitate the transcription of downstream genes [21]. In certain instances, IRF8 has been reported to directly regulate the transcription of several genes. IRF8 serves as a significant regulator in the development of macrophages, dendritic cells, and B-cells and is implicated in the differentiation of Th17, Th9, and Treg cells [22]. Furthermore, it has been documented to modulate the adaptive natural killer cell response and inflammasome activation [23]. Prior research has evidenced that IRF8 amplifies the second phase of type I IFN induction in dendritic cells by sustaining RNA polymerase II recruitment to the IFN promoters in response to infections by murine cytomegalovirus and Newcastle disease viruses [24]. Thus, a nuanced understanding of the roles and regulatory networks involving IRF8 and other chemokines is instrumental for devising targeted therapeutic interventions in retinal inflammatory diseases and beyond.

**Fig. 1:**
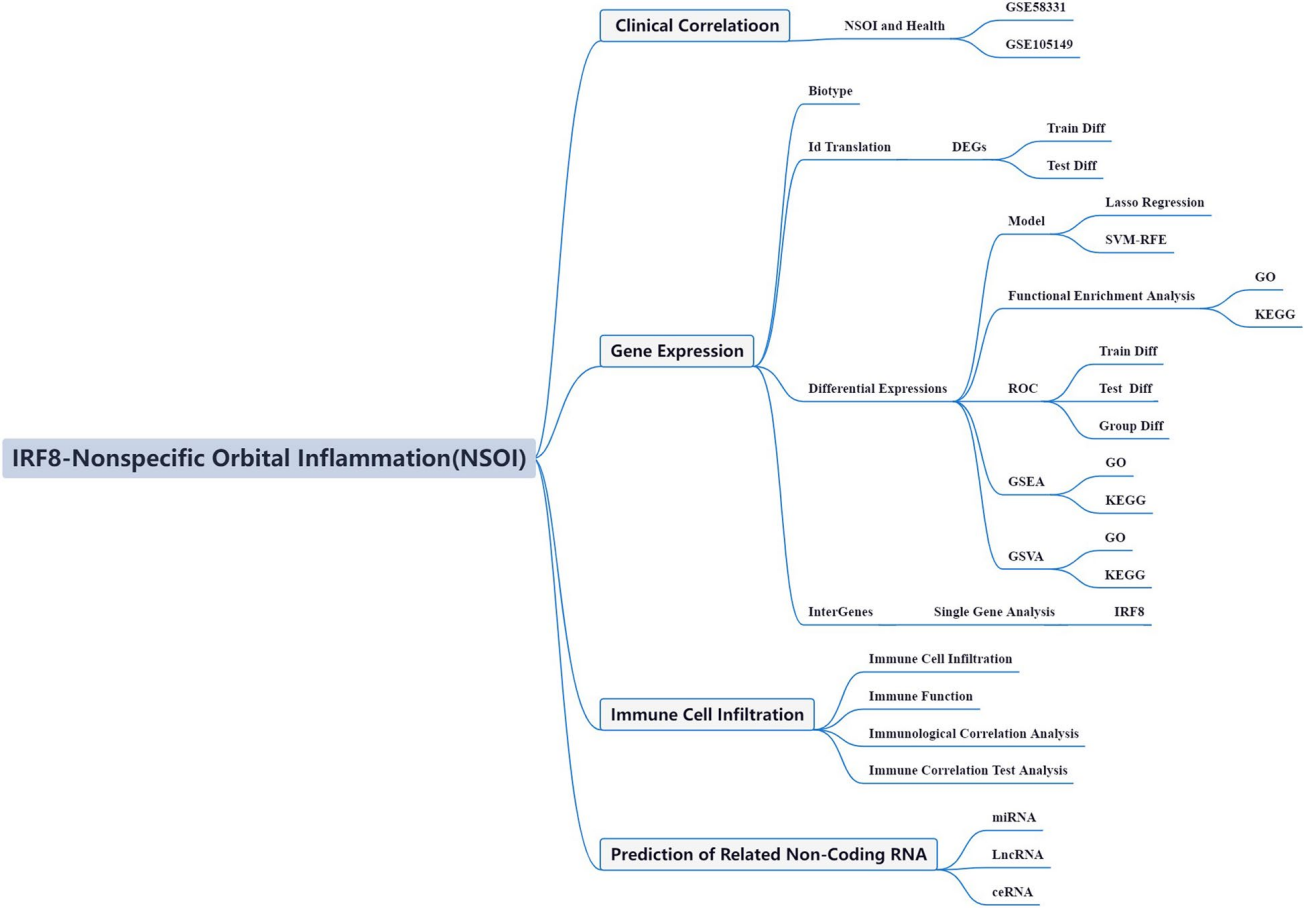
Framework

Recent advancements in oncology have identified a distinct metabolic phenotype in neoplastic cells that reconfigures the immune microenvironment. This environment is a complex network filled with various cellular entities, characterized by poor nutrient and oxygen availability due to an inefficient vascular architecture [25]. Within this evolving scientific framework, there is an increased emphasis on the role of non-tumor immune cells. The pioneering work by Sharma et al. suggests that the immunosuppressive structure of the immune microenvironmen, populated by diverse immune cells, may be crucial in mediating mechanisms that confer resistance to immunotherapy. Empirical studies indicate that many cancers utilize complex immunosuppressive tactics; for instance, regulatory T cells release a variety of immunomodulatory cytokines, while myeloid and stromal cells activate inhibitory checkpoints like PD-1, CTLA4, and TIM-3 [26, 27]. Despite these insights, the interplay between these immunological dynamics and NSOI remains a challenging enigma, urging a deeper investigation into the immune microenvironment's manipulation in cancer progression and response to immunotherapy [28]. This exploration could reveal novel therapeutic avenues specifically designed for NSOI patients. While targeting both IRF8 and immunotherapy could be promising for NSOI treatment, a detailed understanding of the interaction between IRF8, immunogenicity, and immunotherapeutic interventions is still lacking. Utilizing high-throughput data analysis and advanced bioinformatics, we have begun to untangle the complex gene networks across various diseases, shedding light on potential molecular mechanisms [29, 30]. The extensive transcriptome sequencing data and clinical annotations from the NSOI Initiative allow for a detailed examination of altered transcriptional landscapes and the molecular pathways involved in NSOI. However, there is a notable gap as no study has yet used bioinformatics to specifically explore the role of NSOI within the disease context. Thus, our research aims to investigate IRF8-related GEO through the analytical lens of NSOI, as illustrated in Fig. 1.

## Materials and methods

We used the approaches proposed by Zi-Xuan Wu, et al. 2023 [31].

### Source of transcriptional profiling data

GEO was searched for mRNA expression. Series: GSE58331 and GSE105149. Platform: GPL570-55999. GSE58331 and GSE105149 were used as the trian and test groups respectively. Strategy for searching ('eye' [MeSH] mRNA [All Fields] and normal) AND ('Homo sapiens' [Organism] AND 'Non-coding RNA profiling by array' [Filter]). Specifically, this investigation harnessed the datasets GSE58331 and GSE105149, underpinned by the GPL570-55999 platform. GSE58331 functioned as the training cohort, while GSE105149 constituted the testing group. The essential characteristics of the datasets are summarized in Table 1.

**Table 1:** The clinical characteristics of patients

Categories	NOSI	
	GSE58331	GSE105149
Variables	Number of samples	Number of samples
Gender		
Male/Female	19/56	18/9
Diagnosis		
Diseases/Normal	75/29	27/29
Tissue		
Anterior Orbit/Lacrimal gland	42/33	0/27

### Transcriptomic data refinement and preprocessing

The acquired probe-centric expression matrices were transmuted into gene-level expression matrices, capitalizing on the auxiliary probe annotation documentation. In instances where multiple probes corresponded to a solitary gene, an arithmetic mean of these probe values was computed to typify the definitive expression metric of the respective gene. Subsequent to the standardization of the datasets, batch effect normalization was executed employing the SVA package. The efficacy of batch effect rectification was gauged through PCA. Differential expression analyses between NSOI and control groups were conducted utilizing the Linear Models for Microarray Data (limma) package. Criteria for defining differentially expressed genes (DEGs) were set at an absolute log fold change (|log FC|) greater than 1 and an adjusted *p*-value less than 0.05, with the aim of isolating immune infiltration-associated genes in NSOI cases.

### Immune landscape characterization

To elucidate the interface between IRF8 and immune-related biological undertakings, both CIBERSORT and ESTIMATE algorithms were enlisted to scrutinize the TME attributes within individual samples. Differential representation of 22 immunocyte subsets between NSOI and control specimens was statistically validated employing the Wilcoxon rank-sum test.

### Predictive modeling and computational learning

The model framework was scaffolded through Lasso regression analysis coupled with cross-validation, deploying the glmnet package. Subsequent validation of the model's fidelity and precision was performed through the SVM-RFE algorithm, leveraging the e1071 package. Cross-validation procedures were enacted to discern the model's margin of error and acumen. The consequential ranking of feature genes was synthesized from both the Lasso and SVM machine learning paradigms. Genes deciphered from these integrated approaches were then poised for subsequent analytical endeavors. The ROC curve furnished an evaluative framework for the diagnostic potency of identified biomarkers.

### Functional annotation via GO and KEGG pathway analyses

To delineate the biological functions and signaling pathways implicated in the differential expression landscape, GO and KEGG analyses were performed. The R statistical environment was employed to explore how variations in IRF8 expression modulate BP, MF, and CC.

### Integrated enrichment analysis using GSEA and GSVA

Global gene-set enrichment analyses, encompassing GSEA and GSVA, were utilized to identify functionally coherent gene sets and signaling cascades differentially active across the studied samples. Enrichment scores and accompanying visual representations were generated to discern dynamic activities and pathways across various risk stratifications. R was deployed to investigate the influence of differential IRF8 expression on BP, MF, CC, and implicated pathways.

### Biomarker-immune infiltrate correlation analyses

Spearman's rank correlation was invoked to gauge the relationship between diagnostic biomarkers and immune cell infiltration in the tissue microenvironment.

### Dissecting miRNA and lncRNA cross-talk in NSOI

Non-coding RNAs, notably miRNAs and lncRNAs, serve as pivotal modulators of gene expression. While miRNAs principally function through post-transcriptional regulation either by promoting or inhibiting mRNA degradation and translation, lncRNAs engage in multifaceted regulatory capacities, including chromatin remodeling, transcriptional activation, and interference mechanisms. Recent discoveries underscore the intricate interplay between miRNAs and lncRNAs, revealing ceRNA networks. Accordingly, this study aims to unearth common regulatory axes and developmental trajectories involving miRNAs and lncRNAs within the NSOI context.

### Construction of integrated mRNA-miRNA-lncRNA regulatory networks

Empirically validated target gene information for the common miRNAs and lncRNAs was retrieved from miRTarbase and PrognoScan databases. An intersecting regulatory network, encapsulating mRNA-miRNA-lncRNA interplay and their shared targets in NSOI, was assembled and visualized using Cytoscape software.

### Statistical considerations

Statistical assessment of gene expression disparities between the distinct cohorts was executed via the ggpubr package in R (version 4.3.1). For data adhering to a normal distribution, two-sample independent t-tests were utilized; alternatively, the Wilcoxon rank-sum test was applied for non-normally distributed data. A p-value threshold of less than 0.05 was deemed statistically significant for all tests.

## Results

### DEG identification and principal component analysis

**Fig. 2:**
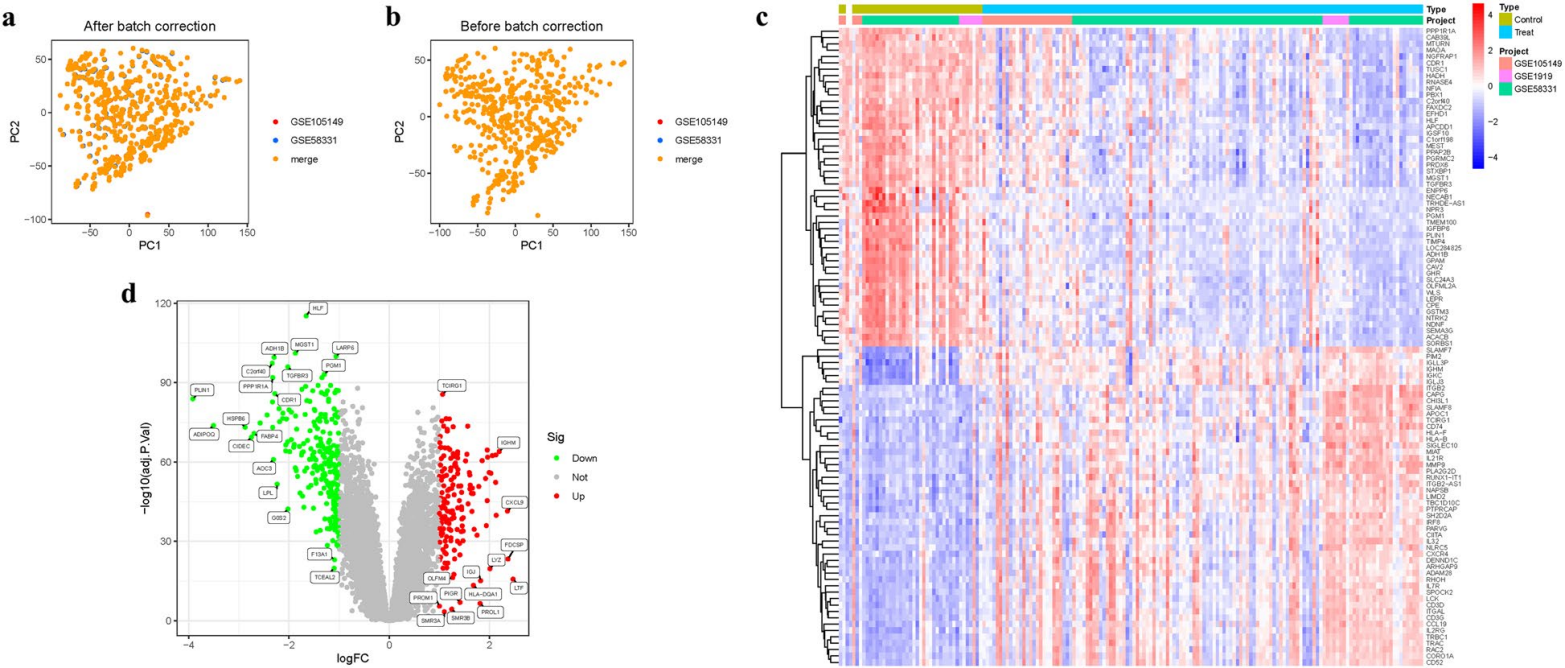
Principal Component Analysis. **a**-**b** Analysis of PCA. **c** Heatmap. **d** Volcano map

We integrated GSE58331 and GSE105149 and conducted batch match evidence integration. PCA corroborated the successful demarcation of patients into risk-specific cohorts (Fig. 2a-b). Among the 314 DEGs, some DEGs were found to be significantly different. In addition, some genes cluster in the treat group and some in the control group. Treat: PPP1R1A, CAB39L, MTURN, MAOA2, NGFRAP1, CDR1, etc. Control: ITGB2, CAPG, CHI3L1, SLAMF8, APOC1, TCIRG1, etc. (Fig. 2c). Some of these DEGs were significantly up-regulated (TCIRG1, IGHM, CXCL9, PROM1, PIGR, HLA-DQA1, etc). However, some genes were significantly down-regulated (HLF, ADH1B, MGST1, LARP6, PGM1, C2orf40, TGFBR3, etc). (Fig. 2d) (Table.S1).

### Construction of the model

**Fig. 3:**
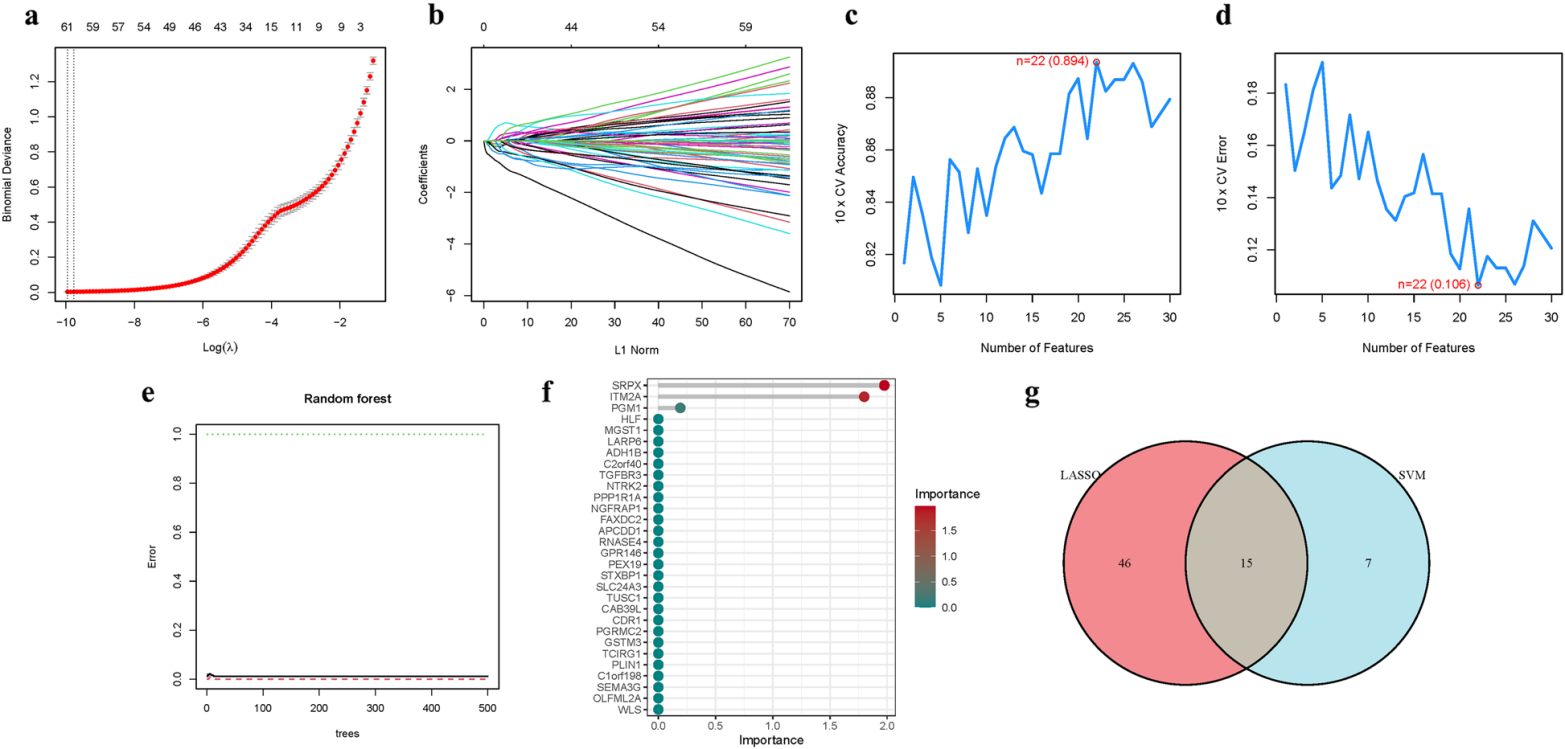
The development of the signature. **a** Regression of the NSOI-related genes using LASSO. **b** Cross-validation is used in the LASSO regression to fine-tune parameter selection. **c**-**d** Accuracy and error of this model. **e**-**f** Random forest analysis. **g** Venn

The LASSO, Cox regression analysis, and optimum value were used to establish a gene signature (Fig. 3a-b). The SVM-RFE was used to build the machine learning model to validate the model's accuracy and reliability. The accuracy of this model was 0.894, and the error was 0.106 (Fig. 3c-d). Some important genes were identified by Random forest analysis, and these genes included SRPX, ITM2A, PGM1, HLF, etc (Fig. 3e-f). We attempted to combine the key genes of these three algorithms to construct the model. However, it was found that only LASSO and SVM-RFE had the most stable key gene construction models. Finally, we obtained 15 hub genes (Fig. 3g) (Table.S3).

### DEG identification and visualization

**Fig. 4:**
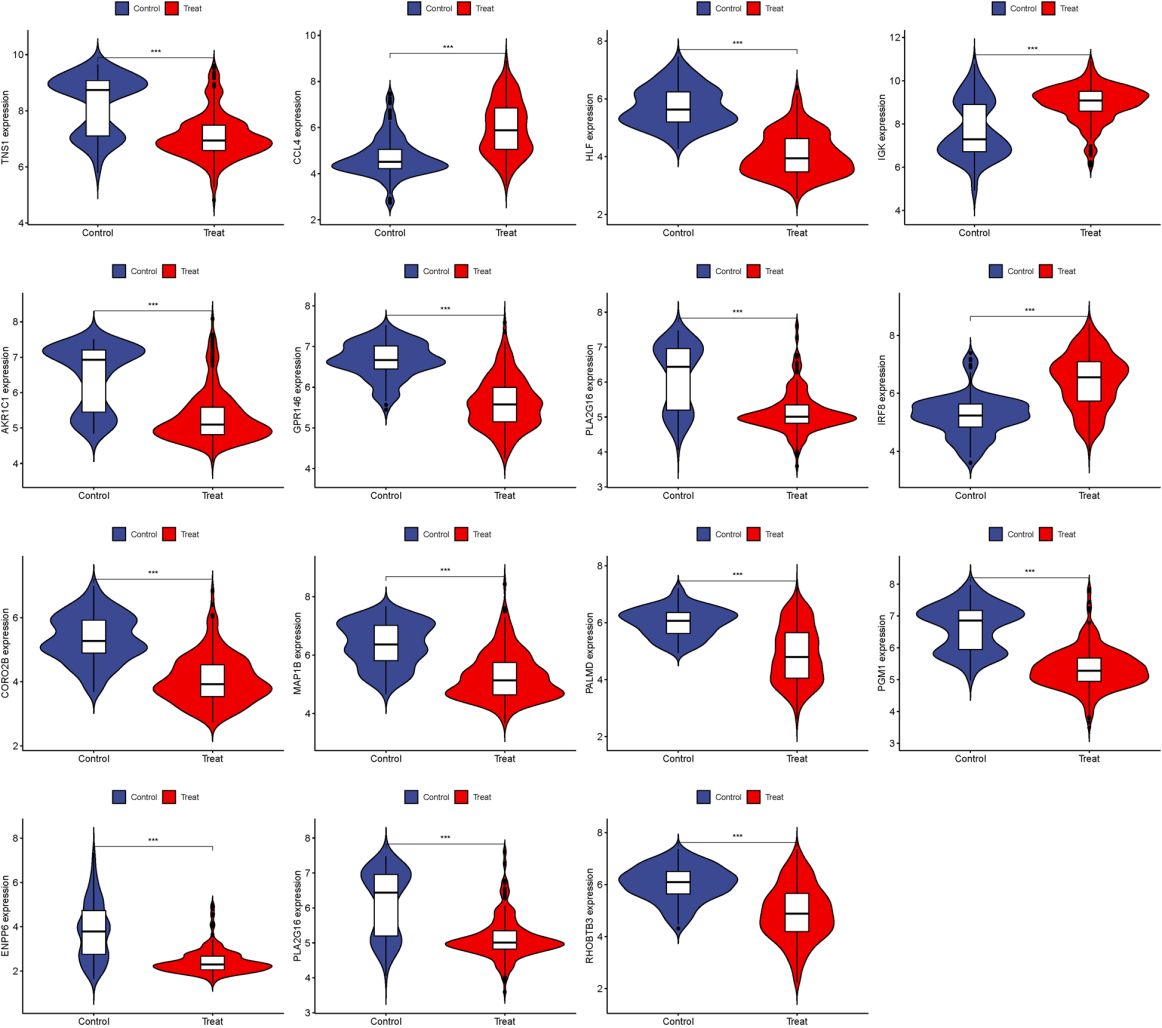
Expression of 15 hub genes in NSOI group and normal sample group respectively

**Fig. 5:**
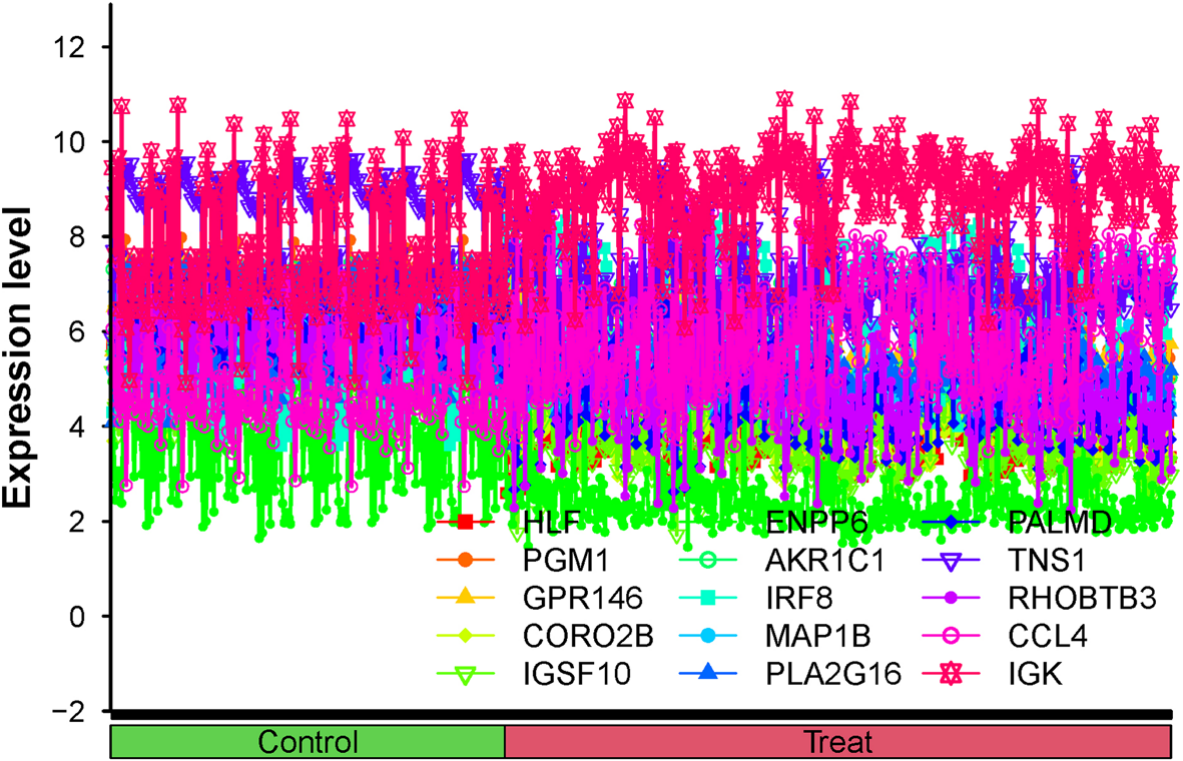
All hub genes are co-expressed in the same line plot

**Fig. 6:**
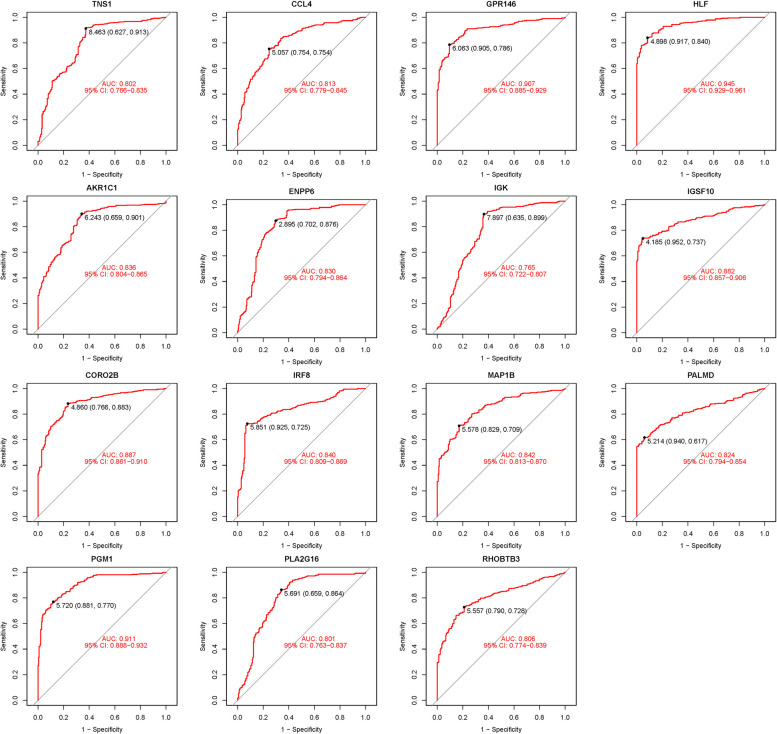
ROC of 15 hub genes

We visualized these 15 hub genes in the NSOI group and the normal sample group respectively (Fig. 4). In addition, we also put these genes in the whole graph for visual comparison (Fig. 5). In the confirmation of 15 hub genes, we analyzed the ROC of these genes, showing that the accuracy of these genes is high. HLF (AUC: 0.945), PGM1 (AUC: 0.911), GPR146 (AUC: 0.907), IRF8 (AUC: 0.840), TNS1 (AUC: 0.802), PLA2G16 (AUC: 0.801), PALMD (AUC: 0.824), CCL4 (AUC: 0.813), IGK (AUC: 0.765), CORO2B (AUC: 0.887), IGSF10 (AUC: 0.882), AKR1C1 (AUC: 0.836), ENPP6 (AUC: 0.830), MAP1B (AUC: 0.842), RHOBTB3 (AUC: 0.806) (Fig. 6).

### Validation of hub genes

**Fig. 7:**
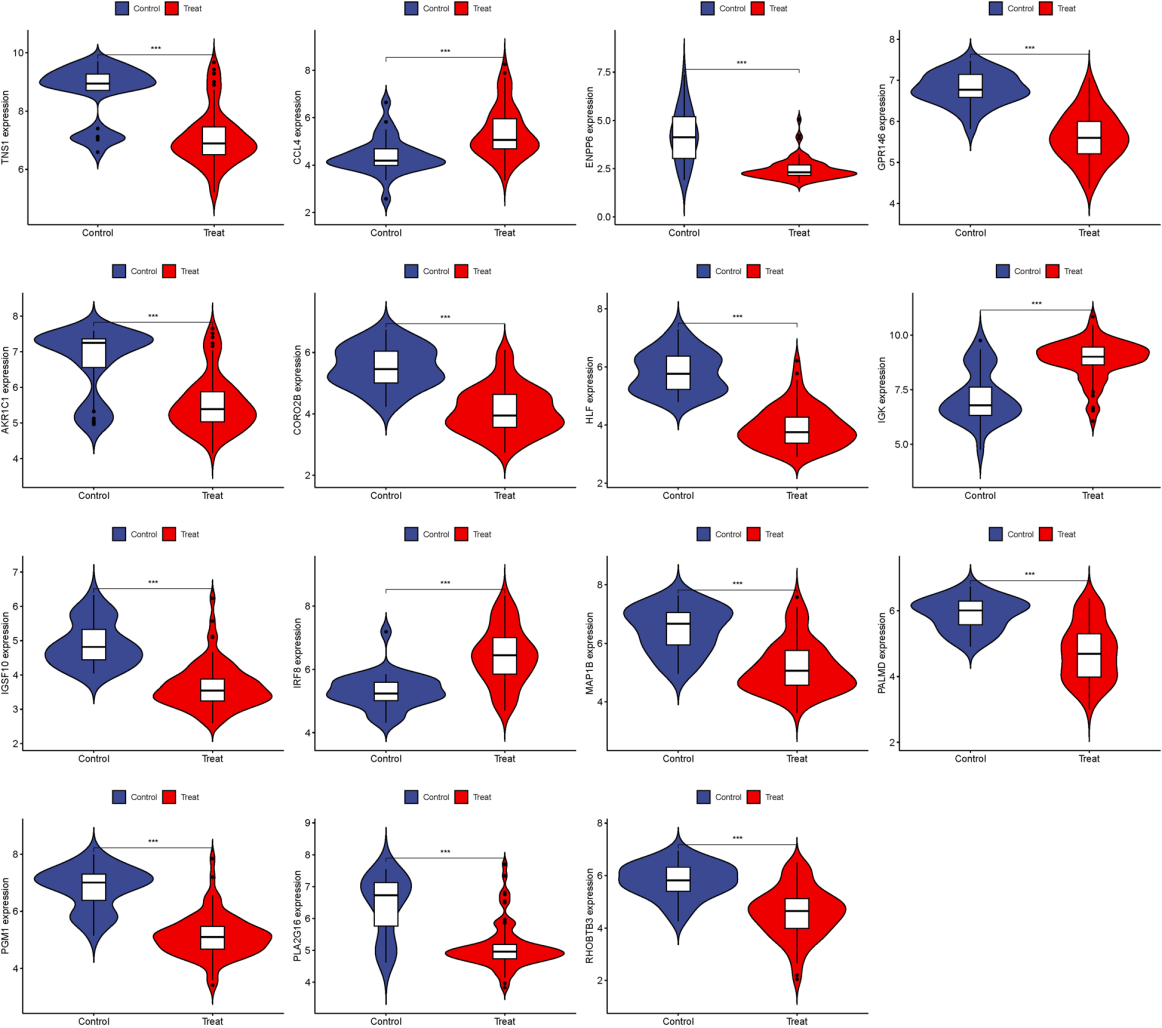
Expression of 15 hub genes in GSE58331 analysis

**Fig. 8:**
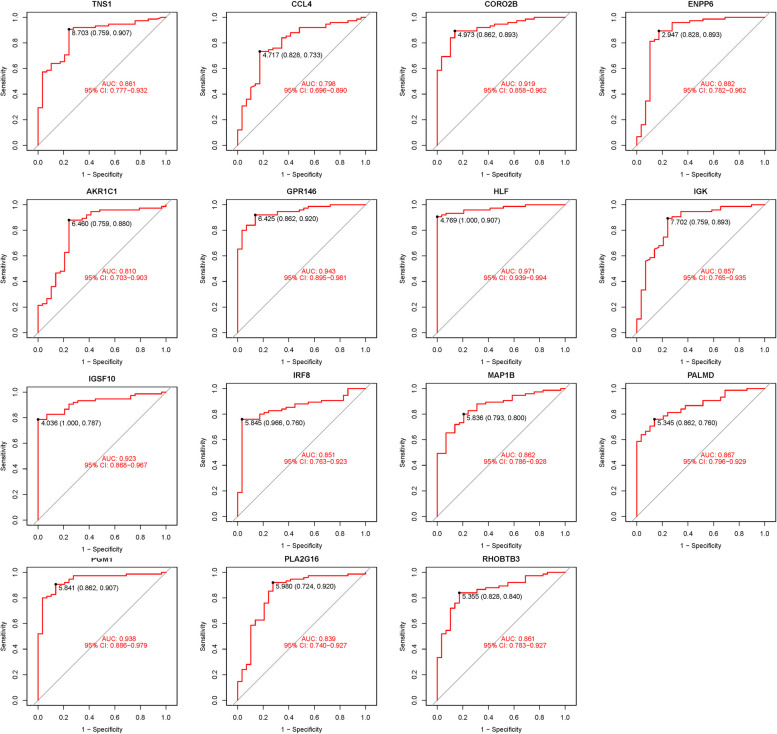
ROC of 15 hub genes

GSE58331 was used for validation to boost our model's confidence and prediction accuracy of these hub genes. What's interesting is that these DEGs are showed significant differences in GSE58331 analysis (Fig. 7). In the GSE58331 analysis of 15 hub genes, we analyzed the ROC of these genes, showing that the accuracy of these genes is high. HLF (AUC: 0.971), PGM1 (AUC: 0.938), GPR146 (AUC: 0.943), IRF8 (AUC: 0.851), TNS1 (AUC: 0.861), PLA2G16 (AUC: 0.839), PALMD (AUC: 0.867), CCL4 (AUC: 0.798), IGK (AUC: 0.857), CORO2B (AUC: 0.919), IGSF10 (AUC: 0.923), AKR1C1 (AUC: 0.810), ENPP6 (AUC: 0.882), MAP1B (AUC: 0.862), RHOBTB3 (AUC: 0.861). These results also confirmed the high reliability and accuracy of our model (Fig. 8).

### DEG identification of IRF8

**Fig. 9:**
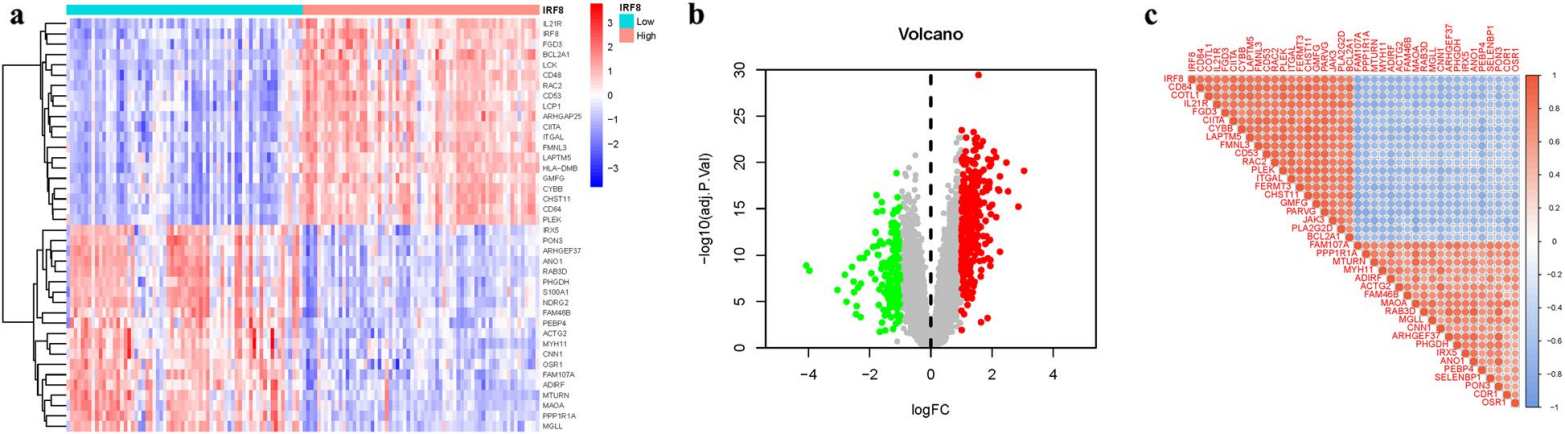
DEG Identification of IRF8. **a** Heatmap. **b** Volcano map. **c** Correlation matrix diagram

By differential analysis of single gene targets, we identified 507 DEGs. Among the 507 DEGs, some DEGs were found to be significantly different. In addition, some genes cluster in the high group and some in the low group. High: IL21R, IRF8, FGD3, BCL2A1, LCK, CD48, RAC2, CD53, etc. Low: IRX5, PON3, ARHGEF37, ANO1, RAB3D, PHGDH, S100A1, etc. (Fig. 9a-b). In addition, we constructed a correlation matrix plot related to IRF8 (Fig. 9c) (Table.S4).

### Enrichment analysis of DEGs of IRF8

**Fig. 10:**
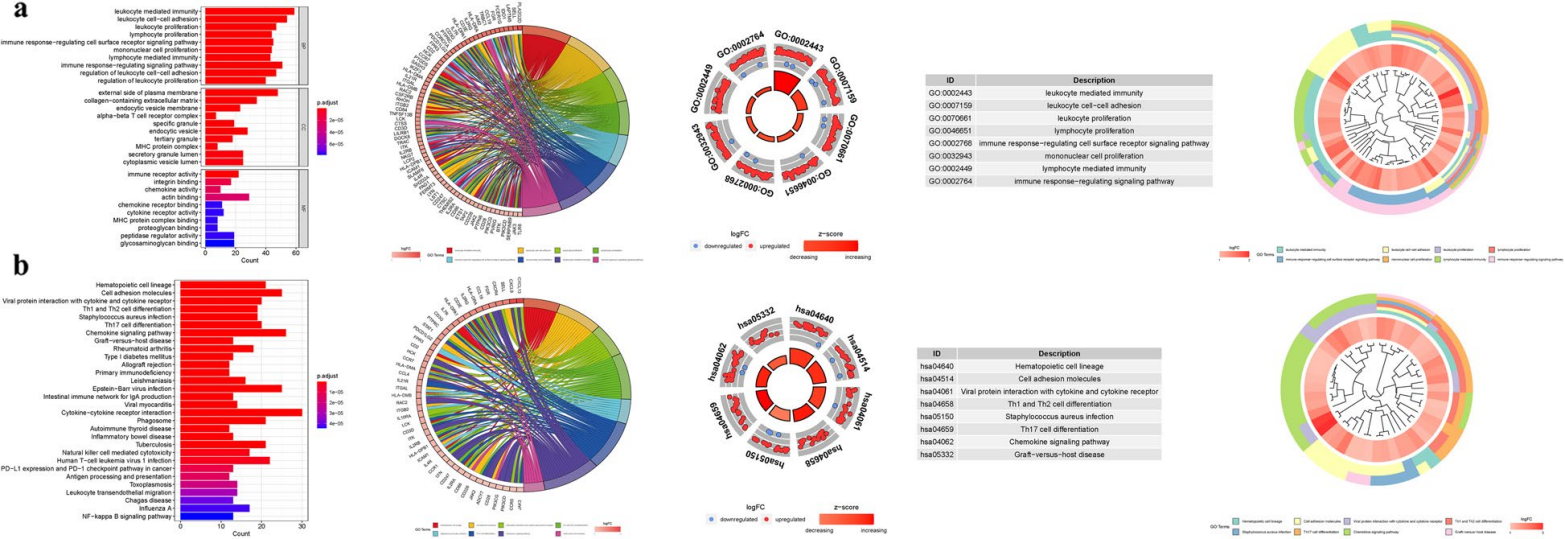
For PMGs, GO, and KEGG analyses were performed. **a** The GO circle illustrates the barplot, chord, circos, and cluster of the selected gene's logFC. **b** The KEGG barplot, chord, circos, and cluster illustrates the scatter map of the logFC of the indicated gene

GO enrichment analysis revealed 996 core targets, including BP, MF, and CC. The MF mainly involves in actin binding (GO:0003779), receptor ligand activity (GO:0048018), immune receptor activity (GO:0140375). The CC mainly involves in external side of plasma membrane (GO:0009897), collagen-containing extracellular matrix (GO:0062023), endocytic vesicle (GO:0030139). The BP mainly involves in leukocyte mediated immunity (GO:0002443), leukocyte cell-cell adhesion (GO:0007159), negative regulation of immune system process (GO:0002683). KEGG enrichment analysis revealing that the over-expressed genes were mainly involved in Cytokine-cytokine receptor interaction (hsa04060), Chemokine signaling pathway (hsa04062), Cell adhesion molecules (hsa04514) (Fig. 10 and Table.S5a-b).

### GSEA of analysis

**Fig. 11:**
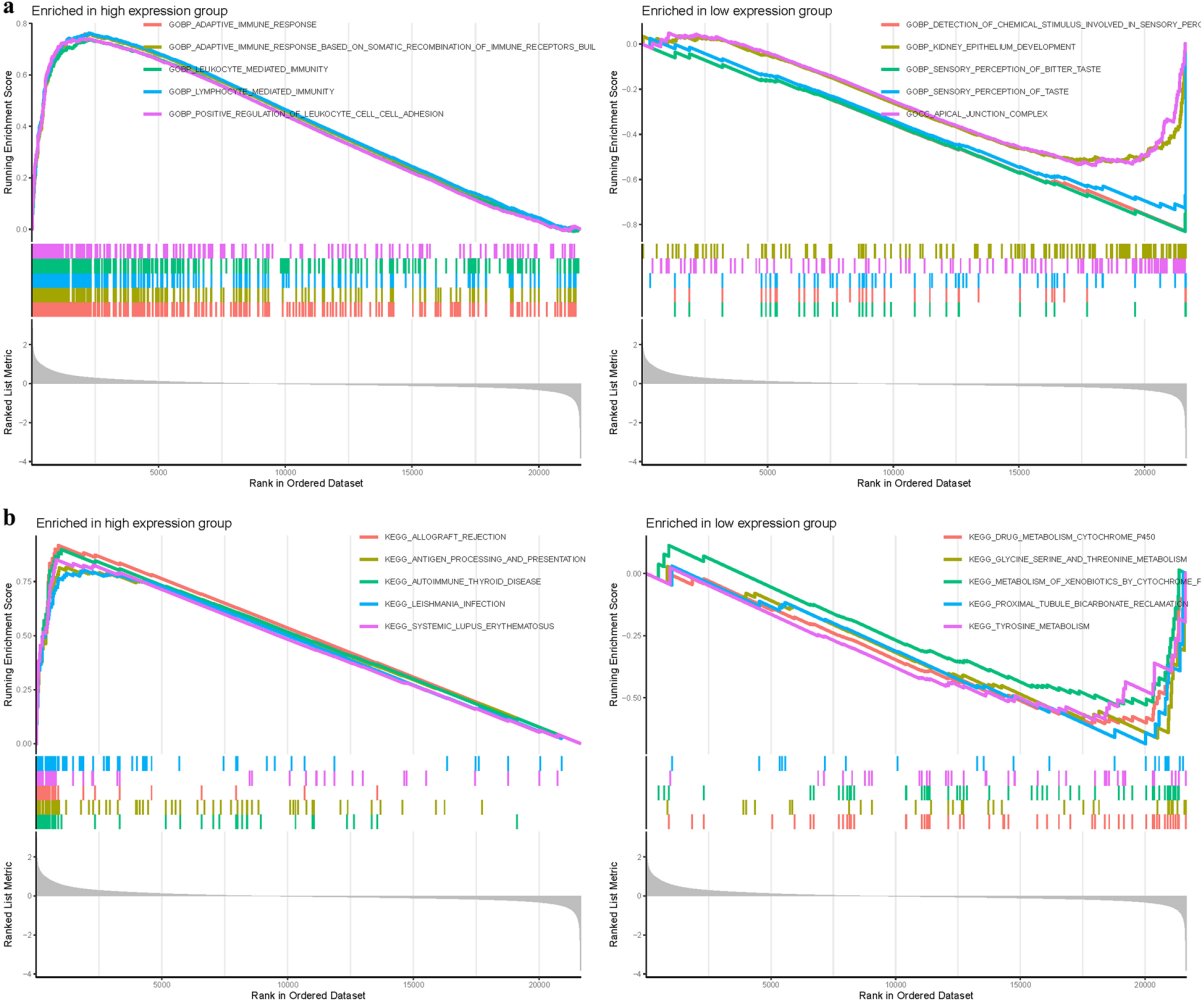
GSEA of Analysis in PDE4B and PDE6D. **a** GO. **b** KEGG

GSEA was deployed to identify functional alterations across the DEGs of IRF8. In high expression group of GO analysis, the functional enrichment mainly involves in BP lymphocyte mediated immunity, BP leukocyte mediated immunity, BP adaptive immune response. In low expression group of GO analysis, the functional enrichment mainly involves in BP sensory perception of bitter taste, BP detection of chemical stimulus involved in sensory perc, BP sensory perception of taste (Fig. 11a).

In high expression group of KEGG analysis, the functional enrichment mainly involves in proximal tubule bicarbonate reclamation, drug metabolism cytochrome p450, glycine serine and threonine metabolism. In low expression group of KEGG analysis, the functional enrichment mainly involves in allograft rejection, autoimmune thyroid disease, systemic lupus erythematosus (Fig. 11b) (Table.S6)

### GSVA of analysis

**Fig. 12:**
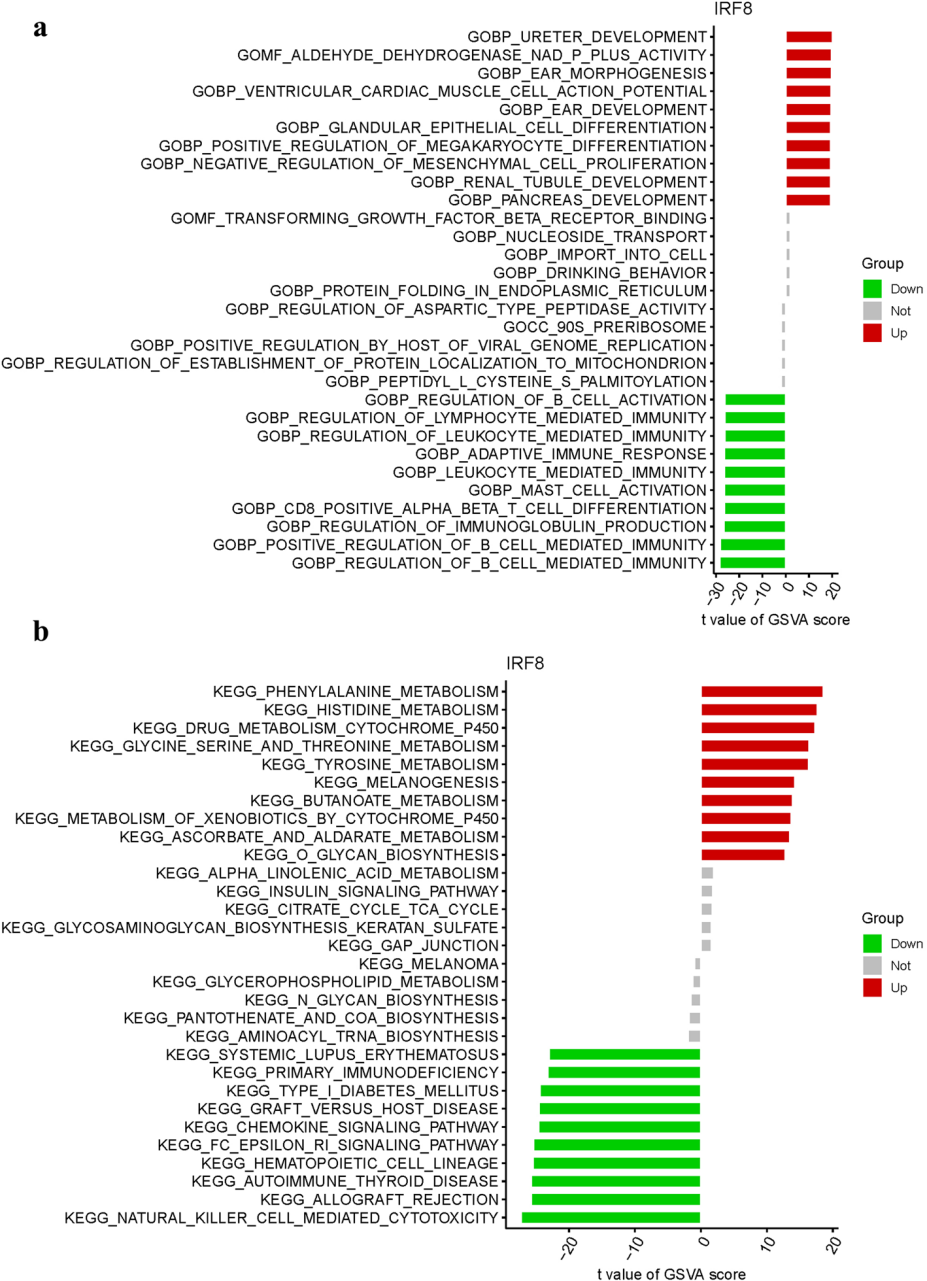
GSVA of Analysis in IRF8. **a** GO. **b** KEGG

GSVA was deployed to identify functional alterations across the DEGs of IRF8. In the GO analysis, the functional enrichment mainly involves in BP ureter development, MF aldehyde dehydrogenase nad p plus activity, BP ear morphogenesis, MF transforming growth factor beta receptor binding, CC 90s preribosome (Fig. 12a). In the KEGG analysis, the functional enrichment mainly involves in phenylalanine metabolism, histidine metabolism, drug metabolism cytochrome p450, glycine serine and threonine metabolism (Fig. 12b) (Table.S7).

### Immune landscape characterization

**Fig. 13:**
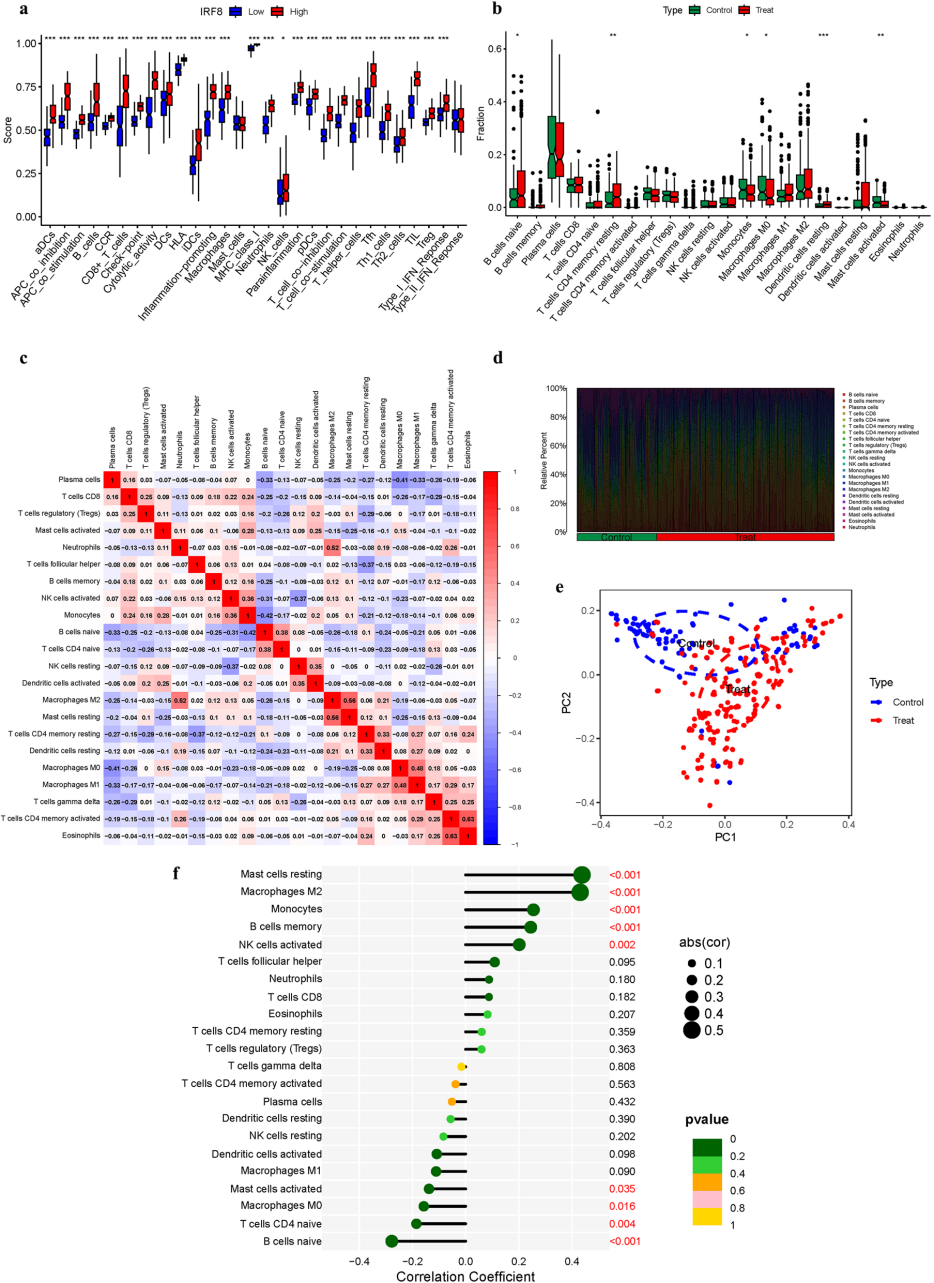
Immune Landscape Characterization. **a** Expression of immune function. **b** Expression of immune cells (**c**) Correlation rectangle plot. **d** Heatmap. **e** PCA analysis. **f** The expression patterns of Correlation Coefficient

**Fig. 14:**
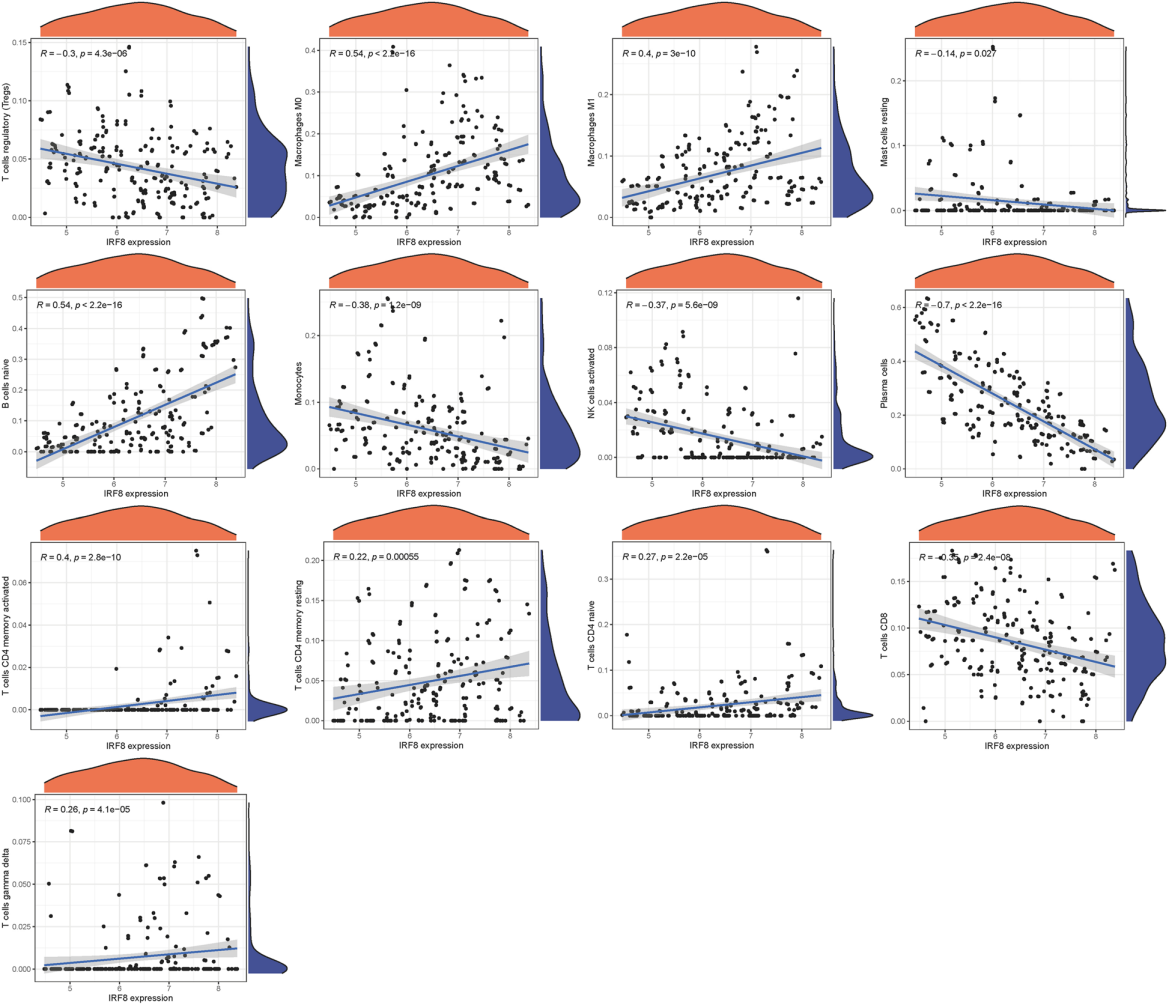
Immune infiltration analyses

The immunological environment has a critical role in the initiation and progression of NSOI. Intriguingly, the risk-associated profiles displayed stark differences in immune cell infiltration. Within the IRF8 cohort, aDCs, APC co inhibition, APC co stimulation, B cells, CCR, CD8+ T cells showed significant variance between the low and high-risk groups. While, Mast cells showed no significant variance between the low and high-risk groups (*P*>0.05) (Fig. 13a). In immune cell, B cells naive, T cells CD4 memory resting, and Dendritic cells resting were highly expressed in the treat group. While, Monocytes, Macrophages M0, and Mast cells activated were highly expressed in the Control group (Fig. 13b). In addition, we also constructed an immune infiltration correlation rectangle plot and heatmap (Fig. 13c-d). Through PCA analysis, immune-based patient categorization was again successfully executed (Fig. 13e). A Lollipop was created to display the expression patterns of Correlation Coefficient. Mast cells resting, Macrophages M2, Monocytes, B cells memory, NK cells activated (Fig. 13f). B cells naive, Macrophages M0, Macrophages M1, T cells CD4 memory activated, T cells CD4 memory resting, T cells CD4 naive, and T cells gamma delta were shown to be positively associated with IRF8. While, Mast cells resting, Monocytes, NK cells activated, Plasma cells, T cells CD8, and T cells regulatory (Tregs) were shown to be negatively linked with IRF8 (Fig. 14) (Table.S7).

### Identification of common RNAs and construction of miRNAs-LncRNAs shared genes network

**Fig. 15:**
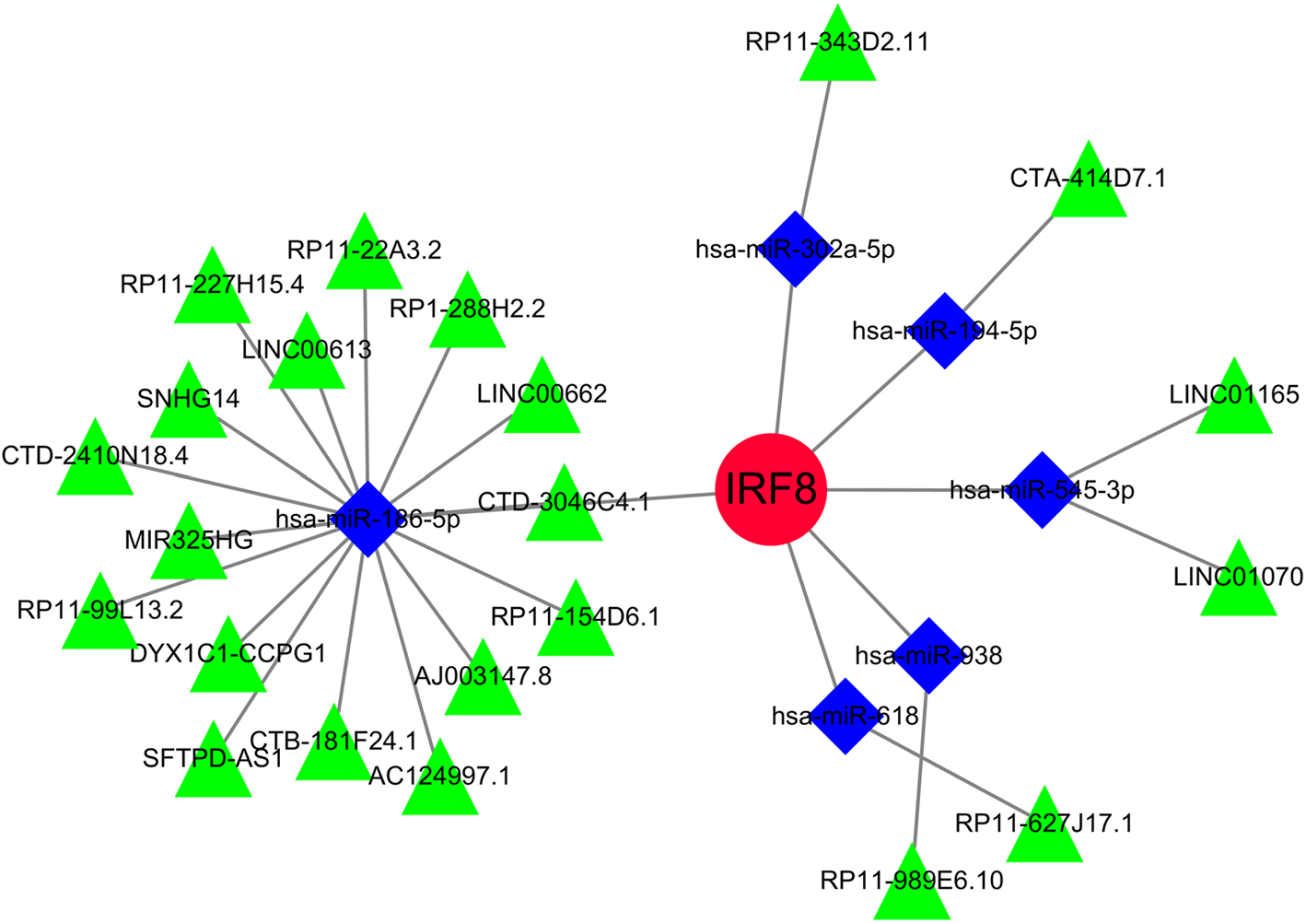
miRNAs-LncRNAs shared Genes Network. Note: Red circles are mrnas, blue quadrangles are miRNAs, and green triangles are lncRNAs

Three databases were searched for 30 miRNAs and 23 lncRNAs linked with NSOI (Table.S7a-b). The network of miRNAs-lncRNAs-genes was constructed by taking the intersection of them and shared genes (obtained by Lasso regression and SVM-RFE). Finally, the miRNAs-genes network included 22 lncRNAs (CTA-414D7.1, LINC01070, RP11-99L13.2, MIR325HG, LINC01165, LINC00613, DYX1C1-CCPG1, RP11-343D2.11, RP11-154D6.1, RP11-22A3.2, SFTPD-AS1, RP1-288H2.2, AC124997.1, CTD-2410N18.4, AJ003147.8, CTD-3046C4.1, RP11-227H15.4, RP11-989E6.10, LINC00662, CTB-181F24.1, RP11-627J17.1, SNHG14), 6 miRNAs (hsa-miR-545-3p, hsa-miR-618, hsa-miR-194-5p, hsa-miR-938, hsa-miR-186-5p, hsa-miR-302a-5p) (Fig. 15) (Table.S8).

## Discussions

NSOI, commonly referred to as orbital pseudotumor, constitutes 8% to 11% of all orbital masses, predominantly affecting middle-aged women. NSOI often leads to recurrent and refractory symptoms despite its generally benign nature, posing significant management challenges [32]. This idiopathic condition, linked to infections, autoimmune and systemic disorders, pharmaceuticals, environmental influences, and individual predispositions, complicates clinical understanding and treatment. Additionally, systemic factors such as upper respiratory infections, sinusitis, and vaccinations have been implicated in NSOI's incidence [33]. Particular viruses like Epstein-Barr virus (EBV), herpes simplex virus (HSV), and SARS-CoV-2 may precipitate NSOI manifestations like dacryoadenitis, which could escalate despite corticosteroid therapy [34]. In immunocompromised patients, such as those with HIV, orbital myositis may develop via immune reconstitution inflammatory syndrome, characterized by a T-cell-mediated inflammatory response. EBV is frequently associated with severe lymphocytic infiltration in NSOI, underscoring its prominent role in the disease's virology [35]. Clinically, NSOI presents with diverse symptoms ranging from extensive orbital inflammation to localized afflictions of structures like the lacrimal gland and extraocular muscles [36]. Symptoms can manifest rapidly or develop gradually and may evolve into chronic conditions with episodic relapses. Common signs include eyelid erythema, edema, ptosis, conjunctival erythema, chemosis, and a characteristic deep, worsening pain with eye movement, suggesting extraocular muscle involvement [36]. This inflammation may restrict muscle movement, leading to ophthalmoplegia and diplopia. Proptosis can occur swiftly or progressively, impairing vision through mechanisms like exposure keratopathy from severe proptosis, optic nerve compression, or potentially in severe cases, as part of a compartment syndrome or via posterior scleritis with exudative retinal detachment [5]. In less pronounced cases, imaging techniques may inadvertently detect orbital masses, facilitating the diagnosis of NSOI. Dacryoadenitis, accounting for half of the NSOI cases, typically presents as a painful, palpable mass on the upper eyelid's lateral aspect, possibly causing an S-shaped ptosis and may appear bilaterally [37].

The IRF family, comprised of nine integral members in mammals, plays a pivotal role in orchestrating a plethora of regulatory functions within the immune system, engaging in both diverse and intersecting molecular pathways [38]. Notably, the expression of IRF8 is recognized to be transcriptionally inducible by IFN-g. In this context, Jia and colleagues discerned that LPS instigated a swift augmentation of IRF8 protein expression in both RAW264.7 cells and peritoneal macrophages [39]. Their investigation further corroborated that stimulation with LPS precipitated an elevated expression of IRF8 mRNA. An accumulating body of evidence posits that the acetylation of IRF family transcription factors is indispensable for optimizing their transcriptional activity [40]. The acetylation across various domains of IRF members induces a conformational alteration, thereby influencing their DNA-binding capacities. Intriguingly, antecedent research has unveiled that SIRT1 possesses the capability to modulate the acetylation status of IRF1 in dendritic cells, consequently inhibiting IL-27p28 subunit expression by attenuating IRF1 transcriptional activity [41]. In instances where the expression of SIRT1 was manipulated in RAW264.7 cells via lentivirus, a discernible alteration in IRF8 expression subsequent to LPS treatment was conspicuously absent [42]. Such findings intimate a plausible regulatory relationship between IRF8 expression and SIRT1, adding a nuanced layer to our understanding of the intricate interplay within the immune regulatory network [43]. Therefore, a nuanced understanding of the role IRF plays in NSOI could not only shed light on its molecular etiology but also offer innovative therapeutic avenues for this poorly understood condition.

Within the multifaceted landscape of NSOI, our multidimensional analytical methodology discerned 507 DEGs. Utilizing a synergistic strategy amalgamating Lasso regression and SVM-RFE, we meticulously isolated a subset of pivotal DEGs intimately associated with NSOI. Subsequent refinement via cross-over analysis elucidated a cadre of 15 hub genes, specifically HLF, PGM1, GPR146, IRF8, TNS1, PLA2G16, PALMD, CCL4, IGK, CORO2B, IGSF10, AKR1C1, ENPP6, MAP1B, and RHOBTB3. Verification against ancillary datasets corroborated their diagnostic propensity, implicating these genes within the intricate mechanistic matrix of NSOI etiology. It is imperative, however, to accentuate that present findings do not conclusively tether these discerned genes to the modulation of specific transcriptional regulators germane to NSOI. Yet, among the cataloged hub genes, IRF8 surfaced as a particularly salient candidate, attracting discerning scrutiny owing to its antecedently recognized roles in inflammation and immune responses. The demarcation of these hub genes sheds light upon prospective pathways for impending scientific exploration. Nevertheless, a more nuanced comprehension of their regulatory networks is indispensable for decoding the enigmatic molecular apparatus underpinning NSOI.

Macrophages epitomize a diverse conglomerate of immune cells, pivotal for initiating and resolving inflammation instigated by pathogenic incursion or tissue damage [44]. These cells exhibit pronounced plasticity, enabling dynamic alterations in their function and physiology in reaction to cytokines and microbial stimuli [45]. Such modifications can engender a spectrum of cells with divergent functions, phenotypically distinguished by the synthesis of both pro-inflammatory and anti-inflammatory cytokines [46]. The gene Sirt2 was initially identified in yeast, functioning as a transcriptional repressor. Its mammalian counterparts, the sirtuins, constitute a family encompassing seven members, denoted as SIRT1 through SIRT7 [47]. Notably, pharmacological potentiation of SIRT1 demonstrates notable influence in this context. IRF8, a constituent of the IRF family, exhibits expression confined to the hematopoietic system [48]. Contrasting the prevalent dichotomy segregating transcription factors implicated in developmental processes from those involved in environmental responses, IRF8 is indispensable for both macrophage differentiation and the stimulus-induced expression of several imperative immune response genes, including but not limited to IL12p40 and IFN-b [49]. This highlights the multifaceted role of IRF8 in both the developmental and environmental response spectra, underscoring its significance in the complex interplay of immune responses [50]. Understanding the regulatory architecture of inflammatory responses in both retinal and extra-retinal tissues is crucial. Our research underscores the significance of DEGs, particularly IRF8, within the pathophysiological landscape of NSOI. Data from the GSE105149 study suggest that IRF8 could serve as a valuable biomarker for NSOI, pointing towards promising directions for future research. Despite these advancements, studies that elucidate the genomic alterations associated with IRF8 are remarkably limited, underscoring a significant gap in the current research landscape. This deficiency highlights the need for more detailed investigations to better understand the molecular mechanisms by which IRF8 influences NSOI, thereby providing a foundation for targeted therapeutic interventions

Within the intricate realm of NSOI, burgeoning evidence contends the traditional perspective that solely attributes the amplified immune response to CD4 cell activity. Instead, a nuanced spectrum of pre-existing T-regulatory cells and a dichotomy of pro-inflammatory and regulatory entities, such as cytokine imbalances, seem to be at play [51]. This sophisticated environment potentially predisposes the immune system to dysregulation, rendering it vulnerable to a spectrum of opportunistic infections, be they manifest, latent, or previously controlled [52]. Conditions including Tuberculosis, Cytomegalovirus infections, Progressive Multifocal Leukoencephalopathy, Kaposi's sarcoma, and assorted autoimmune disorders may either amplify or escape detection, with Cytomegalovirus retinitis being notably predominant in association with Immunological Recovery Uveitis [53, 54]. Intriguingly, nascent therapeutic avenues targeting the elevation of intracellular cAMP levels are demonstrating potential to mitigate chronic inflammation. Small-molecule PDE4 inhibitors, which forestall cAMP degradation, have showcased efficacy across a myriad of inflammatory conditions, encompassing Inflammatory Bowel Disease, Atopic Dermatitis, and Rheumatoid Arthritis [55, 56]. Building upon our preceding explorations, we meticulously examined the expression profile of IRF8 within the immunological microenvironment, identifying, through a Lollipop plot, associations with various immune cells (Fig. 13f). B cells naive, Macrophages M0, Macrophages M1, T cells CD4 memory activated, T cells CD4 memory resting, T cells CD4 naive, and T cells gamma delta were shown to be positively associated with IRF8. While, Mast cells resting, Monocytes, NK cells activated, Plasma cells, T cells CD8, and T cells regulatory (Tregs) were shown to be negatively linked with IRF8. The intricate dance between IRF8 and a diversity of immunological cell types accentuates the central role of inflammation and immune responses in the pathophysiology of NSOI, providing a foundation for the development of targeted therapeutic interventions.

Venturing into the relatively uncharted interface between biomarkers and NSOI, our research constitutes a groundbreaking addition to an emerging, yet rapidly evolving field. While existing literature harnesses bioinformatics to reveal correlations between immunization and ocular diseases, a palpable void remains in relation to IRF8 in the context of NSOI [32, 57, 58]. Significant contributions include the works of Liu et al., and Hu et al., who applied advanced analytical methods to identify key genes in NSOI and thyroid eye disease, respectively, and Huang et al., who coupled exhaustive bioinformatics with in vivo validation to discern pivotal genes in diabetic retinopathy. Diverging from established norms, our investigation adopts an innovative metabolic cellular framework to inform therapeutic strategies for NSOI, applying pioneering methodologies absent in previous research. The role of IRF8 in NSOI marks a significant advance in our understanding of its pathophysiology. As a critical transcription factor involved in immune regulation and myeloid lineage differentiation, IRF8 has emerged as a pivotal element in distinguishing NSOI from other orbital inflammations. The modulation of IRF8 could serve not only as a biomarker for early detection but also provide crucial prognostic insights into disease progression and therapeutic responses. The ability to monitor IRF8 expression levels could revolutionize NSOI diagnostics, moving from a predominantly exclusion-based approach fraught with clinical uncertainties to one that is precise and biomarker-driven. This would allow for more accurate and timely diagnosis, reducing the current dependence on subjective clinical judgment. Additionally, elevated IRF8 levels could predict disease activity and guide the customization of treatment plans, which is critical given the chronic and relapsing nature of NSOI and the significant side effects associated with prolonged corticosteroid use. Therapeutically, targeting IRF8 could introduce novel treatment modalities for NSOI by modulating key inflammatory pathways more selectively than current broad-spectrum immunosuppressive approaches. This strategy promises treatments with reduced side effects and enhanced patient compliance, potentially improving clinical outcomes significantly. The clinical implications of using IRF8 as both a biomarker and a therapeutic target in NSOI are profound. Future research should concentrate on defining the specific mechanisms through which IRF8 influences NSOI, utilizing advanced genomic and proteomic techniques to map its interactions within orbital tissue and identify influenced secondary targets. Such studies should aim to validate IRF8's clinical relevance through larger, multicentric trials and investigate targeted therapeutic agents that modulate IRF8 activity. Despite the promising theoretical and methodological advancements presented, this study recognizes existing limitations, particularly in understanding the foundational mechanistic pathways involving IRF8. Both in vivo and in vitro models present viable paths for further investigation, though their full potential and implications within the broader context of NSOI research are yet to be fully realized.

## Conclusions

The study ventures into the complex domain of oncological diversity, focusing on the role of IRF8 within the immune inflammation spectrum, thereby highlighting its extensive prognostic relevance. Utilizing advanced predictive modeling, we have meticulously delineated the transcriptional framework of IRF8, identifying significant variations in expression between NSOI and normal tissues. This analysis positions IRF8 as a crucial prognostic marker in NSOI, characterized by a diverse array of genetic alterations—including mutations, duplications, and amplifications—that define this immune-inflammatory condition. Importantly, our research reveals a strong correlation between IRF8 expression levels and the degree of immune cell infiltration within the immune microenvironment. This finding not only enhances the prognostic accuracy of IRF8 but also underscores its potential as a diagnostic tool for evaluating the efficacy of various immunotherapeutic strategies in the heterogeneous contexts of NSOI.

### Supplementary Information

Supplementary Material 1. 

## Data Availability

No datasets were generated or analysed during the current study.

## Abbreviations

NSOI: Nonspecific orbital inflammation
GO: Gene Ontology
TCM: Traditional Chinese medicine
MF: Molecular functions
KEGG: Kyoto Encyclopedia of Genes and Genomes
GEO: Gene Expression Omnibus
IRF8: Interferon Regulatory Factor 8
BP: Biological processes
CC: Cellular components
DEGs: Differentially Expressed Genes
