# Supplementary material for: The roles of IRF8 in nonspecific orbital inflammation: an integrated analysis by bioinformatics and machine learning
Source: J Ophthalmic Inflamm Infect. 2024 Jun 20;14:29. doi: 10.1186/s12348-024-00410-4 (PMC11190126; doi:10.1186/s12348-024-00410-4)
Supplement: Supplementary file 1 — Supplementary Material 1. [file 12348_2024_410_MOESM1_ESM.doc]

**The roles of IRF8 in Nonspecific Orbital Inflammation: An integrated analysis by Bioinformatics and Machine Learning**

**Supplementary appendix to the manuscript**

Contents of supplementary appendix

[Appendix 1 3](#__RefHeading___Toc11146)

[The clinical characteristics of patients 3](#__RefHeading___Toc29786)

[Table S1. The clinical characteristics of patients. 3](#__RefHeading___Toc15538)

[Appendix 2 4](#__RefHeading___Toc26373)

[DEGs linked to NSOI 4](#__RefHeading___Toc23276)

[Table S2. 314 DEGs linked to NSOI. 4](#__RefHeading___Toc28614)

[Appendix 3 12](#__RefHeading___Toc17404)

[Table S3a. LASSO genes. 12](#__RefHeading___Toc8634)

[Table S3b. SVM-RFE genes. 12](#__RefHeading___Toc332)

[Table S3c. InterGenes. 13](#__RefHeading___Toc8291)

[Appendix 4 13](#__RefHeading___Toc20780)

[DEG Identification of IRF8 13](#__RefHeading___Toc32339)

[Table S4. 507 DEG Identification of IRF8 13](#__RefHeading___Toc15171)

[Appendix 5 48](#__RefHeading___Toc28280)

[Table S5a. Analysis of GO. 48](#__RefHeading___Toc12605)

[Table S5b. Analysis of KEGG. 97](#__RefHeading___Toc17520)

[Appendix 6 102](#__RefHeading___Toc26174)

[Table 6a. GO of GSEA analysis. 102](#__RefHeading___Toc25805)

[Table 6b. KEGG of GSEA analysis. 183](#__RefHeading___Toc6824)

[Appendix 7 190](#__RefHeading___Toc3125)

[Table 7. Immune Correlation Analysis. 190](#__RefHeading___Toc18213)

[Appendix 8 192](#__RefHeading___Toc19645)

[Table 8a. Gene-miRNA. 192](#__RefHeading___Toc10537)

[Table 8b. Gene-miRNA. 193](#__RefHeading___Toc23290)

# Appendix 1

**The clinical characteristics of patients**

**Table S1.** **The clinical characteristics of patients.**

| GSE58331 | | GSE105149 | |
| --- | --- | --- | --- |
| Variables | Number of samples | Variables | Number of samples |
| Gender |  | Gender |  |
| Male/Female | 19/56 | Male/Female | 9/18 |
| Diagnosis |  | Diagnosis |  |
| NSOI/Normal | 75/29 | NSOI/Normal | 27/7 |
| Tissue |  | Tissue |  |
| Anterior Orbit/  Lacrimal gland | 33/42 | Anterior Orbit/  Lacrimal gland | 0/27 |

# Appendix 2

## **DEGs linked to NSOI**

**Table S2. 314 DEGs linked to NSOI.**

| id | logFC | AveExpr | t | P.Value | adj.P.Val |
| --- | --- | --- | --- | --- | --- |
| HLF | -1.581770516 | 4.486953656 | -12.4363579 | 6.18E-26 | 1.34E-21 |
| MGST1 | -1.73101078 | 4.846522249 | -10.96290951 | 1.11E-21 | 7.99E-18 |
| C2orf40 | -2.196342714 | 4.519571821 | -10.79956686 | 3.24E-21 | 1.75E-17 |
| ADH1B | -2.095662677 | 4.158273041 | -10.74506249 | 4.64E-21 | 2.01E-17 |
| PGM1 | -1.196129698 | 5.644288109 | -10.66251442 | 7.97E-21 | 2.88E-17 |
| PGRMC2 | -1.258252644 | 4.200521305 | -10.61694307 | 1.07E-20 | 2.91E-17 |
| GSTM3 | -1.207615154 | 4.74005161 | -10.58382151 | 1.33E-20 | 3.21E-17 |
| NTRK2 | -1.228056221 | 4.525433831 | -10.52344673 | 1.98E-20 | 4.29E-17 |
| TGFBR3 | -1.886004737 | 5.252480398 | -10.44841687 | 3.23E-20 | 6.36E-17 |
| PPP1R1A | -2.187117989 | 5.727381115 | -10.11887501 | 2.75E-19 | 4.50E-16 |
| RNASE4 | -1.718212377 | 5.221705294 | -10.1073842 | 2.97E-19 | 4.50E-16 |
| APCDD1 | -1.512789702 | 5.715749609 | -10.09983731 | 3.11E-19 | 4.50E-16 |
| CAB39L | -1.030217143 | 4.949170449 | -10.08187051 | 3.50E-19 | 4.73E-16 |
| SLC24A3 | -1.125513094 | 4.313704729 | -10.01079712 | 5.54E-19 | 7.05E-16 |
| WLS | -1.149021225 | 5.203113407 | -9.907575363 | 1.08E-18 | 1.23E-15 |
| TUSC1 | -1.287879465 | 5.651620298 | -9.767610369 | 2.65E-18 | 2.87E-15 |
| STXBP1 | -1.412261334 | 4.671545908 | -9.680158969 | 4.64E-18 | 4.37E-15 |
| FAXDC2 | -1.056469312 | 4.96290536 | -9.62374166 | 6.66E-18 | 5.54E-15 |
| C1orf198 | -1.118940653 | 6.317302602 | -9.609476425 | 7.29E-18 | 5.54E-15 |
| NGFRAP1 | -1.325144028 | 6.64221447 | -9.607476998 | 7.38E-18 | 5.54E-15 |
| SEMA3G | -1.553875288 | 5.80514183 | -9.606545263 | 7.43E-18 | 5.54E-15 |
| TCIRG1 | 1.013294599 | 7.003690562 | 9.601458778 | 7.67E-18 | 5.54E-15 |
| PLIN1 | -3.510729005 | 6.206463925 | -9.564801157 | 9.70E-18 | 6.77E-15 |
| TIMP4 | -1.776944016 | 3.777321203 | -9.540191143 | 1.13E-17 | 7.44E-15 |
| GPAM | -1.964251439 | 3.992002302 | -9.52302724 | 1.27E-17 | 7.80E-15 |
| CDR1 | -2.144905233 | 6.179538535 | -9.519141461 | 1.30E-17 | 7.80E-15 |
| NECAB1 | -1.034647256 | 2.169721244 | -9.502402166 | 1.44E-17 | 8.09E-15 |
| LEPR | -2.206002435 | 3.940087912 | -9.496928317 | 1.49E-17 | 8.09E-15 |
| CAV2 | -1.337869546 | 4.75307031 | -9.47127428 | 1.76E-17 | 9.28E-15 |
| GHR | -2.038120615 | 3.529538566 | -9.433388079 | 2.24E-17 | 1.03E-14 |
| MEST | -2.032412974 | 3.995469591 | -9.413087554 | 2.55E-17 | 1.15E-14 |
| NDNF | -1.929794836 | 4.293116528 | -9.351750842 | 3.76E-17 | 1.66E-14 |
| HADH | -1.089676098 | 4.925785437 | -9.320925836 | 4.56E-17 | 1.94E-14 |
| IGFBP6 | -1.895419766 | 6.719933425 | -9.308710627 | 4.93E-17 | 2.05E-14 |
| OLFML2A | -1.419877286 | 5.287243312 | -9.288056282 | 5.62E-17 | 2.30E-14 |
| PRDX6 | -1.024201576 | 6.82029148 | -9.257464531 | 6.82E-17 | 2.59E-14 |
| ACACB | -1.225419329 | 6.038983591 | -9.242358839 | 7.50E-17 | 2.80E-14 |
| MTURN | -1.222611145 | 5.378674298 | -9.233041454 | 7.95E-17 | 2.92E-14 |
| CPE | -1.257137939 | 4.715130972 | -9.215894478 | 8.86E-17 | 3.14E-14 |
| TRHDE-AS1 | -1.518368089 | 3.078249473 | -9.190156933 | 1.04E-16 | 3.58E-14 |
| IGSF10 | -1.151645472 | 4.12122447 | -9.179030936 | 1.12E-16 | 3.78E-14 |
| LIMD2 | 1.096609514 | 7.559429116 | 9.162129238 | 1.24E-16 | 4.14E-14 |
| NPR3 | -1.532985416 | 3.501358447 | -9.113191883 | 1.69E-16 | 5.46E-14 |
| LOC284825 | -1.372371338 | 3.039392288 | -9.088822552 | 1.97E-16 | 6.27E-14 |
| PPAP2B | -1.546183631 | 6.72891155 | -9.078431504 | 2.10E-16 | 6.60E-14 |
| ENPP6 | -1.20312907 | 2.732761455 | -8.99736236 | 3.49E-16 | 1.03E-13 |
| NFIA | -1.027794466 | 4.963336742 | -8.980603043 | 3.88E-16 | 1.12E-13 |
| PBX1 | -1.44655793 | 6.592959362 | -8.95283287 | 4.61E-16 | 1.23E-13 |
| EFHD1 | -1.347095261 | 6.250872915 | -8.947018061 | 4.78E-16 | 1.24E-13 |
| TMEM100 | -1.245035632 | 2.621077458 | -8.93205661 | 5.25E-16 | 1.31E-13 |
| SORBS1 | -1.16278045 | 5.510134543 | -8.931606383 | 5.27E-16 | 1.31E-13 |
| MAOA | -1.958420887 | 5.489570158 | -8.921310044 | 5.61E-16 | 1.38E-13 |
| ADIPOQ | -3.045245553 | 3.959666935 | -8.881446745 | 7.20E-16 | 1.73E-13 |
| CORO2B | -1.167529165 | 4.368560636 | -8.879327674 | 7.29E-16 | 1.73E-13 |
| FTO | -1.022722841 | 7.177143322 | -8.877744447 | 7.36E-16 | 1.73E-13 |
| RUNX1-IT1 | 1.129771198 | 5.125247074 | 8.864406406 | 8.00E-16 | 1.84E-13 |
| ADIRF | -1.745871197 | 8.12664587 | -8.844058477 | 9.08E-16 | 2.05E-13 |
| ITGB2-AS1 | 1.227307212 | 5.904472474 | 8.841717787 | 9.21E-16 | 2.06E-13 |
| FHL1 | -2.092149128 | 6.045253917 | -8.816132924 | 1.08E-15 | 2.36E-13 |
| RNF11 | -1.535841555 | 3.935163673 | -8.800732561 | 1.19E-15 | 2.57E-13 |
| CHRDL1 | -1.698377913 | 5.787419576 | -8.684949938 | 2.43E-15 | 5.11E-13 |
| FABP4 | -2.353748214 | 5.206550928 | -8.682245195 | 2.47E-15 | 5.15E-13 |
| PLSCR4 | -1.274757896 | 2.985046822 | -8.655960692 | 2.91E-15 | 5.78E-13 |
| TNMD | -1.04518328 | 2.791647023 | -8.650148707 | 3.01E-15 | 5.93E-13 |
| SH3D19 | -1.22465157 | 5.719367165 | -8.627295814 | 3.47E-15 | 6.71E-13 |
| PHYH | -1.291005106 | 4.511931621 | -8.615465443 | 3.73E-15 | 7.09E-13 |
| PCDH18 | -1.299308935 | 5.42226592 | -8.559750062 | 5.26E-15 | 9.73E-13 |
| TBC1D10C | 1.473425607 | 6.577682583 | 8.558349206 | 5.30E-15 | 9.73E-13 |
| EPHX1 | -1.206806765 | 7.467876504 | -8.552855759 | 5.48E-15 | 9.89E-13 |
| PPAP2A | -1.136882346 | 5.91326828 | -8.544351478 | 5.78E-15 | 1.03E-12 |
| PCOLCE2 | -1.515874871 | 3.030533398 | -8.542001332 | 5.86E-15 | 1.03E-12 |
| CIDEC | -2.41117819 | 5.944620287 | -8.535646536 | 6.09E-15 | 1.06E-12 |
| KLB | -1.119367337 | 2.353027435 | -8.532625108 | 6.21E-15 | 1.08E-12 |
| LYVE1 | -2.244951922 | 5.215787478 | -8.524917355 | 6.51E-15 | 1.10E-12 |
| ASPA | -1.055274718 | 3.669429502 | -8.523573255 | 6.56E-15 | 1.10E-12 |
| MIAT | 1.019026479 | 5.24153807 | 8.519312101 | 6.73E-15 | 1.12E-12 |
| HCAR1 | -1.119209236 | 3.777418405 | -8.469317001 | 9.14E-15 | 1.45E-12 |
| PARVG | 1.094550292 | 5.516760352 | 8.460115639 | 9.67E-15 | 1.51E-12 |
| MAMDC2 | -1.730469482 | 3.118799264 | -8.445980461 | 1.05E-14 | 1.63E-12 |
| HSPB6 | -2.539368644 | 7.497621453 | -8.431807843 | 1.15E-14 | 1.75E-12 |
| CETN2 | -1.087280636 | 5.78650944 | -8.423713089 | 1.21E-14 | 1.82E-12 |
| PPL | -1.710548579 | 6.388411778 | -8.395003875 | 1.44E-14 | 2.09E-12 |
| BNIP3L | -1.051369981 | 6.860025447 | -8.387225216 | 1.51E-14 | 2.18E-12 |
| VIT | -1.675552155 | 4.902205109 | -8.368419053 | 1.69E-14 | 2.43E-12 |
| THRSP | -1.636316122 | 4.64274161 | -8.34165597 | 1.99E-14 | 2.71E-12 |
| PCYOX1 | -1.299089014 | 5.422457266 | -8.341566259 | 1.99E-14 | 2.71E-12 |
| HLA-F | 1.078545985 | 7.814018991 | 8.321710672 | 2.25E-14 | 2.97E-12 |
| YAP1 | -1.111039667 | 5.665259802 | -8.270311849 | 3.07E-14 | 3.93E-12 |
| ACSL1 | -1.319124186 | 6.906560476 | -8.26815685 | 3.11E-14 | 3.96E-12 |
| CNTFR | -1.158708488 | 6.058542253 | -8.199593737 | 4.70E-14 | 5.66E-12 |
| CAP2 | -1.065065275 | 3.657663217 | -8.197426883 | 4.77E-14 | 5.70E-12 |
| ATXN1L | -1.042632367 | 5.41128383 | -8.187837241 | 5.05E-14 | 5.94E-12 |
| IGHM | 1.893953592 | 8.464685567 | 8.16003212 | 5.97E-14 | 6.91E-12 |
| MN1 | -1.570698055 | 5.292246061 | -8.154754024 | 6.16E-14 | 7.10E-12 |
| GSTM5 | -1.25620581 | 4.944432756 | -8.152929983 | 6.23E-14 | 7.14E-12 |
| ACADL | -1.104516836 | 3.410072407 | -8.151645618 | 6.28E-14 | 7.15E-12 |
| TMEM132C | -1.267317386 | 4.067071738 | -8.100494497 | 8.54E-14 | 9.29E-12 |
| SCARA5 | -1.193473518 | 5.890702792 | -8.064299792 | 1.06E-13 | 1.11E-11 |
| PTPRCAP | 1.133661289 | 7.237678373 | 8.048820964 | 1.16E-13 | 1.21E-11 |
| NAPSB | 1.030717746 | 4.947260779 | 8.043680529 | 1.20E-13 | 1.24E-11 |
| OMD | -1.322607942 | 3.923039324 | -8.029980482 | 1.30E-13 | 1.34E-11 |
| MFAP4 | -1.601286777 | 6.91438899 | -8.02214008 | 1.36E-13 | 1.39E-11 |
| ADAM28 | 1.102305993 | 4.784065539 | 8.018397614 | 1.40E-13 | 1.40E-11 |
| RBP4 | -1.174535717 | 4.410902601 | -8.006856523 | 1.50E-13 | 1.48E-11 |
| CALB2 | -1.253426532 | 3.963233847 | -8.003234146 | 1.53E-13 | 1.50E-11 |
| GYG2 | -1.254679436 | 4.610246054 | -7.997454507 | 1.58E-13 | 1.54E-11 |
| PDK4 | -1.426209986 | 4.540221122 | -7.994998707 | 1.60E-13 | 1.55E-11 |
| LRRN4CL | -1.726727881 | 4.424048701 | -7.980499892 | 1.75E-13 | 1.65E-11 |
| AKR1C1 | -1.041371399 | 5.59018636 | -7.972903639 | 1.83E-13 | 1.71E-11 |
| EHBP1 | -1.14554198 | 5.982775699 | -7.953103321 | 2.06E-13 | 1.89E-11 |
| APOD | -1.882244524 | 9.838764755 | -7.927534736 | 2.40E-13 | 2.16E-11 |
| TSKU | -1.150911186 | 5.787482441 | -7.917882808 | 2.54E-13 | 2.27E-11 |
| TRBC1 | 1.815085432 | 6.583157486 | 7.91284724 | 2.62E-13 | 2.32E-11 |
| CAV1 | -1.813912368 | 6.854425452 | -7.90816649 | 2.69E-13 | 2.37E-11 |
| NDFIP1 | -1.112314401 | 6.026818655 | -7.895332306 | 2.90E-13 | 2.52E-11 |
| CRYAB | -1.961025916 | 8.096120564 | -7.893923502 | 2.93E-13 | 2.52E-11 |
| RP11-736K20.5 | -1.151628229 | 5.633634195 | -7.86614755 | 3.45E-13 | 2.92E-11 |
| IGLL3P | 1.348670984 | 9.057507242 | 7.861481713 | 3.55E-13 | 2.99E-11 |
| BC022047 | -1.207117032 | 4.802307286 | -7.846896828 | 3.87E-13 | 3.25E-11 |
| AMOTL2 | -1.25204271 | 5.288509815 | -7.832080887 | 4.22E-13 | 3.48E-11 |
| DENND1C | 1.280791781 | 6.090037587 | 7.829675803 | 4.28E-13 | 3.51E-11 |
| CORO1A | 1.490258951 | 7.766740844 | 7.823698118 | 4.44E-13 | 3.61E-11 |
| ITGAL | 1.261781787 | 5.975292808 | 7.81434834 | 4.69E-13 | 3.79E-11 |
| IL21R | 1.223039408 | 5.004894929 | 7.799184731 | 5.13E-13 | 4.10E-11 |
| LINC00657 | -1.10323622 | 5.357381967 | -7.794978056 | 5.26E-13 | 4.17E-11 |
| SGCG | -1.598499332 | 3.15631333 | -7.782610437 | 5.65E-13 | 4.45E-11 |
| CCL19 | 1.818238625 | 7.880550521 | 7.778598006 | 5.79E-13 | 4.51E-11 |
| TCF7L1 | -1.136132121 | 6.152244124 | -7.771954361 | 6.02E-13 | 4.67E-11 |
| IRF8 | 1.233530672 | 6.102711564 | 7.771393236 | 6.04E-13 | 4.67E-11 |
| FZD4 | -1.034166834 | 5.741148556 | -7.758446742 | 6.52E-13 | 4.99E-11 |
| DNMBP | -1.051034251 | 5.196394381 | -7.75455089 | 6.67E-13 | 5.09E-11 |
| MRAP | -1.018747691 | 3.573749337 | -7.742089943 | 7.18E-13 | 5.36E-11 |
| TSPAN6 | -1.021555172 | 4.345915851 | -7.73172439 | 7.63E-13 | 5.64E-11 |
| PDGFD | -1.357957301 | 4.912871545 | -7.720007191 | 8.17E-13 | 6.02E-11 |
| PAM | -1.112423739 | 5.885799585 | -7.709756918 | 8.68E-13 | 6.34E-11 |
| FMO2 | -1.379755451 | 5.885950942 | -7.703198359 | 9.02E-13 | 6.55E-11 |
| OLFML1 | -1.313114257 | 3.40833212 | -7.70078897 | 9.15E-13 | 6.62E-11 |
| HLA-B | 1.163607639 | 10.93628287 | 7.681355193 | 1.02E-12 | 7.28E-11 |
| MMP9 | 1.965916767 | 6.857354614 | 7.666651644 | 1.12E-12 | 7.70E-11 |
| IL7R | 1.732257154 | 5.609671444 | 7.664092123 | 1.13E-12 | 7.80E-11 |
| TMEM200B | -1.061462554 | 4.505638146 | -7.661038192 | 1.15E-12 | 7.86E-11 |
| AOC3 | -1.977719355 | 4.353420703 | -7.651111705 | 1.22E-12 | 8.25E-11 |
| CD74 | 1.119709874 | 8.834651006 | 7.632957925 | 1.36E-12 | 9.04E-11 |
| CRNDE | -1.293840002 | 4.568632288 | -7.631385291 | 1.37E-12 | 9.09E-11 |
| IL32 | 1.283633762 | 7.748202392 | 7.630424906 | 1.38E-12 | 9.11E-11 |
| ZNF423 | -1.299917264 | 4.837136643 | -7.620270671 | 1.46E-12 | 9.64E-11 |
| TUBB2A | -1.522843019 | 4.519781287 | -7.590925442 | 1.74E-12 | 1.12E-10 |
| LAPTM4A | -1.020146223 | 7.062747291 | -7.585002271 | 1.80E-12 | 1.15E-10 |
| CIITA | 1.434128152 | 5.82955887 | 7.584999067 | 1.80E-12 | 1.15E-10 |
| LCK | 1.207272363 | 5.133001308 | 7.556984982 | 2.12E-12 | 1.33E-10 |
| CA3 | -1.397029769 | 2.871505982 | -7.540561679 | 2.33E-12 | 1.45E-10 |
| CHI3L1 | 1.911130703 | 5.577084066 | 7.540506624 | 2.33E-12 | 1.45E-10 |
| CYBRD1 | -1.32368824 | 6.412982308 | -7.525924649 | 2.54E-12 | 1.57E-10 |
| AQPEP | -1.082296344 | 2.474334542 | -7.522886587 | 2.58E-12 | 1.59E-10 |
| MYOC | -1.426256726 | 4.267750326 | -7.5181086 | 2.65E-12 | 1.62E-10 |
| ALDH1A1 | -1.157776409 | 5.690677596 | -7.500332159 | 2.94E-12 | 1.78E-10 |
| PRELP | -1.368875484 | 6.347271132 | -7.486383941 | 3.19E-12 | 1.89E-10 |
| TRAC | 1.214385275 | 7.322602675 | 7.485462438 | 3.21E-12 | 1.90E-10 |
| MARC1 | -1.092510242 | 4.381581801 | -7.476964151 | 3.37E-12 | 1.97E-10 |
| BNIP3 | -1.235396841 | 5.675988924 | -7.461065531 | 3.69E-12 | 2.15E-10 |
| ITGB2 | 1.165071216 | 6.893631197 | 7.456119994 | 3.80E-12 | 2.20E-10 |
| EBF1 | -1.318238618 | 4.626904283 | -7.449743627 | 3.94E-12 | 2.28E-10 |
| SIGLEC10 | 1.224329637 | 5.535788687 | 7.432731334 | 4.35E-12 | 2.48E-10 |
| CD36 | -1.607165225 | 4.534679053 | -7.422677356 | 4.61E-12 | 2.62E-10 |
| DDR2 | -1.102453345 | 6.035613186 | -7.407888293 | 5.02E-12 | 2.81E-10 |
| ZNF667-AS1 | -1.008788072 | 5.637265115 | -7.393656611 | 5.44E-12 | 3.02E-10 |
| CD34 | -1.256004889 | 6.513814211 | -7.388224299 | 5.62E-12 | 3.10E-10 |
| RHOH | 1.247725263 | 4.641017917 | 7.386803594 | 5.66E-12 | 3.10E-10 |
| USP54 | -1.17571173 | 6.102203696 | -7.38439834 | 5.74E-12 | 3.14E-10 |
| GBE1 | -1.027826069 | 3.210398081 | -7.380737672 | 5.86E-12 | 3.20E-10 |
| IL2RG | 1.749958396 | 7.818285751 | 7.3621074 | 6.53E-12 | 3.51E-10 |
| CFH | -1.350738672 | 5.556135523 | -7.326546868 | 8.00E-12 | 4.18E-10 |
| SLC19A3 | -1.019896884 | 2.904428349 | -7.315742541 | 8.51E-12 | 4.40E-10 |
| TIMP3 | -1.151600079 | 8.030378699 | -7.307362679 | 8.93E-12 | 4.57E-10 |
| MAP1B | -1.006337056 | 5.53842632 | -7.304561661 | 9.07E-12 | 4.63E-10 |
| AOX1 | -1.32271604 | 5.252556404 | -7.296763057 | 9.49E-12 | 4.80E-10 |
| SMIM19 | -1.135827832 | 6.72484084 | -7.291701762 | 9.77E-12 | 4.92E-10 |
| SRPX | -1.158450028 | 6.219664492 | -7.261717257 | 1.16E-11 | 5.66E-10 |
| CD3D | 1.245146874 | 6.29149497 | 7.255452129 | 1.20E-11 | 5.86E-10 |
| PALMD | -1.118219182 | 5.186982933 | -7.252568101 | 1.22E-11 | 5.91E-10 |
| PLA2G2D | 1.941342796 | 5.961330087 | 7.216571198 | 1.50E-11 | 7.11E-10 |
| AKR1C3 | -1.426982657 | 4.677849082 | -7.21184206 | 1.54E-11 | 7.28E-10 |
| SLC2A10 | -1.062148436 | 3.718929416 | -7.188258164 | 1.76E-11 | 8.11E-10 |
| PCK1 | -1.475504303 | 2.760272338 | -7.171284609 | 1.94E-11 | 8.83E-10 |
| TMEM47 | -1.095040723 | 5.043745766 | -7.170583658 | 1.94E-11 | 8.85E-10 |
| SPOCK2 | 1.057674206 | 6.376214336 | 7.163844744 | 2.02E-11 | 9.13E-10 |
| SYNM | -1.223555572 | 5.660397548 | -7.161471189 | 2.05E-11 | 9.22E-10 |
| SLAMF8 | 1.501661586 | 5.432453358 | 7.136917355 | 2.35E-11 | 1.04E-09 |
| SH2D2A | 1.02217402 | 4.290105568 | 7.126504762 | 2.49E-11 | 1.09E-09 |
| CXCR4 | 1.975273786 | 7.0885297 | 7.117572984 | 2.62E-11 | 1.14E-09 |
| OSR2 | -1.16102895 | 6.19367835 | -7.113665127 | 2.68E-11 | 1.16E-09 |
| NTN4 | -1.127100992 | 4.982780396 | -7.080033241 | 3.24E-11 | 1.37E-09 |
| APOC1 | 1.21309651 | 6.387892148 | 7.076888294 | 3.30E-11 | 1.39E-09 |
| PLAT | -1.421291584 | 4.790006992 | -7.026460628 | 4.38E-11 | 1.79E-09 |
| OLFM2 | -1.141597923 | 4.3231814 | -7.020144185 | 4.54E-11 | 1.85E-09 |
| BOK | -1.194484161 | 5.216390864 | -7.016088349 | 4.64E-11 | 1.88E-09 |
| ADAMTS5 | -1.013975928 | 3.0334763 | -7.014385822 | 4.69E-11 | 1.90E-09 |
| SLAMF7 | 1.596148424 | 6.514155877 | 6.975891792 | 5.81E-11 | 2.29E-09 |
| ZFPM2 | -1.016389112 | 2.986151041 | -6.973933453 | 5.87E-11 | 2.30E-09 |
| LPL | -1.925281129 | 5.328549029 | -6.951966106 | 6.64E-11 | 2.59E-09 |
| CD52 | 1.595727299 | 7.748757371 | 6.951044468 | 6.67E-11 | 2.59E-09 |
| CD3G | 1.478065164 | 5.395383945 | 6.944285197 | 6.93E-11 | 2.67E-09 |
| OGN | -1.595707784 | 4.206741741 | -6.937697978 | 7.19E-11 | 2.75E-09 |
| RAC2 | 1.129469682 | 7.247281587 | 6.935011038 | 7.30E-11 | 2.78E-09 |
| PIM2 | 1.004702341 | 6.3433007 | 6.925928745 | 7.67E-11 | 2.91E-09 |
| IGKC | 1.149788899 | 6.840816581 | 6.876534517 | 1.01E-10 | 3.74E-09 |
| TCEAL8 | -1.081951075 | 4.592707896 | -6.864460496 | 1.08E-10 | 3.97E-09 |
| CAPG | 1.179274116 | 7.163397946 | 6.846296465 | 1.19E-10 | 4.33E-09 |
| ARHGAP9 | 1.030845182 | 6.105324891 | 6.845978484 | 1.20E-10 | 4.33E-09 |
| EPDR1 | -1.24642265 | 3.867164416 | -6.838662065 | 1.24E-10 | 4.49E-09 |
| ZBTB16 | -1.305367643 | 5.409064229 | -6.83702477 | 1.26E-10 | 4.53E-09 |
| NLRC5 | 1.016800132 | 5.898174776 | 6.807648334 | 1.48E-10 | 5.22E-09 |
| OSR1 | -1.000504653 | 5.636313275 | -6.804187172 | 1.51E-10 | 5.31E-09 |
| RHOBTB3 | -1.117969406 | 5.210508454 | -6.787559726 | 1.65E-10 | 5.77E-09 |
| IGLJ3 | 1.02644592 | 6.81973377 | 6.778231179 | 1.74E-10 | 6.04E-09 |
| P2RX5 | 1.060760276 | 5.12710244 | 6.75317515 | 1.99E-10 | 6.82E-09 |
| CD3E | 1.561531912 | 5.541127794 | 6.742006543 | 2.12E-10 | 7.19E-09 |
| CCR7 | 1.338763659 | 5.055349796 | 6.741152332 | 2.13E-10 | 7.21E-09 |
| SCP2 | -1.077088785 | 5.793161385 | -6.740570441 | 2.13E-10 | 7.22E-09 |
| LYRM5 | -1.13419933 | 4.539770399 | -6.733795972 | 2.22E-10 | 7.45E-09 |
| BCL11B | 1.0951051 | 4.572459544 | 6.733694669 | 2.22E-10 | 7.45E-09 |
| HIST1H3B | 1.650285849 | 6.23072074 | 6.705357548 | 2.59E-10 | 8.48E-09 |
| HLA-DRA | 1.490874383 | 10.26462261 | 6.651994987 | 3.46E-10 | 1.10E-08 |
| IGLC1 | 1.153810093 | 8.250421542 | 6.61978314 | 4.12E-10 | 1.28E-08 |
| GPX3 | -1.05125436 | 8.821861482 | -6.599824899 | 4.59E-10 | 1.41E-08 |
| AIM2 | 1.505274751 | 5.359298996 | 6.575542935 | 5.24E-10 | 1.59E-08 |
| LRRC15 | 1.239644866 | 4.45867228 | 6.567156037 | 5.48E-10 | 1.66E-08 |
| CFD | -1.481985765 | 7.323610326 | -6.543136337 | 6.24E-10 | 1.86E-08 |
| ANG | -1.238370577 | 6.004427997 | -6.519212903 | 7.10E-10 | 2.07E-08 |
| LGALS2 | 1.002819521 | 6.071646918 | 6.477349645 | 8.89E-10 | 2.53E-08 |
| IKZF3 | 1.260458112 | 5.386650118 | 6.469288868 | 9.28E-10 | 2.61E-08 |
| HLA-DMB | 1.096883331 | 7.822380059 | 6.44710624 | 1.04E-09 | 2.89E-08 |
| PLIN4 | -1.476723008 | 5.929041061 | -6.438505304 | 1.09E-09 | 3.01E-08 |
| IGFBP5 | -1.012988145 | 8.32584525 | -6.413168754 | 1.25E-09 | 3.36E-08 |
| PFN2 | -1.305808632 | 7.191459458 | -6.408058139 | 1.29E-09 | 3.42E-08 |
| LY86 | 1.046716811 | 5.00331236 | 6.40602675 | 1.30E-09 | 3.44E-08 |
| ADAMDEC1 | 1.843955589 | 3.328738653 | 6.378087762 | 1.51E-09 | 3.93E-08 |
| SGCE | -1.002458275 | 6.624569504 | -6.373257622 | 1.55E-09 | 4.02E-08 |
| C6 | -1.075281143 | 4.109097165 | -6.351233594 | 1.74E-09 | 4.46E-08 |
| RAMP2 | -1.013776794 | 6.509320046 | -6.316218865 | 2.09E-09 | 5.17E-08 |
| FAIM3 | 1.259183341 | 6.207656375 | 6.310017643 | 2.16E-09 | 5.31E-08 |
| TAP1 | 1.035927974 | 7.103372959 | 6.302633084 | 2.25E-09 | 5.49E-08 |
| HLA-DPB1 | 1.088401152 | 6.262672368 | 6.265027357 | 2.74E-09 | 6.54E-08 |
| LAPTM5 | 1.395936968 | 8.426366691 | 6.255967641 | 2.87E-09 | 6.83E-08 |
| HIST1H3F | 1.46423071 | 4.70721114 | 6.224954467 | 3.38E-09 | 7.86E-08 |
| MXI1 | -1.011232551 | 5.819021654 | -6.2094364 | 3.67E-09 | 8.39E-08 |
| CD53 | 1.005734718 | 5.352609466 | 6.207567156 | 3.70E-09 | 8.45E-08 |
| PRKAR2B | -1.277940389 | 3.100586588 | -6.193114434 | 3.99E-09 | 8.97E-08 |
| YWHAG | -1.001278301 | 6.190991046 | -6.178280862 | 4.32E-09 | 9.61E-08 |
| GPR174 | 1.317450106 | 4.278934985 | 6.171956195 | 4.46E-09 | 9.90E-08 |
| CD19 | 1.115698073 | 5.32371994 | 6.158886774 | 4.77E-09 | 1.05E-07 |
| CXCL13 | 2.100461667 | 4.55167995 | 6.139910294 | 5.27E-09 | 1.13E-07 |
| G0S2 | -1.709121439 | 6.59574315 | -6.083459492 | 7.06E-09 | 1.46E-07 |
| KLHL6 | 1.06375426 | 4.479001529 | 6.077159222 | 7.29E-09 | 1.50E-07 |
| AC079767.4 | 1.032956019 | 3.602282621 | 6.071573416 | 7.50E-09 | 1.55E-07 |
| CXCL9 | 2.182766208 | 6.053932717 | 6.049156624 | 8.42E-09 | 1.70E-07 |
| SLAMF6 | 1.00606887 | 5.37489056 | 6.045041689 | 8.60E-09 | 1.73E-07 |
| ITM2A | -1.087307223 | 6.006359724 | -6.038047074 | 8.92E-09 | 1.78E-07 |
| SERINC1 | -1.030373963 | 5.089049494 | -5.961587453 | 1.32E-08 | 2.52E-07 |
| NLRC3 | 1.192798109 | 4.711440758 | 5.93934475 | 1.48E-08 | 2.78E-07 |
| CCNG1 | -1.037486926 | 5.992720497 | -5.926777815 | 1.57E-08 | 2.93E-07 |
| GZMK | 1.117171872 | 6.167266707 | 5.90482329 | 1.76E-08 | 3.24E-07 |
| UAP1 | -1.07203366 | 5.597160481 | -5.863476701 | 2.17E-08 | 3.90E-07 |
| CD2 | 1.197652592 | 4.33768524 | 5.862259812 | 2.18E-08 | 3.92E-07 |
| CD22 | 1.085579562 | 5.465080104 | 5.852446389 | 2.29E-08 | 4.10E-07 |
| FCGR1B | 1.510880221 | 4.936964755 | 5.828906725 | 2.58E-08 | 4.54E-07 |
| TOB1 | -1.061302278 | 3.755601796 | -5.825942223 | 2.62E-08 | 4.59E-07 |
| SASH3 | 1.075270983 | 5.819402899 | 5.782346731 | 3.26E-08 | 5.59E-07 |
| IGLV1-44 | 1.161620861 | 7.60313081 | 5.761726551 | 3.61E-08 | 6.13E-07 |
| CCL4 | 1.094390248 | 5.643877075 | 5.747569268 | 3.88E-08 | 6.54E-07 |
| GBP5 | 1.131562932 | 5.107788168 | 5.735730064 | 4.11E-08 | 6.89E-07 |
| BCL2A1 | 1.307378359 | 3.996057213 | 5.632881725 | 6.84E-08 | 1.09E-06 |
| PTPRC | 1.107499382 | 4.84825036 | 5.631931166 | 6.87E-08 | 1.10E-06 |
| SMIM3 | -1.002676384 | 4.890902125 | -5.631884628 | 6.88E-08 | 1.10E-06 |
| CXCL10 | 1.737636753 | 5.835419282 | 5.511953194 | 1.24E-07 | 1.83E-06 |
| KCNA3 | 1.114739091 | 5.112487954 | 5.499728776 | 1.31E-07 | 1.92E-06 |
| PLAC8 | 1.182853097 | 5.277176525 | 5.447246744 | 1.69E-07 | 2.39E-06 |
| CPA3 | -1.245141593 | 4.39787271 | -5.43013705 | 1.83E-07 | 2.57E-06 |
| SPP1 | 1.37301198 | 3.822137766 | 5.429056322 | 1.84E-07 | 2.58E-06 |
| MS4A1 | 1.663717969 | 4.667887221 | 5.415956785 | 1.96E-07 | 2.72E-06 |
| CLMP | -1.029124833 | 5.444183565 | -5.400631519 | 2.11E-07 | 2.89E-06 |
| IGLL5 | 1.322481113 | 8.465721166 | 5.394985517 | 2.17E-07 | 2.95E-06 |
| CHIT1 | 1.30940756 | 5.146673618 | 5.378751611 | 2.34E-07 | 3.15E-06 |
| HIST1H3C | 1.019628143 | 5.73248254 | 5.323446474 | 3.05E-07 | 3.94E-06 |
| HBB | -1.080979986 | 6.406081225 | -5.321388823 | 3.08E-07 | 3.98E-06 |
| SELL | 1.437493857 | 5.710514646 | 5.273483799 | 3.86E-07 | 4.80E-06 |
| RASGRP1 | 1.017947496 | 3.969639249 | 5.256653112 | 4.18E-07 | 5.16E-06 |
| MIR100HG | -1.242104796 | 4.886247778 | -5.175602739 | 6.11E-07 | 7.18E-06 |
| SFRP1 | -1.230196944 | 7.018231023 | -5.127128204 | 7.65E-07 | 8.71E-06 |
| ANKRD22 | 1.051363787 | 3.983796073 | 5.119110825 | 7.94E-07 | 8.97E-06 |
| C15orf48 | 1.281464983 | 3.22296987 | 5.075988112 | 9.68E-07 | 1.06E-05 |
| TCL1A | 1.257197154 | 5.399322988 | 5.010341921 | 1.31E-06 | 1.38E-05 |
| FGR | 1.08695019 | 5.498696616 | 4.969528169 | 1.57E-06 | 1.63E-05 |
| PLA2G7 | 1.12575966 | 5.037218284 | 4.957730743 | 1.66E-06 | 1.70E-05 |
| LCP1 | 1.025456824 | 6.403611786 | 4.923869065 | 1.93E-06 | 1.96E-05 |
| MYO5C | -1.043270702 | 5.043093603 | -4.91515382 | 2.01E-06 | 2.02E-05 |
| TCEAL2 | -1.189281706 | 5.049764006 | -4.851217179 | 2.67E-06 | 2.60E-05 |
| HLA-DPA1 | 1.008384359 | 7.742546736 | 4.737146008 | 4.42E-06 | 4.03E-05 |
| IDO1 | 1.219714862 | 4.418865866 | 4.621533421 | 7.31E-06 | 6.19E-05 |
| VCAN | 1.090763313 | 5.834654181 | 4.613687722 | 7.56E-06 | 6.36E-05 |
| IRX3 | -1.027393767 | 5.052762569 | -4.447355332 | 1.53E-05 | 0.00011679 |
| MMP7 | 1.092485954 | 5.343884158 | 4.420635906 | 1.71E-05 | 0.000128681 |
| MMP10 | 1.053870613 | 3.573200734 | 4.298668576 | 2.83E-05 | 0.000199138 |
| CYTIP | 1.060044386 | 5.519318204 | 4.275401124 | 3.11E-05 | 0.000215791 |
| FDCSP | 1.956398989 | 6.414984501 | 4.125723389 | 5.67E-05 | 0.000364805 |
| IGHD | 1.056196393 | 7.183395927 | 4.016060013 | 8.73E-05 | 0.000530533 |
| FNDC1 | 1.0681358 | 4.100637785 | 3.965248027 | 0.000106221 | 0.000628104 |
| CCL18 | 1.247785171 | 6.113480614 | 3.851379283 | 0.000163884 | 0.000904832 |
| OLFM4 | 1.125572769 | 3.714594958 | 3.412097176 | 0.000798527 | 0.003445472 |
| LYZ | 1.424552745 | 10.4266248 | 3.394247398 | 0.000849001 | 0.003622517 |
| HLA-DQA1 | 1.526563848 | 7.029904979 | 3.196540199 | 0.001646922 | 0.006291893 |
| IGJ | 1.298367998 | 7.610171178 | 3.091887458 | 0.002310462 | 0.00830715 |
| LTF | 1.826715569 | 10.28926435 | 3.07840849 | 0.00241194 | 0.008612821 |
| SCGB2A2 | -1.013207333 | 3.893518563 | -2.608050163 | 0.009882211 | 0.027107854 |

# Appendix 3

**LASSO and** **SVM-RFE genes**

**Table S3a. LASSO genes.**

| HLF | IGSF10 | F8 | PALMD | C6 | GPR34 |
| --- | --- | --- | --- | --- | --- |
| PGM1 | PLSCR4 | IRF8 | SMIM19 | RHOBTB3 | FBP1 |
| GPR146 | ENPP6 | MAP1B | TNS1 | CCL4 | SMR3B |
| GSTM3 | NECAB1 | IGLL3P | CD3E | IGK | CPA3 |
| TCIRG1 | ATXN1L | PLA2G16 | LRRC15 | CD79A | HIST1H2BM |
| TIMP4 | AKR1C1 | SPOCK2 | GBE1 | TCEAL8 | IDO1 |
| CORO2B | MARC1 | APOC1 | MGLL | SUCLA2 | FGR |
| RUNX1-IT1 | COX7A1 | RBPMS2 | TMEM47 | ALDH2 | IGHD |
| LYVE1 | TMEM200B | UGDH | ADAMDEC1 | RCAN2 | FDCSP |
| PARVG | CALB2 | ZBTB16 | HLA-DPB1 | SPP1 | OLFM4 |
| CCL18 |  |  |  |  |  |

**Table S3b. SVM-RFE genes.**

| IGK | TUSC1 | OLFML1 | MIR100HG | MAP1B | IGJ |
| --- | --- | --- | --- | --- | --- |
| IRF8 | AKR1C1 | GPR146 | SASH3 | TNMD | DENND1C |
| HLF | RHOBTB3 | PALMD | CCL4 | CORO2B | ENPP6 |
| PGM1 | TNS1 | PLA2G16 | IGSF10 |  |  |

**Table S3c. InterGenes.**

| HLF | IGK | CCL4 | TNS1 | MAP1B |
| --- | --- | --- | --- | --- |
| PGM1 | CORO2B | PLA2G16 | RHOBTB3 | AKR1C1 |
| GPR146 | IGSF10 | PALMD | ENPP6 | IRF8 |

# Appendix 4

## **DEG Identification of IRF8**

**Table S4. 507 DEG Identification of IRF8**

| id | logFC | AveExpr | t | P.Value | adj.P.Val |
| --- | --- | --- | --- | --- | --- |
| IRF8 | 1.558101259 | 6.405807672 | 16.65335834 | 1.70E-34 | 3.69E-30 |
| ARHGAP25 | 1.009233394 | 5.058015022 | 14.07868207 | 3.09E-28 | 3.35E-24 |
| CD84 | 1.396577691 | 5.130320199 | 13.92397527 | 7.50E-28 | 5.42E-24 |
| FMNL3 | 1.154142495 | 4.990037251 | 13.57134171 | 5.69E-27 | 2.08E-23 |
| CD53 | 1.509739886 | 5.599732854 | 13.54730978 | 6.54E-27 | 2.08E-23 |
| CYBB | 1.438200146 | 5.826484095 | 13.42901623 | 1.29E-26 | 3.50E-23 |
| CIITA | 1.697437908 | 6.181944645 | 13.33308328 | 2.25E-26 | 5.41E-23 |
| IL21R | 1.461505314 | 5.305413184 | 13.20042252 | 4.84E-26 | 8.78E-23 |
| ITGAL | 1.473389407 | 6.285330619 | 13.19973553 | 4.86E-26 | 8.78E-23 |
| CHST11 | 1.554556711 | 5.592782634 | 13.08488242 | 9.45E-26 | 1.57E-22 |
| PLEK | 1.626130486 | 6.787503446 | 13.05222728 | 1.14E-25 | 1.77E-22 |
| RAC2 | 1.453436629 | 7.524808423 | 12.86240632 | 3.43E-25 | 4.68E-22 |
| LCK | 1.398135091 | 5.429645374 | 12.82864956 | 4.18E-25 | 5.32E-22 |
| LAPTM5 | 2.065202901 | 8.769368346 | 12.7771671 | 5.63E-25 | 6.53E-22 |
| FGD3 | 1.139832918 | 6.651241435 | 12.77429333 | 5.73E-25 | 6.53E-22 |
| CD48 | 1.07188496 | 5.348933295 | 12.51326861 | 2.61E-24 | 2.46E-21 |
| HLA-DMB | 1.517696275 | 8.091899964 | 12.49671782 | 2.88E-24 | 2.60E-21 |
| BCL2A1 | 2.136891671 | 4.317298752 | 12.47954392 | 3.18E-24 | 2.75E-21 |
| GMFG | 1.153317839 | 7.186503674 | 12.39706136 | 5.14E-24 | 4.12E-21 |
| LCP1 | 1.871893126 | 6.655581177 | 12.36096671 | 6.34E-24 | 4.78E-21 |
| COTL1 | 1.012161166 | 5.906981609 | 12.35930672 | 6.40E-24 | 4.78E-21 |
| TRBC1 | 1.967926201 | 7.029149906 | 12.32492281 | 7.82E-24 | 5.65E-21 |
| ARHGAP30 | 1.02006067 | 7.039507237 | 12.310969 | 8.48E-24 | 5.93E-21 |
| LCP2 | 1.283009395 | 4.412595315 | 12.29337983 | 9.40E-24 | 6.36E-21 |
| TRAC | 1.372867469 | 7.620994486 | 12.26469032 | 1.11E-23 | 6.99E-21 |
| PIK3CD | 1.074401819 | 6.193108364 | 12.26186486 | 1.13E-23 | 6.99E-21 |
| JAK3 | 1.042355094 | 5.762317484 | 12.2069196 | 1.56E-23 | 9.36E-21 |
| PARVG | 1.133382663 | 5.785706996 | 12.17401162 | 1.89E-23 | 1.05E-20 |
| PLA2G2D | 2.470255121 | 6.438345745 | 12.17292493 | 1.90E-23 | 1.05E-20 |
| FYB | 1.377217815 | 5.855384508 | 12.14790667 | 2.20E-23 | 1.19E-20 |
| RHOH | 1.442247204 | 4.947601839 | 12.10529001 | 2.81E-23 | 1.49E-20 |
| FERMT3 | 1.147663531 | 6.832864176 | 12.08422639 | 3.18E-23 | 1.60E-20 |
| AIM2 | 1.967749199 | 5.729166506 | 12.08419301 | 3.18E-23 | 1.60E-20 |
| ARHGDIB | 1.081951113 | 7.425091001 | 12.07580149 | 3.34E-23 | 1.65E-20 |
| IL2RG | 1.924160084 | 8.248275528 | 11.98692743 | 5.61E-23 | 2.70E-20 |
| PTPRC | 1.723450399 | 5.12037878 | 11.8315135 | 1.39E-22 | 6.41E-20 |
| SPOCK2 | 1.238055159 | 6.636099998 | 11.81068466 | 1.57E-22 | 7.09E-20 |
| CXCL13 | 3.047162852 | 5.067793388 | 11.7797483 | 1.88E-22 | 8.32E-20 |
| IL32 | 1.450626145 | 8.063609545 | 11.74297428 | 2.33E-22 | 9.91E-20 |
| CORO1A | 1.631330822 | 8.132918758 | 11.70426685 | 2.92E-22 | 1.19E-19 |
| FAM107A | -1.120916107 | 5.884350378 | -11.66905658 | 3.59E-22 | 1.44E-19 |
| PPP1R18 | 1.33378172 | 7.124887151 | 11.64069876 | 4.24E-22 | 1.64E-19 |
| DOK3 | 1.057114272 | 5.763244799 | 11.60454434 | 5.24E-22 | 1.99E-19 |
| MMP9 | 2.259135385 | 7.340408448 | 11.51011121 | 9.09E-22 | 3.28E-19 |
| SASH3 | 1.561492602 | 6.083612341 | 11.48165495 | 1.07E-21 | 3.81E-19 |
| LAIR1 | 1.3913503 | 7.055604735 | 11.47788678 | 1.10E-21 | 3.83E-19 |
| ARHGAP9 | 1.281460139 | 6.358618279 | 11.42978615 | 1.45E-21 | 4.90E-19 |
| ITGB2 | 1.36983706 | 7.179905839 | 11.42343348 | 1.51E-21 | 4.95E-19 |
| GM2A | 1.152517661 | 6.236051116 | 11.40896971 | 1.64E-21 | 5.31E-19 |
| IL2RB | 1.349159822 | 5.508774404 | 11.321447 | 2.74E-21 | 8.47E-19 |
| TAGAP | 1.067682129 | 3.794202436 | 11.28595609 | 3.37E-21 | 1.01E-18 |
| CD3G | 1.733551436 | 5.758565671 | 11.27708927 | 3.55E-21 | 1.05E-18 |
| LY86 | 1.406290553 | 5.260505633 | 11.27214218 | 3.65E-21 | 1.07E-18 |
| CD3E | 1.910685271 | 5.924818492 | 11.24455122 | 4.29E-21 | 1.22E-18 |
| IL7R | 1.867426324 | 6.035311774 | 11.21327615 | 5.15E-21 | 1.41E-18 |
| CCL19 | 1.91980282 | 8.327317726 | 11.20399799 | 5.44E-21 | 1.45E-18 |
| IKZF3 | 1.556784137 | 5.696362683 | 11.19565027 | 5.71E-21 | 1.51E-18 |
| ADCY7 | 1.090381556 | 5.166891409 | 11.14667898 | 7.60E-21 | 1.98E-18 |
| FGR | 1.910758538 | 5.765775806 | 11.13219811 | 8.27E-21 | 2.11E-18 |
| BCL11B | 1.330561428 | 4.841542511 | 11.05364979 | 1.31E-20 | 3.22E-18 |
| PDCD1LG2 | 1.519542801 | 5.033181421 | 11.03978145 | 1.42E-20 | 3.41E-18 |
| CD3D | 1.3775331 | 6.597445345 | 11.02745374 | 1.52E-20 | 3.55E-18 |
| ALOX5AP | 1.330387417 | 6.160736208 | 11.00038564 | 1.79E-20 | 4.11E-18 |
| CD28 | 1.088776758 | 3.986415611 | 10.99628595 | 1.83E-20 | 4.17E-18 |
| LEF1 | 1.017854323 | 5.154292689 | 10.96075205 | 2.25E-20 | 5.02E-18 |
| HCLS1 | 1.798442887 | 7.040751333 | 10.94260269 | 2.50E-20 | 5.47E-18 |
| CD52 | 1.861019173 | 8.140850365 | 10.9277721 | 2.73E-20 | 5.90E-18 |
| HLA-DRA | 1.904019737 | 10.63095174 | 10.9262872 | 2.75E-20 | 5.90E-18 |
| LST1 | 1.169639026 | 5.945362993 | 10.91382368 | 2.96E-20 | 6.28E-18 |
| CXCR4 | 2.200488245 | 7.573882687 | 10.86581738 | 3.92E-20 | 8.23E-18 |
| TRAF1 | 1.300740855 | 5.300843395 | 10.86106052 | 4.03E-20 | 8.38E-18 |
| P2RY10 | 1.071200185 | 4.00102543 | 10.85029288 | 4.29E-20 | 8.78E-18 |
| RCSD1 | 1.217352611 | 5.319767891 | 10.84999327 | 4.30E-20 | 8.78E-18 |
| IL10RA | 1.405650353 | 6.140269293 | 10.83573446 | 4.67E-20 | 9.30E-18 |
| SIGLEC10 | 1.4021936 | 5.836623969 | 10.83518088 | 4.68E-20 | 9.30E-18 |
| TAP2 | 1.111631494 | 5.212107738 | 10.80389905 | 5.62E-20 | 1.11E-17 |
| HLA-DPA1 | 1.819902488 | 7.990321178 | 10.79938885 | 5.77E-20 | 1.13E-17 |
| SELL | 2.234961368 | 6.063727422 | 10.79559534 | 5.90E-20 | 1.14E-17 |
| PTPN6 | 1.078016489 | 8.453600587 | 10.77252162 | 6.75E-20 | 1.27E-17 |
| ANKRD44 | 1.009852017 | 4.528153571 | 10.7618766 | 7.18E-20 | 1.34E-17 |
| ADAMDEC1 | 2.525988637 | 3.781824883 | 10.7523519 | 7.59E-20 | 1.39E-17 |
| DENND1C | 1.33851927 | 6.404746425 | 10.74468182 | 7.94E-20 | 1.44E-17 |
| GPR174 | 1.696505682 | 4.602651297 | 10.72212731 | 9.06E-20 | 1.63E-17 |
| LYN | 1.185767072 | 5.827005545 | 10.7198554 | 9.18E-20 | 1.64E-17 |
| CD72 | 1.441201847 | 4.59567565 | 10.70914523 | 9.77E-20 | 1.73E-17 |
| PIK3CG | 1.076935378 | 3.977903708 | 10.7084785 | 9.81E-20 | 1.73E-17 |
| BIN2 | 1.240701637 | 6.046795911 | 10.70116806 | 1.02E-19 | 1.79E-17 |
| CD86 | 1.128187643 | 4.934888579 | 10.69827953 | 1.04E-19 | 1.80E-17 |
| TBC1D10C | 1.379960416 | 6.939724303 | 10.62924356 | 1.56E-19 | 2.63E-17 |
| PPP1R1A | -1.776591626 | 5.189974981 | -10.58114276 | 2.06E-19 | 3.43E-17 |
| GZMK | 1.506365615 | 6.441771795 | 10.56812453 | 2.22E-19 | 3.67E-17 |
| ADAM28 | 1.106302343 | 5.054917869 | 10.4996528 | 3.31E-19 | 5.31E-17 |
| ITGB2-AS1 | 1.119152043 | 6.206039389 | 10.49348195 | 3.43E-19 | 5.46E-17 |
| TAP1 | 1.345847816 | 7.357915262 | 10.48154842 | 3.68E-19 | 5.73E-17 |
| MTURN | -1.086647539 | 5.078261273 | -10.47321864 | 3.86E-19 | 5.97E-17 |
| IL4R | 1.213890135 | 7.43372304 | 10.47151775 | 3.90E-19 | 5.99E-17 |
| IL2RA | 1.129439464 | 4.713436358 | 10.4565554 | 4.25E-19 | 6.44E-17 |
| DOCK8 | 1.317386839 | 4.494624731 | 10.43097729 | 4.94E-19 | 7.37E-17 |
| 1-Mar | 1.038827648 | 3.739052741 | 10.42935687 | 4.98E-19 | 7.39E-17 |
| LILRB1 | 1.273536936 | 6.387525064 | 10.40569256 | 5.72E-19 | 8.43E-17 |
| TMEM163 | 1.270907519 | 2.908115944 | 10.37678827 | 6.77E-19 | 9.90E-17 |
| SLC20A1 | 1.23642929 | 4.29245926 | 10.36305358 | 7.33E-19 | 1.07E-16 |
| HIST1H2BM | 1.483144828 | 5.693603463 | 10.33989166 | 8.39E-19 | 1.19E-16 |
| SP110 | 1.08225638 | 6.225099241 | 10.33545228 | 8.61E-19 | 1.22E-16 |
| PARP15 | 1.517715933 | 4.186966625 | 10.32596027 | 9.09E-19 | 1.28E-16 |
| CCL4 | 1.534932214 | 5.912784393 | 10.31017849 | 9.97E-19 | 1.38E-16 |
| CD247 | 1.149192559 | 4.919993351 | 10.30492847 | 1.03E-18 | 1.42E-16 |
| CCR7 | 1.569960149 | 5.384303153 | 10.30106721 | 1.05E-18 | 1.44E-16 |
| ICAM1 | 1.189054844 | 6.271803648 | 10.27287695 | 1.24E-18 | 1.66E-16 |
| TRAF3IP3 | 1.204386843 | 4.373119652 | 10.27210348 | 1.24E-18 | 1.66E-16 |
| ELF4 | 1.004377438 | 5.331559908 | 10.2559209 | 1.37E-18 | 1.80E-16 |
| CD226 | 1.098497218 | 4.634366165 | 10.25508461 | 1.37E-18 | 1.80E-16 |
| SAMD9L | 1.377051257 | 5.563863967 | 10.25189244 | 1.40E-18 | 1.82E-16 |
| ADIRF | -1.605428128 | 7.697660376 | -10.23271218 | 1.56E-18 | 2.00E-16 |
| WIPF1 | 1.100474775 | 5.972348434 | 10.22085251 | 1.67E-18 | 2.12E-16 |
| FPR3 | 1.539396384 | 6.27344379 | 10.21923672 | 1.69E-18 | 2.13E-16 |
| SLAMF8 | 1.738389872 | 5.801433062 | 10.20442274 | 1.84E-18 | 2.31E-16 |
| PLEKHO1 | 1.041086884 | 7.288833235 | 10.19273372 | 1.97E-18 | 2.45E-16 |
| SRGN | 1.172218312 | 6.064895984 | 10.19124123 | 1.99E-18 | 2.46E-16 |
| CD2 | 1.624789337 | 4.631965591 | 10.18016984 | 2.12E-18 | 2.60E-16 |
| KMO | 1.262524897 | 3.792335734 | 10.11350546 | 3.12E-18 | 3.72E-16 |
| C1orf162 | 1.600446143 | 6.348705947 | 10.10886631 | 3.21E-18 | 3.78E-16 |
| HIST1H3B | 2.005249935 | 6.636219548 | 10.10792632 | 3.23E-18 | 3.78E-16 |
| SERPINB9 | 1.068865133 | 4.882566401 | 10.09974709 | 3.38E-18 | 3.94E-16 |
| CYFIP2 | 1.152101024 | 5.18574193 | 10.09823173 | 3.41E-18 | 3.95E-16 |
| MPEG1 | 1.374368062 | 6.321799495 | 10.09212434 | 3.54E-18 | 4.05E-16 |
| NLRC5 | 1.180047166 | 6.148017095 | 10.06784774 | 4.07E-18 | 4.64E-16 |
| SH2D2A | 1.168731109 | 4.541268327 | 10.0241237 | 5.24E-18 | 5.85E-16 |
| CXCL9 | 2.861760304 | 6.590269557 | 10.01473125 | 5.54E-18 | 6.13E-16 |
| HIST1H3F | 1.916310967 | 5.066993543 | 9.993073779 | 6.28E-18 | 6.90E-16 |
| ACTG2 | -1.021914495 | 4.332013751 | -9.984228213 | 6.61E-18 | 7.19E-16 |
| SLAMF6 | 1.298074388 | 5.622096054 | 9.972051418 | 7.09E-18 | 7.61E-16 |
| THEMIS2 | 1.141176505 | 6.433049888 | 9.956627433 | 7.75E-18 | 8.27E-16 |
| BTK | 1.05052741 | 6.587768264 | 9.948452696 | 8.13E-18 | 8.58E-16 |
| CXorf65 | 1.022825345 | 4.080532032 | 9.935360102 | 8.77E-18 | 9.22E-16 |
| CSF2RB | 1.458791589 | 4.691822341 | 9.931020293 | 8.99E-18 | 9.40E-16 |
| GPR65 | 1.242570939 | 3.664013973 | 9.891343509 | 1.13E-17 | 1.16E-15 |
| PAG1 | 1.218578221 | 4.187675609 | 9.871533688 | 1.27E-17 | 1.27E-15 |
| HVCN1 | 1.007775341 | 5.733402482 | 9.861268674 | 1.35E-17 | 1.33E-15 |
| PARP14 | 1.121569886 | 5.662918679 | 9.854510861 | 1.40E-17 | 1.37E-15 |
| MYH11 | -1.174601944 | 5.854541744 | -9.842774788 | 1.50E-17 | 1.45E-15 |
| ANO1 | -1.581623919 | 6.059711982 | -9.806510267 | 1.85E-17 | 1.77E-15 |
| RAB3D | -1.091748978 | 6.413573233 | -9.799414816 | 1.92E-17 | 1.83E-15 |
| PTPRCAP | 1.064234684 | 7.516235147 | 9.798353948 | 1.94E-17 | 1.83E-15 |
| BMP2K | 1.015195004 | 4.273815563 | 9.797425747 | 1.95E-17 | 1.83E-15 |
| TNFSF13B | 1.406024671 | 4.863735082 | 9.782813891 | 2.12E-17 | 1.99E-15 |
| HCK | 1.500139457 | 5.730166405 | 9.779260207 | 2.16E-17 | 2.02E-15 |
| HIST1H3C | 1.558083226 | 5.983019741 | 9.772804343 | 2.24E-17 | 2.08E-15 |
| PLEKHA2 | 1.020220399 | 6.079975102 | 9.765689918 | 2.34E-17 | 2.15E-15 |
| MAOA | -1.770359396 | 5.008358168 | -9.750575976 | 2.55E-17 | 2.34E-15 |
| HIST1H3I | 1.152190301 | 6.527689079 | 9.717709911 | 3.08E-17 | 2.81E-15 |
| GBP5 | 1.562087022 | 5.385829345 | 9.689192743 | 3.63E-17 | 3.28E-15 |
| TRG-AS1 | 1.280503489 | 4.165102777 | 9.666076769 | 4.15E-17 | 3.70E-15 |
| MGLL | -1.234235305 | 7.169740717 | -9.66460781 | 4.19E-17 | 3.72E-15 |
| STK17A | 1.195108524 | 4.035606664 | 9.647426683 | 4.62E-17 | 4.09E-15 |
| FCER1G | 1.965802386 | 7.571183795 | 9.645432219 | 4.68E-17 | 4.12E-15 |
| ITK | 1.275603527 | 4.816308153 | 9.626366557 | 5.22E-17 | 4.56E-15 |
| HLA-DMA | 1.496474593 | 8.077349037 | 9.612768435 | 5.65E-17 | 4.87E-15 |
| PLAC8 | 1.614238247 | 5.567820429 | 9.587251785 | 6.54E-17 | 5.57E-15 |
| ETS1 | 1.158501398 | 5.882392497 | 9.577484333 | 6.92E-17 | 5.87E-15 |
| CECR1 | 1.484050969 | 7.722428643 | 9.525573739 | 9.32E-17 | 7.83E-15 |
| SNX10 | 2.138735302 | 5.159589764 | 9.513748934 | 9.98E-17 | 8.28E-15 |
| KIF21B | 1.189287218 | 5.097565749 | 9.4664545 | 1.31E-16 | 1.08E-14 |
| FAM26F | 1.048594679 | 4.69300323 | 9.434570505 | 1.57E-16 | 1.26E-14 |
| KIAA0226L | 1.069805634 | 4.160118515 | 9.426713185 | 1.65E-16 | 1.31E-14 |
| AC079767.4 | 1.326224954 | 3.856094671 | 9.415700189 | 1.75E-16 | 1.39E-14 |
| CCR5 | 1.026079989 | 4.843091516 | 9.412070933 | 1.79E-16 | 1.41E-14 |
| AC017002.2 | 1.266406118 | 6.157790443 | 9.401345723 | 1.90E-16 | 1.48E-14 |
| ANKRD44-IT1 | 1.300376399 | 5.285858146 | 9.390732529 | 2.02E-16 | 1.56E-14 |
| PLA2G7 | 1.791616673 | 5.313833515 | 9.340160699 | 2.70E-16 | 2.04E-14 |
| TFEC | 1.10125127 | 2.782512608 | 9.314677233 | 3.13E-16 | 2.34E-14 |
| NLRC3 | 1.49631801 | 5.004528293 | 9.292305368 | 3.55E-16 | 2.63E-14 |
| SERPINA1 | 1.103052025 | 4.522960962 | 9.243223643 | 4.71E-16 | 3.41E-14 |
| CNN1 | -1.183634801 | 6.351463817 | -9.237638985 | 4.86E-16 | 3.48E-14 |
| FAM46B | -1.277930551 | 4.997908702 | -9.227622658 | 5.15E-16 | 3.67E-14 |
| CD96 | 1.233315934 | 3.280556381 | 9.206452548 | 5.81E-16 | 4.08E-14 |
| PVRIG | 1.030266606 | 4.315455539 | 9.202450786 | 5.94E-16 | 4.16E-14 |
| CST7 | 1.108998846 | 5.930821943 | 9.175033412 | 6.95E-16 | 4.79E-14 |
| CNTRL | 1.053014595 | 4.513317014 | 9.163369813 | 7.43E-16 | 5.06E-14 |
| C1orf54 | 1.201754429 | 6.544887728 | 9.163197678 | 7.44E-16 | 5.06E-14 |
| RASGRP1 | 1.421741073 | 4.219763491 | 9.159901153 | 7.58E-16 | 5.14E-14 |
| KLHDC7B | 1.302441191 | 4.912581366 | 9.148790248 | 8.07E-16 | 5.46E-14 |
| PARP12 | 1.038793531 | 6.23638021 | 9.140358245 | 8.47E-16 | 5.70E-14 |
| IRX5 | -1.199480769 | 5.501986267 | -9.127825208 | 9.10E-16 | 6.10E-14 |
| KLHL6 | 1.272365222 | 4.740381147 | 9.111171628 | 1.00E-15 | 6.63E-14 |
| ARHGEF37 | -1.090189641 | 5.945369733 | -9.111042427 | 1.00E-15 | 6.63E-14 |
| JAK2 | 1.118609685 | 4.597504846 | 9.102218241 | 1.05E-15 | 6.95E-14 |
| CTSS | 1.355145929 | 6.206630753 | 9.098156503 | 1.08E-15 | 7.09E-14 |
| PHGDH | -1.27384526 | 6.426025131 | -9.091922238 | 1.12E-15 | 7.28E-14 |
| TLR8 | 1.010263565 | 3.660392436 | 9.08596738 | 1.15E-15 | 7.49E-14 |
| CTSC | 1.136073317 | 5.944427647 | 9.079608672 | 1.20E-15 | 7.72E-14 |
| SEPT6 | 1.060555391 | 6.256457469 | 9.078757461 | 1.20E-15 | 7.73E-14 |
| EPSTI1 | 1.135792437 | 5.902832179 | 9.067012196 | 1.29E-15 | 8.24E-14 |
| DOCK11 | 1.325223629 | 6.063696046 | 9.061937027 | 1.32E-15 | 8.46E-14 |
| STAT1 | 1.631214488 | 6.950376879 | 9.057233454 | 1.36E-15 | 8.66E-14 |
| CLEC2D | 1.165383906 | 3.679749867 | 9.035246569 | 1.54E-15 | 9.73E-14 |
| P2RX5 | 1.13335525 | 5.387746394 | 9.025321877 | 1.63E-15 | 1.02E-13 |
| FXYD5 | 1.016542175 | 8.1480436 | 9.004513189 | 1.84E-15 | 1.12E-13 |
| EVI2B | 1.546234786 | 5.381648423 | 8.977551593 | 2.14E-15 | 1.30E-13 |
| HLA-DPB1 | 1.292723862 | 6.53010808 | 8.970721029 | 2.22E-15 | 1.34E-13 |
| GZMA | 1.216579866 | 5.043128785 | 8.969232147 | 2.24E-15 | 1.35E-13 |
| RNF19B | 1.106988797 | 5.349928911 | 8.964985883 | 2.30E-15 | 1.38E-13 |
| SLC2A3 | 1.042809569 | 4.342713398 | 8.948436062 | 2.52E-15 | 1.51E-13 |
| BTG1 | 1.003879331 | 6.032347287 | 8.940081813 | 2.65E-15 | 1.57E-13 |
| PCED1B | 1.067032784 | 5.046627342 | 8.93174907 | 2.78E-15 | 1.64E-13 |
| P2RY8 | 1.00666909 | 6.029078024 | 8.908181487 | 3.17E-15 | 1.86E-13 |
| STAT4 | 1.110095601 | 5.116904906 | 8.900140277 | 3.32E-15 | 1.93E-13 |
| LRMP | 1.26265454 | 5.66965579 | 8.88804642 | 3.56E-15 | 2.06E-13 |
| CCR1 | 1.187804231 | 5.073806818 | 8.878426953 | 3.76E-15 | 2.16E-13 |
| RNASE6 | 1.335917344 | 6.458799231 | 8.858436236 | 4.21E-15 | 2.37E-13 |
| MYD88 | 1.175815369 | 6.282398061 | 8.842212496 | 4.61E-15 | 2.57E-13 |
| CKS2 | 1.010492538 | 3.143355255 | 8.839198483 | 4.69E-15 | 2.61E-13 |
| GVINP1 | 1.218637459 | 4.176613886 | 8.838141895 | 4.72E-15 | 2.62E-13 |
| PSMB9 | 1.091176724 | 6.685958624 | 8.83652954 | 4.76E-15 | 2.64E-13 |
| UCP2 | 1.067447268 | 8.237061506 | 8.823400821 | 5.13E-15 | 2.83E-13 |
| S100A1 | -1.809218644 | 6.778884438 | -8.819778796 | 5.24E-15 | 2.88E-13 |
| GIMAP4 | 1.346257946 | 5.776626212 | 8.815676669 | 5.36E-15 | 2.92E-13 |
| CTSH | 1.151856478 | 7.928001037 | 8.811782347 | 5.48E-15 | 2.97E-13 |
| NDRG2 | -1.172271738 | 6.466282105 | -8.800074605 | 5.85E-15 | 3.14E-13 |
| OSR1 | -1.092542952 | 5.390474989 | -8.794601931 | 6.04E-15 | 3.22E-13 |
| ITGA4 | 1.230510552 | 4.783887196 | 8.785832319 | 6.34E-15 | 3.37E-13 |
| PEBP4 | -1.291242421 | 4.70641426 | -8.777114686 | 6.66E-15 | 3.52E-13 |
| P2RY13 | 1.250734585 | 4.613881098 | 8.771435762 | 6.88E-15 | 3.63E-13 |
| LAP3 | 1.353690566 | 6.427254562 | 8.770732571 | 6.91E-15 | 3.63E-13 |
| PON3 | -1.803371339 | 4.805658511 | -8.763627102 | 7.19E-15 | 3.74E-13 |
| FAM174B | -1.192483427 | 5.18953164 | -8.763439886 | 7.20E-15 | 3.74E-13 |
| NKG7 | 1.262555863 | 5.806240001 | 8.763052595 | 7.21E-15 | 3.74E-13 |
| HIST1H3G | 1.033854594 | 6.670463898 | 8.723431912 | 9.02E-15 | 4.61E-13 |
| GZMB | 1.515323176 | 3.983156806 | 8.678846583 | 1.16E-14 | 5.84E-13 |
| RAB31 | 1.247131531 | 6.510530504 | 8.660563399 | 1.29E-14 | 6.43E-13 |
| STK17B | 1.001586624 | 5.321895778 | 8.634133698 | 1.49E-14 | 7.33E-13 |
| ENPP2 | 1.704178404 | 5.379913397 | 8.607305595 | 1.74E-14 | 8.37E-13 |
| SELENBP1 | -1.101718825 | 6.51321984 | -8.60516441 | 1.76E-14 | 8.45E-13 |
| TCL1A | 1.822512952 | 5.708234289 | 8.594399405 | 1.87E-14 | 8.96E-13 |
| RASSF2 | 1.31872365 | 5.979864801 | 8.592763085 | 1.88E-14 | 9.00E-13 |
| CPVL | 1.265137654 | 5.499775168 | 8.588990302 | 1.92E-14 | 9.16E-13 |
| FAIM3 | 1.369430062 | 6.51705571 | 8.539698012 | 2.54E-14 | 1.19E-12 |
| TMEM176A | 1.22329235 | 5.652903892 | 8.526863332 | 2.73E-14 | 1.28E-12 |
| PTGDS | 1.500782608 | 8.410410374 | 8.524680435 | 2.76E-14 | 1.29E-12 |
| PLXNB3 | -1.139699279 | 4.349548639 | -8.517505592 | 2.87E-14 | 1.34E-12 |
| TMSB10 | 1.213748608 | 11.50782549 | 8.504733774 | 3.09E-14 | 1.43E-12 |
| BIRC3 | 1.005709367 | 3.606794686 | 8.50387895 | 3.10E-14 | 1.44E-12 |
| ZC3H12D | 1.412713451 | 3.220536266 | 8.488178056 | 3.39E-14 | 1.56E-12 |
| CAPG | 1.287241256 | 7.453162444 | 8.478353078 | 3.58E-14 | 1.64E-12 |
| ATP6V1B2 | 1.084963006 | 5.885973851 | 8.443248906 | 4.36E-14 | 1.97E-12 |
| PTGER4 | 1.159812645 | 4.498062684 | 8.434759702 | 4.57E-14 | 2.06E-12 |
| MYO1F | 1.125106852 | 6.121963867 | 8.430570969 | 4.68E-14 | 2.09E-12 |
| SMAP2 | 1.128357541 | 6.999979684 | 8.42803233 | 4.74E-14 | 2.12E-12 |
| FCGR2B | 1.302507558 | 6.208183739 | 8.426839622 | 4.77E-14 | 2.12E-12 |
| FCGR1B | 1.932922448 | 5.30820961 | 8.425049127 | 4.82E-14 | 2.14E-12 |
| DENND2D | 1.077872445 | 5.07008974 | 8.422384463 | 4.89E-14 | 2.17E-12 |
| PTTG1 | 1.029330138 | 5.417029105 | 8.417723564 | 5.02E-14 | 2.22E-12 |
| PFN1 | 1.038841098 | 9.487014748 | 8.404332655 | 5.41E-14 | 2.37E-12 |
| IDO1 | 1.899562075 | 4.718567232 | 8.385654077 | 6.01E-14 | 2.61E-12 |
| MFI2 | -1.045783857 | 5.12242588 | -8.378115029 | 6.27E-14 | 2.71E-12 |
| CDR1 | -1.653400948 | 5.652504678 | -8.359744804 | 6.94E-14 | 2.98E-12 |
| NUSAP1 | 1.017327092 | 3.884461141 | 8.352494726 | 7.23E-14 | 3.07E-12 |
| CD19 | 1.291233165 | 5.597862895 | 8.337597932 | 7.86E-14 | 3.31E-12 |
| GPR183 | 1.18753082 | 5.121253788 | 8.336239417 | 7.92E-14 | 3.32E-12 |
| IGSF6 | 1.08030779 | 3.65028101 | 8.326143966 | 8.37E-14 | 3.49E-12 |
| ANG | -1.37120957 | 5.700142655 | -8.322380646 | 8.55E-14 | 3.55E-12 |
| NOSTRIN | -1.17960217 | 5.033136892 | -8.316020811 | 8.86E-14 | 3.66E-12 |
| FCRL3 | 1.468780418 | 4.579563469 | 8.240622221 | 1.35E-13 | 5.41E-12 |
| RNASE4 | -1.24136121 | 4.799515967 | -8.233257514 | 1.40E-13 | 5.62E-12 |
| FN1 | 1.781249958 | 7.28403011 | 8.222273185 | 1.49E-13 | 5.94E-12 |
| LOC101928045 | -1.557479979 | 3.51220629 | -8.221208666 | 1.50E-13 | 5.96E-12 |
| C1QC | 1.35970467 | 8.589111356 | 8.211607212 | 1.58E-13 | 6.28E-12 |
| HMGCS2 | -1.054030387 | 4.31810209 | -8.206976013 | 1.62E-13 | 6.42E-12 |
| SERPINE2 | 1.037133289 | 4.583830271 | 8.184578991 | 1.84E-13 | 7.19E-12 |
| ITGAM | 1.485455446 | 5.308891666 | 8.155484997 | 2.16E-13 | 8.36E-12 |
| CCL2 | 1.548871225 | 7.75010096 | 8.142297452 | 2.32E-13 | 8.91E-12 |
| IL18 | 1.263342583 | 3.190820706 | 8.13618099 | 2.40E-13 | 9.17E-12 |
| TPD52L1 | -1.746899708 | 5.54343024 | -8.126818775 | 2.53E-13 | 9.59E-12 |
| APOC1 | 1.243429073 | 6.685967291 | 8.124496093 | 2.56E-13 | 9.69E-12 |
| FAS | 1.069790088 | 4.079498035 | 8.124114141 | 2.57E-13 | 9.70E-12 |
| PPP1R16A | -1.051318466 | 6.107479996 | -8.122072579 | 2.60E-13 | 9.77E-12 |
| CSF1R | 1.074030658 | 7.576732732 | 8.112014801 | 2.75E-13 | 1.03E-11 |
| KIAA1551 | 1.265769938 | 6.27421504 | 8.110672107 | 2.77E-13 | 1.04E-11 |
| SCGB3A1 | -1.180619993 | 6.166687757 | -8.105756111 | 2.84E-13 | 1.06E-11 |
| HIST1H2BO | 1.100207354 | 3.881203271 | 8.096791718 | 2.99E-13 | 1.12E-11 |
| SAMHD1 | 1.066908647 | 7.047772742 | 8.077540583 | 3.32E-13 | 1.22E-11 |
| PPP1R1B | -1.892345505 | 7.124928184 | -8.074159319 | 3.39E-13 | 1.25E-11 |
| BTN3A3 | 1.239442546 | 6.062408855 | 8.071363778 | 3.44E-13 | 1.26E-11 |
| CCL5 | 1.0220206 | 5.983287118 | 8.063405844 | 3.59E-13 | 1.31E-11 |
| GBP1 | 1.495627336 | 4.86072426 | 8.044809554 | 3.98E-13 | 1.44E-11 |
| GPR171 | 1.216357368 | 3.771185544 | 8.04396248 | 4.00E-13 | 1.45E-11 |
| EFCAB4A | -1.175493221 | 6.677939236 | -8.011337029 | 4.79E-13 | 1.71E-11 |
| C1QA | 1.447286254 | 8.360726124 | 7.992451408 | 5.31E-13 | 1.87E-11 |
| ANKRD22 | 1.481641261 | 4.242131175 | 7.990340132 | 5.38E-13 | 1.89E-11 |
| IL1B | 1.122175448 | 4.88503914 | 7.987187505 | 5.47E-13 | 1.92E-11 |
| PALMD | -1.110687814 | 4.912220506 | -7.976322648 | 5.81E-13 | 2.02E-11 |
| MNDA | 1.161277642 | 4.94612389 | 7.944534555 | 6.91E-13 | 2.37E-11 |
| CD22 | 1.251386386 | 5.731822511 | 7.934954223 | 7.29E-13 | 2.48E-11 |
| CD274 | 1.195029167 | 4.068393187 | 7.931554934 | 7.42E-13 | 2.52E-11 |
| PLAU | 1.146405046 | 5.730347244 | 7.927885092 | 7.58E-13 | 2.57E-11 |
| NCALD | -1.061336785 | 4.489549923 | -7.926473583 | 7.63E-13 | 2.58E-11 |
| TNFAIP2 | 1.051852036 | 7.350571876 | 7.926176001 | 7.65E-13 | 2.58E-11 |
| C5orf27 | -1.235794969 | 4.708621631 | -7.915074233 | 8.13E-13 | 2.73E-11 |
| C1QB | 1.557447242 | 8.644853924 | 7.893880711 | 9.13E-13 | 3.03E-11 |
| LOC101928429 | 1.000861606 | 3.09881575 | 7.861878227 | 1.09E-12 | 3.55E-11 |
| HCP5 | 1.105373483 | 5.980077407 | 7.84046229 | 1.22E-12 | 3.96E-11 |
| CCL18 | 2.259386663 | 6.420079256 | 7.832059424 | 1.28E-12 | 4.13E-11 |
| TMSB4X | 1.248599873 | 11.62959181 | 7.831914459 | 1.28E-12 | 4.13E-11 |
| TYROBP | 1.183343585 | 7.814586894 | 7.81344865 | 1.42E-12 | 4.53E-11 |
| HLA-B | 1.028636969 | 11.22219789 | 7.810395153 | 1.44E-12 | 4.59E-11 |
| NR1H3 | 1.274136921 | 6.336522524 | 7.779408998 | 1.71E-12 | 5.35E-11 |
| CD83 | 1.13881258 | 5.967393631 | 7.778346508 | 1.72E-12 | 5.37E-11 |
| CILP | 1.64519654 | 4.704863467 | 7.774639684 | 1.75E-12 | 5.43E-11 |
| AQP5 | -2.082908727 | 6.054413642 | -7.771801628 | 1.78E-12 | 5.50E-11 |
| BHLHE40 | 1.044838298 | 6.84415742 | 7.725486531 | 2.29E-12 | 6.88E-11 |
| HLA-A | 1.010731229 | 10.55893273 | 7.712297005 | 2.46E-12 | 7.36E-11 |
| AIM1 | 1.051784641 | 5.261225535 | 7.69932142 | 2.64E-12 | 7.88E-11 |
| CD163 | 1.0856896 | 5.888354726 | 7.697920251 | 2.66E-12 | 7.92E-11 |
| HP | -1.730920823 | 6.630941215 | -7.679919163 | 2.93E-12 | 8.69E-11 |
| GSAP | 1.078730941 | 4.000924418 | 7.673099127 | 3.04E-12 | 8.99E-11 |
| SFRP1 | -1.713468955 | 6.71595406 | -7.669104286 | 3.11E-12 | 9.13E-11 |
| LINC01215 | 1.185409335 | 2.413399179 | 7.661911901 | 3.23E-12 | 9.44E-11 |
| NCF2 | 1.43853059 | 5.342756996 | 7.654339727 | 3.37E-12 | 9.83E-11 |
| FAM3D | -1.133505299 | 6.916234235 | -7.644316664 | 3.56E-12 | 1.03E-10 |
| AQP1 | -1.102888301 | 7.10520768 | -7.59571941 | 4.62E-12 | 1.31E-10 |
| SYNM | -1.18865731 | 5.359752465 | -7.582753219 | 4.96E-12 | 1.40E-10 |
| CHRM1 | -1.102226971 | 3.596773627 | -7.572209707 | 5.25E-12 | 1.48E-10 |
| PLBD1 | 1.061830496 | 5.961734619 | 7.556144578 | 5.73E-12 | 1.61E-10 |
| SLC15A3 | 1.053860574 | 5.357592953 | 7.523906152 | 6.81E-12 | 1.88E-10 |
| CST2 | -2.191027755 | 6.547486487 | -7.515181415 | 7.14E-12 | 1.96E-10 |
| TNFRSF19 | -1.237064878 | 4.97480039 | -7.510371211 | 7.33E-12 | 2.00E-10 |
| LPO | -2.322013664 | 5.768748278 | -7.507703892 | 7.43E-12 | 2.03E-10 |
| FNDC1 | 1.869269185 | 4.36309401 | 7.501398569 | 7.69E-12 | 2.09E-10 |
| PLAUR | 1.007923117 | 5.286463648 | 7.496112917 | 7.91E-12 | 2.15E-10 |
| APCDD1L | -1.175221867 | 4.586975871 | -7.483675634 | 8.46E-12 | 2.29E-10 |
| KANK4 | -1.037985112 | 4.44159536 | -7.482274694 | 8.52E-12 | 2.30E-10 |
| LOC101928054 | 1.138517173 | 4.957958799 | 7.455171744 | 9.86E-12 | 2.63E-10 |
| ERP27 | 1.043079707 | 3.361334855 | 7.455088771 | 9.86E-12 | 2.63E-10 |
| TLR10 | 1.050470235 | 4.765983804 | 7.431683969 | 1.12E-11 | 2.96E-10 |
| MYOC | -1.214333262 | 3.917298673 | -7.426479814 | 1.15E-11 | 3.04E-10 |
| MOB1A | 1.00494349 | 5.239502354 | 7.425753194 | 1.15E-11 | 3.04E-10 |
| LZTS3 | -1.325589733 | 6.238466618 | -7.418714476 | 1.20E-11 | 3.14E-10 |
| MS4A1 | 1.950093811 | 5.076686494 | 7.408635151 | 1.27E-11 | 3.30E-10 |
| CHI3L1 | 1.727170696 | 6.046676181 | 7.406129465 | 1.28E-11 | 3.34E-10 |
| SLC13A2 | -1.150312527 | 5.409719521 | -7.391795026 | 1.38E-11 | 3.57E-10 |
| SULF1 | 1.323539794 | 6.147170733 | 7.389637066 | 1.40E-11 | 3.61E-10 |
| CSTA | 1.407216128 | 6.900331565 | 7.379933205 | 1.48E-11 | 3.78E-10 |
| EDN3 | -1.283298513 | 5.226788258 | -7.379643244 | 1.48E-11 | 3.78E-10 |
| VCAN | 1.59646028 | 6.10267031 | 7.365691681 | 1.59E-11 | 4.03E-10 |
| LY75 | 1.067428561 | 2.805011362 | 7.345663085 | 1.77E-11 | 4.45E-10 |
| LIPA | 1.379129287 | 6.954017718 | 7.344610146 | 1.78E-11 | 4.47E-10 |
| DCD | -1.178188215 | 3.186694187 | -7.325467003 | 1.97E-11 | 4.92E-10 |
| MAFB | 1.012670465 | 7.166983017 | 7.31335272 | 2.10E-11 | 5.21E-10 |
| KRT18 | -1.518636085 | 6.338836453 | -7.276525136 | 2.56E-11 | 6.22E-10 |
| EVI2A | 1.109104849 | 4.144953739 | 7.276138957 | 2.57E-11 | 6.23E-10 |
| LRRC15 | 1.30979638 | 4.763270732 | 7.260293002 | 2.79E-11 | 6.70E-10 |
| SLC7A7 | 1.026546832 | 5.133590326 | 7.240597773 | 3.10E-11 | 7.37E-10 |
| MSLN | -1.587806495 | 5.65437694 | -7.231708467 | 3.25E-11 | 7.68E-10 |
| LY96 | 1.119050794 | 5.659987007 | 7.224117153 | 3.38E-11 | 7.92E-10 |
| TCEAL2 | -1.549384577 | 4.757540501 | -7.220205963 | 3.45E-11 | 8.06E-10 |
| BTLA | 1.040738536 | 4.204557314 | 7.206087642 | 3.72E-11 | 8.61E-10 |
| OTX1 | -1.384271801 | 4.720180983 | -7.192370186 | 4.00E-11 | 9.18E-10 |
| TRAT1 | 1.145700021 | 3.196989622 | 7.188963188 | 4.07E-11 | 9.32E-10 |
| CAMSAP3 | -1.231800941 | 6.254892536 | -7.171208978 | 4.47E-11 | 1.02E-09 |
| KIF21A | -1.46620693 | 4.03284632 | -7.16129625 | 4.71E-11 | 1.07E-09 |
| PRO0471 | 1.203356105 | 4.336114945 | 7.156978164 | 4.82E-11 | 1.09E-09 |
| GIMAP7 | 1.000299028 | 5.444063687 | 7.131620364 | 5.51E-11 | 1.23E-09 |
| SCGB1D1 | -4.072830808 | 5.680889576 | -7.125413154 | 5.70E-11 | 1.27E-09 |
| CXCL10 | 1.944906467 | 6.262381456 | 7.107107938 | 6.27E-11 | 1.39E-09 |
| FAP | 1.112636406 | 4.744381002 | 7.097989366 | 6.58E-11 | 1.45E-09 |
| ASS1 | -1.079064153 | 7.685425769 | -7.0862785 | 7.00E-11 | 1.53E-09 |
| GCH1 | 1.280834053 | 4.197567304 | 7.081147152 | 7.19E-11 | 1.57E-09 |
| CDH11 | 1.000669175 | 4.500351082 | 7.066694644 | 7.76E-11 | 1.68E-09 |
| BLNK | 1.095790393 | 5.266722328 | 7.064107225 | 7.86E-11 | 1.70E-09 |
| LINC00948 | -1.580525066 | 3.453403485 | -7.049316706 | 8.50E-11 | 1.83E-09 |
| CST1 | -2.572654338 | 6.964452411 | -7.048219305 | 8.55E-11 | 1.83E-09 |
| SH3BGRL2 | -1.43246452 | 6.315212725 | -7.014548987 | 1.02E-10 | 2.15E-09 |
| GBP2 | 1.043575731 | 4.953087621 | 7.013571096 | 1.03E-10 | 2.16E-09 |
| TMEM125 | -1.051096706 | 5.325901585 | -7.012984472 | 1.03E-10 | 2.16E-09 |
| CRYAB | -1.621903008 | 7.614268481 | -6.955321742 | 1.39E-10 | 2.85E-09 |
| TIMD4 | 1.266176324 | 3.287824821 | 6.951296482 | 1.42E-10 | 2.90E-09 |
| WARS | 1.096857388 | 7.558282639 | 6.949721505 | 1.43E-10 | 2.92E-09 |
| SLAMF7 | 1.267209897 | 6.906352346 | 6.915154116 | 1.71E-10 | 3.43E-09 |
| KCNA3 | 1.076257773 | 5.386395273 | 6.913782296 | 1.73E-10 | 3.45E-09 |
| STAP1 | 1.015488418 | 3.745204451 | 6.896888128 | 1.88E-10 | 3.75E-09 |
| C15orf48 | 1.650881974 | 3.537844123 | 6.888747085 | 1.97E-10 | 3.89E-09 |
| GIMAP6 | 1.009272722 | 5.324717827 | 6.870347496 | 2.16E-10 | 4.25E-09 |
| MS4A4A | 1.000403714 | 4.981815306 | 6.863580197 | 2.24E-10 | 4.40E-09 |
| SCGB2A1 | -3.969295196 | 6.120845492 | -6.852911434 | 2.37E-10 | 4.61E-09 |
| TGFBI | 1.131440484 | 7.871641215 | 6.852908319 | 2.37E-10 | 4.61E-09 |
| ATP6V0A4 | -1.265639891 | 4.196432676 | -6.85217827 | 2.38E-10 | 4.62E-09 |
| TIMP1 | 1.176741904 | 8.866527826 | 6.81299857 | 2.91E-10 | 5.60E-09 |
| FCRL1 | 1.284206609 | 3.398780448 | 6.79552637 | 3.19E-10 | 6.07E-09 |
| RP11-532F12.5 | -1.159149626 | 5.558627266 | -6.78570071 | 3.35E-10 | 6.35E-09 |
| PFN2 | -1.281191774 | 6.870603623 | -6.765966001 | 3.71E-10 | 7.02E-09 |
| POSTN | 1.300153278 | 4.234440969 | 6.761153305 | 3.80E-10 | 7.17E-09 |
| GPNMB | 1.079866051 | 5.453484437 | 6.74403857 | 4.15E-10 | 7.77E-09 |
| SCGB2A2 | -1.948137703 | 3.644559047 | -6.723590296 | 4.61E-10 | 8.52E-09 |
| ROBO1 | 1.046829159 | 4.578224534 | 6.675855165 | 5.89E-10 | 1.06E-08 |
| LUM | 1.08425317 | 3.804788147 | 6.674229126 | 5.94E-10 | 1.07E-08 |
| DNASE2B | -1.488288985 | 3.804071109 | -6.663844795 | 6.27E-10 | 1.12E-08 |
| CRLF3 | 1.10975657 | 4.816574222 | 6.657151163 | 6.49E-10 | 1.16E-08 |
| GGT6 | -1.010228146 | 5.144401201 | -6.647195289 | 6.82E-10 | 1.22E-08 |
| CRNDE | -1.037569913 | 4.250717316 | -6.643146736 | 6.97E-10 | 1.24E-08 |
| CST4 | -2.809512387 | 7.277700794 | -6.63861176 | 7.13E-10 | 1.26E-08 |
| C5AR1 | 1.273665234 | 5.248168403 | 6.629196054 | 7.48E-10 | 1.32E-08 |
| SLC14A1 | -1.495932682 | 4.322980477 | -6.559152152 | 1.07E-09 | 1.83E-08 |
| SPP1 | 1.632772664 | 4.159506423 | 6.546483183 | 1.14E-09 | 1.94E-08 |
| COBL | -1.12811662 | 4.833034371 | -6.537523813 | 1.19E-09 | 2.01E-08 |
| KRT14 | -1.375014593 | 5.286629151 | -6.474539943 | 1.64E-09 | 2.67E-08 |
| OLR1 | 1.063439957 | 3.056161443 | 6.447438662 | 1.88E-09 | 3.03E-08 |
| TRIM22 | 1.311109349 | 5.272852584 | 6.42764956 | 2.08E-09 | 3.32E-08 |
| ESRRG | -1.016779775 | 3.155413709 | -6.422097179 | 2.14E-09 | 3.40E-08 |
| PRSS8 | -1.820316523 | 5.806280395 | -6.391133899 | 2.50E-09 | 3.92E-08 |
| DNER | -1.118649215 | 3.391315455 | -6.381865456 | 2.61E-09 | 4.09E-08 |
| PBX1 | -1.001232795 | 6.237519413 | -6.378937096 | 2.65E-09 | 4.15E-08 |
| MYO5C | -1.223851477 | 4.786747088 | -6.344385923 | 3.15E-09 | 4.85E-08 |
| PLCB1 | -1.177287192 | 4.870194436 | -6.332588096 | 3.35E-09 | 5.11E-08 |
| MUC15 | -1.058325879 | 3.544961942 | -6.294305014 | 4.05E-09 | 6.08E-08 |
| FOLR1 | -1.366058254 | 3.91642399 | -6.287013527 | 4.20E-09 | 6.29E-08 |
| FTL | 1.051898246 | 11.51370103 | 6.284189176 | 4.26E-09 | 6.37E-08 |
| DMBT1 | -2.524424251 | 7.406881013 | -6.266772866 | 4.64E-09 | 6.87E-08 |
| COL5A2 | 1.431215167 | 6.383299529 | 6.249329691 | 5.06E-09 | 7.43E-08 |
| PLEKHH1 | -1.34944453 | 4.217889469 | -6.232098578 | 5.51E-09 | 8.01E-08 |
| THBS2 | 1.206012419 | 5.681576899 | 6.203262674 | 6.36E-09 | 9.12E-08 |
| COL6A3 | 1.31680547 | 8.319185499 | 6.15901529 | 7.91E-09 | 1.11E-07 |
| HTN1 | -2.286265945 | 4.187103901 | -6.147682945 | 8.37E-09 | 1.16E-07 |
| CCL8 | 1.172693299 | 4.964375138 | 6.124154513 | 9.39E-09 | 1.29E-07 |
| VSIG10L | -1.377198506 | 5.205718229 | -6.12303764 | 9.44E-09 | 1.30E-07 |
| PAX6 | -1.594318777 | 4.948996695 | -6.082101389 | 1.15E-08 | 1.56E-07 |
| IGHM | 1.100337255 | 8.930057021 | 6.022697851 | 1.54E-08 | 2.03E-07 |
| CTSK | 1.398425511 | 6.683633321 | 6.014881554 | 1.60E-08 | 2.10E-07 |
| MXRA5 | 1.085894936 | 7.302876465 | 6.014240878 | 1.61E-08 | 2.10E-07 |
| SOX10 | -1.380467368 | 6.273523206 | -5.999879014 | 1.72E-08 | 2.24E-07 |
| TFCP2L1 | -1.108655257 | 4.937314866 | -5.992442631 | 1.79E-08 | 2.31E-07 |
| HSD11B1 | 1.140793615 | 4.690144168 | 5.990561981 | 1.80E-08 | 2.33E-07 |
| ANXA3 | -1.23988407 | 4.134260438 | -5.963195806 | 2.06E-08 | 2.64E-07 |
| C16orf54 | 1.143124797 | 3.16505237 | 5.952602121 | 2.17E-08 | 2.76E-07 |
| RNF152 | -1.079430966 | 3.889505654 | -5.950032673 | 2.19E-08 | 2.79E-07 |
| FOXC1 | -1.34008042 | 7.431855157 | -5.948084135 | 2.21E-08 | 2.82E-07 |
| STATH | -2.37644174 | 4.301394305 | -5.93262143 | 2.39E-08 | 3.01E-07 |
| MLPH | -1.432736474 | 6.901743623 | -5.91946421 | 2.54E-08 | 3.19E-07 |
| DSG2 | -1.161236929 | 4.817334091 | -5.905485278 | 2.72E-08 | 3.39E-07 |
| SOX9 | -1.366197148 | 6.561130767 | -5.891075628 | 2.92E-08 | 3.61E-07 |
| S100A8 | 1.015741623 | 4.067288789 | 5.856836398 | 3.44E-08 | 4.18E-07 |
| HTRA4 | 1.196242759 | 3.953595575 | 5.842954639 | 3.67E-08 | 4.45E-07 |
| AQP3 | -1.108866675 | 6.303021651 | -5.832842415 | 3.85E-08 | 4.64E-07 |
| LOC100507073 | -1.683469502 | 3.962866542 | -5.820442569 | 4.09E-08 | 4.89E-07 |
| FAM3B | -1.207216953 | 4.887548894 | -5.801512847 | 4.48E-08 | 5.31E-07 |
| PPL | -1.14554451 | 5.968105556 | -5.795536525 | 4.61E-08 | 5.45E-07 |
| LACRT | -3.055063754 | 8.357458034 | -5.788722831 | 4.76E-08 | 5.62E-07 |
| ADRA2A | 1.129951 | 4.780602229 | 5.78702416 | 4.80E-08 | 5.65E-07 |
| KIT | -1.063335971 | 5.010092407 | -5.703980446 | 7.11E-08 | 8.12E-07 |
| ESRP1 | -1.203238443 | 3.627411562 | -5.674372773 | 8.18E-08 | 9.22E-07 |
| TCN1 | -2.509353562 | 5.448520881 | -5.666984073 | 8.47E-08 | 9.49E-07 |
| MMP12 | 1.3705092 | 3.119920723 | 5.658499879 | 8.81E-08 | 9.82E-07 |
| C8orf4 | -1.514511365 | 4.168743599 | -5.629434093 | 1.01E-07 | 1.11E-06 |
| ELF5 | -1.409770038 | 4.880438124 | -5.613057184 | 1.09E-07 | 1.19E-06 |
| STK39 | -1.417392348 | 5.864480138 | -5.59786058 | 1.17E-07 | 1.27E-06 |
| PHLDA1 | -1.133496992 | 5.710860527 | -5.570535808 | 1.33E-07 | 1.43E-06 |
| COL1A2 | 1.315830346 | 7.558188656 | 5.542845569 | 1.51E-07 | 1.61E-06 |
| COL1A1 | 1.321354189 | 8.810400029 | 5.534623703 | 1.57E-07 | 1.67E-06 |
| C2orf40 | -1.04176281 | 3.979899039 | -5.528722644 | 1.62E-07 | 1.71E-06 |
| CTHRC1 | 1.118360798 | 4.603545236 | 5.503325266 | 1.82E-07 | 1.91E-06 |
| CYP27B1 | 1.068454391 | 3.519433051 | 5.499739991 | 1.85E-07 | 1.94E-06 |
| C9orf152 | -1.137525089 | 2.934158735 | -5.487635906 | 1.96E-07 | 2.04E-06 |
| CYTIP | 1.131152082 | 5.779786253 | 5.478464432 | 2.04E-07 | 2.12E-06 |
| HOMER2 | -1.21620279 | 3.927256111 | -5.435189816 | 2.49E-07 | 2.54E-06 |
| EPCAM | -1.411146825 | 4.440983709 | -5.403608606 | 2.88E-07 | 2.90E-06 |
| FXYD3 | -1.336026517 | 5.975470206 | -5.382422371 | 3.17E-07 | 3.18E-06 |
| C4orf19 | -1.044819461 | 4.496073824 | -5.38187738 | 3.18E-07 | 3.19E-06 |
| IRX3 | -1.166271678 | 4.800317243 | -5.323175477 | 4.15E-07 | 4.07E-06 |
| COL5A1 | 1.182860268 | 7.165257779 | 5.308120305 | 4.45E-07 | 4.33E-06 |
| COL3A1 | 1.344000724 | 8.086316325 | 5.305865768 | 4.49E-07 | 4.36E-06 |
| HSD17B2 | -1.367539942 | 4.810730615 | -5.226958208 | 6.42E-07 | 6.04E-06 |
| MEIS1 | -1.314464706 | 6.132674233 | -5.21395705 | 6.80E-07 | 6.37E-06 |
| KLF5 | -1.154490116 | 5.43950482 | -5.178668738 | 7.97E-07 | 7.37E-06 |
| SLC12A2 | -1.270642383 | 5.702350445 | -5.165184962 | 8.46E-07 | 7.79E-06 |
| CA2 | -1.13845833 | 3.629300297 | -5.11501163 | 1.06E-06 | 9.51E-06 |
| LCN1 | -2.755375603 | 9.497904003 | -5.09087691 | 1.18E-06 | 1.05E-05 |
| PLA2G2A | -1.190416601 | 9.498741971 | -4.99310113 | 1.81E-06 | 1.54E-05 |
| MEIS2 | -1.012826361 | 4.201521367 | -4.9597231 | 2.09E-06 | 1.76E-05 |
| KIAA1324 | -1.34289292 | 5.294225462 | -4.907934057 | 2.62E-06 | 2.16E-05 |
| MMP10 | 1.200411122 | 3.832151799 | 4.887266209 | 2.86E-06 | 2.34E-05 |
| ZG16B | -1.698413915 | 8.023607994 | -4.886150415 | 2.88E-06 | 2.35E-05 |
| TFAP2A | -1.053556391 | 4.569192731 | -4.855780588 | 3.28E-06 | 2.65E-05 |
| AZGP1 | -2.424857276 | 7.654945649 | -4.815802396 | 3.89E-06 | 3.08E-05 |
| CLDN10 | -1.293842379 | 6.449223664 | -4.741850583 | 5.33E-06 | 4.10E-05 |
| APOD | -1.106323736 | 9.376270386 | -4.612937817 | 9.14E-06 | 6.65E-05 |
| PLIN1 | -1.438196357 | 5.343827656 | -4.558729618 | 1.14E-05 | 8.11E-05 |
| EHF | -1.541381663 | 5.41032821 | -4.555517408 | 1.16E-05 | 8.21E-05 |
| IFI44L | 1.002362728 | 4.182915332 | 4.516017503 | 1.36E-05 | 9.51E-05 |
| ODAM | -1.413323655 | 5.31497353 | -4.425475276 | 1.97E-05 | 0.000133235 |
| CCL28 | -1.40432103 | 6.127705409 | -4.314004136 | 3.08E-05 | 0.000200156 |
| CHIT1 | 1.086714573 | 5.468413762 | 4.277339593 | 3.56E-05 | 0.000227628 |
| PIP | -2.445171248 | 8.057139749 | -4.269214065 | 3.68E-05 | 0.000234082 |
| CDH1 | -1.074858558 | 6.070377709 | -4.224118648 | 4.39E-05 | 0.000273781 |
| SLC31A2 | -1.000093694 | 4.760033067 | -4.179732694 | 5.22E-05 | 0.000317621 |
| SLPI | -1.499722064 | 9.03898103 | -4.179651682 | 5.22E-05 | 0.000317632 |
| KRT5 | -1.117444856 | 6.239255786 | -4.175797353 | 5.30E-05 | 0.000321796 |
| SMR3B | -2.295617482 | 7.392208412 | -4.063684882 | 8.17E-05 | 0.000475328 |
| KRT23 | -1.29999245 | 6.870446082 | -4.058682034 | 8.33E-05 | 0.000483056 |
| HLA-DQA1 | 1.854379664 | 7.405003525 | 3.99325754 | 0.000106689 | 0.000602252 |
| PDZK1IP1 | -1.045708557 | 6.318479442 | -3.918567198 | 0.000141126 | 0.00077229 |
| SLC6A14 | -1.042569376 | 4.296239042 | -3.730673794 | 0.00028062 | 0.001417102 |
| FDCSP | 1.640749897 | 6.895699682 | 3.674400162 | 0.000343169 | 0.001696184 |
| CXCL17 | -1.59396707 | 7.134648473 | -3.645518506 | 0.000380185 | 0.001855426 |
| LCN2 | -1.140560608 | 6.883723595 | -3.554841395 | 0.000522449 | 0.0024408 |
| PROM1 | -1.232918975 | 5.149411498 | -3.091619938 | 0.002420325 | 0.009240077 |
| OLFM4 | 1.007597716 | 3.991164267 | 2.999712409 | 0.003220635 | 0.011718977 |
| PIGR | -1.490801813 | 8.732908525 | -2.962513666 | 0.003608972 | 0.012896247 |
| SMR3A | -1.670320409 | 7.709410345 | -2.818140084 | 0.005559232 | 0.018451811 |

# Appendix 5

**Analysis of enrichment**

**Table S5a. Analysis of GO.**

| ONTOLOGY | ID | Description | GeneRatio | pvalue | qvalue |
| --- | --- | --- | --- | --- | --- |
| BP | GO:0002443 | leukocyte mediated immunity | 59/402 | 3.71E-32 | 1.03E-28 |
| BP | GO:0007159 | leukocyte cell-cell adhesion | 54/402 | 9.50E-27 | 1.31E-23 |
| BP | GO:0070661 | leukocyte proliferation | 47/402 | 4.64E-24 | 4.28E-21 |
| BP | GO:0046651 | lymphocyte proliferation | 44/402 | 1.08E-23 | 7.45E-21 |
| BP | GO:0002768 | immune response-regulating cell surface receptor signaling pathway | 45/402 | 2.68E-23 | 1.27E-20 |
| BP | GO:0032943 | mononuclear cell proliferation | 44/402 | 2.75E-23 | 1.27E-20 |
| BP | GO:0002449 | lymphocyte mediated immunity | 43/402 | 4.06E-23 | 1.60E-20 |
| BP | GO:0002764 | immune response-regulating signaling pathway | 51/402 | 1.64E-22 | 5.12E-20 |
| BP | GO:1903037 | regulation of leukocyte cell-cell adhesion | 47/402 | 1.67E-22 | 5.12E-20 |
| BP | GO:0070663 | regulation of leukocyte proliferation | 40/402 | 3.13E-22 | 8.64E-20 |
| BP | GO:0002683 | negative regulation of immune system process | 52/402 | 9.47E-22 | 2.25E-19 |
| BP | GO:0050867 | positive regulation of cell activation | 47/402 | 9.77E-22 | 2.25E-19 |
| BP | GO:0002696 | positive regulation of leukocyte activation | 46/402 | 1.07E-21 | 2.27E-19 |
| BP | GO:0022407 | regulation of cell-cell adhesion | 52/402 | 1.37E-21 | 2.56E-19 |
| BP | GO:0002460 | adaptive immune response based on somatic recombination of immune receptors built from immunoglobulin superfamily domains | 42/402 | 1.39E-21 | 2.56E-19 |
| BP | GO:0050670 | regulation of lymphocyte proliferation | 37/402 | 3.38E-21 | 5.75E-19 |
| BP | GO:0002429 | immune response-activating cell surface receptor signaling pathway | 41/402 | 3.54E-21 | 5.75E-19 |
| BP | GO:0032944 | regulation of mononuclear cell proliferation | 37/402 | 6.09E-21 | 9.35E-19 |
| BP | GO:0019221 | cytokine-mediated signaling pathway | 51/402 | 9.05E-21 | 1.32E-18 |
| BP | GO:1903131 | mononuclear cell differentiation | 50/402 | 1.05E-20 | 1.45E-18 |
| BP | GO:0050900 | leukocyte migration | 45/402 | 3.98E-20 | 5.11E-18 |
| BP | GO:0001819 | positive regulation of cytokine production | 50/402 | 4.06E-20 | 5.11E-18 |
| BP | GO:1903039 | positive regulation of leukocyte cell-cell adhesion | 38/402 | 4.41E-20 | 5.30E-18 |
| BP | GO:0050863 | regulation of T cell activation | 44/402 | 5.04E-20 | 5.81E-18 |
| BP | GO:0002253 | activation of immune response | 50/402 | 6.89E-20 | 7.62E-18 |
| BP | GO:0050851 | antigen receptor-mediated signaling pathway | 33/402 | 7.86E-20 | 8.36E-18 |
| BP | GO:0002366 | leukocyte activation involved in immune response | 39/402 | 1.01E-19 | 1.04E-17 |
| BP | GO:0002757 | immune response-activating signaling pathway | 46/402 | 1.21E-19 | 1.20E-17 |
| BP | GO:0002263 | cell activation involved in immune response | 39/402 | 1.64E-19 | 1.57E-17 |
| BP | GO:0022409 | positive regulation of cell-cell adhesion | 40/402 | 2.59E-19 | 2.38E-17 |
| BP | GO:0051251 | positive regulation of lymphocyte activation | 40/402 | 5.07E-19 | 4.52E-17 |
| BP | GO:0030098 | lymphocyte differentiation | 45/402 | 6.76E-19 | 5.84E-17 |
| BP | GO:0050870 | positive regulation of T cell activation | 35/402 | 1.02E-18 | 8.56E-17 |
| BP | GO:0001909 | leukocyte mediated cytotoxicity | 27/402 | 2.20E-18 | 1.79E-16 |
| BP | GO:0002274 | myeloid leukocyte activation | 34/402 | 2.54E-18 | 2.01E-16 |
| BP | GO:0046631 | alpha-beta T cell activation | 29/402 | 1.11E-17 | 8.55E-16 |
| BP | GO:0030595 | leukocyte chemotaxis | 33/402 | 2.33E-17 | 1.74E-15 |
| BP | GO:0070665 | positive regulation of leukocyte proliferation | 28/402 | 2.55E-17 | 1.85E-15 |
| BP | GO:0060326 | cell chemotaxis | 37/402 | 3.21E-17 | 2.27E-15 |
| BP | GO:0045785 | positive regulation of cell adhesion | 45/402 | 1.15E-16 | 7.94E-15 |
| BP | GO:0001906 | cell killing | 29/402 | 2.99E-16 | 2.02E-14 |
| BP | GO:0042098 | T cell proliferation | 30/402 | 3.15E-16 | 2.07E-14 |
| BP | GO:0071674 | mononuclear cell migration | 29/402 | 5.93E-16 | 3.81E-14 |
| BP | GO:0002237 | response to molecule of bacterial origin | 38/402 | 1.01E-15 | 6.35E-14 |
| BP | GO:0050852 | T cell receptor signaling pathway | 24/402 | 3.46E-15 | 2.13E-13 |
| BP | GO:0097530 | granulocyte migration | 25/402 | 4.66E-15 | 2.80E-13 |
| BP | GO:0002695 | negative regulation of leukocyte activation | 28/402 | 4.80E-15 | 2.80E-13 |
| BP | GO:0002697 | regulation of immune effector process | 38/402 | 4.86E-15 | 2.80E-13 |
| BP | GO:0042113 | B cell activation | 32/402 | 9.21E-15 | 5.19E-13 |
| BP | GO:0097529 | myeloid leukocyte migration | 30/402 | 9.56E-15 | 5.29E-13 |
| BP | GO:0050866 | negative regulation of cell activation | 29/402 | 1.08E-14 | 5.84E-13 |
| BP | GO:0002703 | regulation of leukocyte mediated immunity | 30/402 | 1.67E-14 | 8.88E-13 |
| BP | GO:0042129 | regulation of T cell proliferation | 26/402 | 2.56E-14 | 1.33E-12 |
| BP | GO:0032496 | response to lipopolysaccharide | 35/402 | 2.91E-14 | 1.49E-12 |
| BP | GO:1990266 | neutrophil migration | 22/402 | 4.73E-14 | 2.37E-12 |
| BP | GO:1903706 | regulation of hemopoiesis | 38/402 | 5.86E-14 | 2.89E-12 |
| BP | GO:0042742 | defense response to bacterium | 33/402 | 5.99E-14 | 2.90E-12 |
| BP | GO:0050671 | positive regulation of lymphocyte proliferation | 23/402 | 7.42E-14 | 3.54E-12 |
| BP | GO:0071621 | granulocyte chemotaxis | 22/402 | 7.77E-14 | 3.64E-12 |
| BP | GO:0043299 | leukocyte degranulation | 18/402 | 9.42E-14 | 4.34E-12 |
| BP | GO:0030217 | T cell differentiation | 32/402 | 9.67E-14 | 4.38E-12 |
| BP | GO:0032946 | positive regulation of mononuclear cell proliferation | 23/402 | 1.17E-13 | 5.20E-12 |
| BP | GO:0030593 | neutrophil chemotaxis | 20/402 | 1.30E-13 | 5.72E-12 |
| BP | GO:1902105 | regulation of leukocyte differentiation | 32/402 | 5.28E-13 | 2.28E-11 |
| BP | GO:0050864 | regulation of B cell activation | 21/402 | 6.36E-13 | 2.71E-11 |
| BP | GO:0016064 | immunoglobulin mediated immune response | 22/402 | 6.65E-13 | 2.79E-11 |
| BP | GO:0032609 | type II interferon production | 20/402 | 8.96E-13 | 3.64E-11 |
| BP | GO:0032649 | regulation of type II interferon production | 20/402 | 8.96E-13 | 3.64E-11 |
| BP | GO:0002699 | positive regulation of immune effector process | 29/402 | 9.18E-13 | 3.68E-11 |
| BP | GO:0042102 | positive regulation of T cell proliferation | 19/402 | 9.91E-13 | 3.92E-11 |
| BP | GO:0019724 | B cell mediated immunity | 22/402 | 1.02E-12 | 3.97E-11 |
| BP | GO:0050727 | regulation of inflammatory response | 36/402 | 1.30E-12 | 4.98E-11 |
| BP | GO:0051250 | negative regulation of lymphocyte activation | 23/402 | 1.81E-12 | 6.87E-11 |
| BP | GO:0002444 | myeloid leukocyte mediated immunity | 19/402 | 2.36E-12 | 8.83E-11 |
| BP | GO:0034341 | response to type II interferon | 21/402 | 2.44E-12 | 8.99E-11 |
| BP | GO:0002275 | myeloid cell activation involved in immune response | 18/402 | 3.03E-12 | 1.10E-10 |
| BP | GO:1990868 | response to chemokine | 18/402 | 3.63E-12 | 1.29E-10 |
| BP | GO:1990869 | cellular response to chemokine | 18/402 | 3.63E-12 | 1.29E-10 |
| BP | GO:0002831 | regulation of response to biotic stimulus | 37/402 | 7.75E-12 | 2.71E-10 |
| BP | GO:0007162 | negative regulation of cell adhesion | 30/402 | 8.51E-12 | 2.94E-10 |
| BP | GO:0002228 | natural killer cell mediated immunity | 16/402 | 1.04E-11 | 3.54E-10 |
| BP | GO:0045576 | mast cell activation | 15/402 | 1.57E-11 | 5.27E-10 |
| BP | GO:0072676 | lymphocyte migration | 19/402 | 1.58E-11 | 5.27E-10 |
| BP | GO:0002685 | regulation of leukocyte migration | 25/402 | 2.62E-11 | 8.64E-10 |
| BP | GO:0050777 | negative regulation of immune response | 23/402 | 2.69E-11 | 8.73E-10 |
| BP | GO:0045088 | regulation of innate immune response | 32/402 | 3.46E-11 | 1.11E-09 |
| BP | GO:0035710 | CD4-positive, alpha-beta T cell activation | 18/402 | 5.01E-11 | 1.59E-09 |
| BP | GO:0071346 | cellular response to type II interferon | 18/402 | 5.81E-11 | 1.81E-09 |
| BP | GO:0050672 | negative regulation of lymphocyte proliferation | 16/402 | 5.83E-11 | 1.81E-09 |
| BP | GO:0071219 | cellular response to molecule of bacterial origin | 25/402 | 6.72E-11 | 2.06E-09 |
| BP | GO:0042267 | natural killer cell mediated cytotoxicity | 15/402 | 6.85E-11 | 2.08E-09 |
| BP | GO:0032945 | negative regulation of mononuclear cell proliferation | 16/402 | 6.97E-11 | 2.10E-09 |
| BP | GO:0032729 | positive regulation of type II interferon production | 15/402 | 8.34E-11 | 2.45E-09 |
| BP | GO:0050853 | B cell receptor signaling pathway | 15/402 | 8.34E-11 | 2.45E-09 |
| BP | GO:0050854 | regulation of antigen receptor-mediated signaling pathway | 14/402 | 8.60E-11 | 2.50E-09 |
| BP | GO:0070098 | chemokine-mediated signaling pathway | 16/402 | 9.89E-11 | 2.85E-09 |
| BP | GO:0071222 | cellular response to lipopolysaccharide | 24/402 | 1.18E-10 | 3.35E-09 |
| BP | GO:0071216 | cellular response to biotic stimulus | 26/402 | 1.28E-10 | 3.62E-09 |
| BP | GO:0071675 | regulation of mononuclear cell migration | 18/402 | 1.37E-10 | 3.84E-09 |
| BP | GO:0031349 | positive regulation of defense response | 34/402 | 1.50E-10 | 4.13E-09 |
| BP | GO:0002285 | lymphocyte activation involved in immune response | 23/402 | 1.53E-10 | 4.20E-09 |
| BP | GO:0043410 | positive regulation of MAPK cascade | 36/402 | 2.14E-10 | 5.79E-09 |
| BP | GO:0070664 | negative regulation of leukocyte proliferation | 16/402 | 2.28E-10 | 6.12E-09 |
| BP | GO:0072678 | T cell migration | 14/402 | 3.02E-10 | 8.02E-09 |
| BP | GO:0006909 | phagocytosis | 24/402 | 3.48E-10 | 9.15E-09 |
| BP | GO:0070371 | ERK1 and ERK2 cascade | 29/402 | 3.65E-10 | 9.53E-09 |
| BP | GO:0046634 | regulation of alpha-beta T cell activation | 17/402 | 3.79E-10 | 9.79E-09 |
| BP | GO:0002478 | antigen processing and presentation of exogenous peptide antigen | 11/402 | 5.48E-10 | 1.40E-08 |
| BP | GO:0071887 | leukocyte apoptotic process | 17/402 | 5.75E-10 | 1.46E-08 |
| BP | GO:0042100 | B cell proliferation | 16/402 | 6.74E-10 | 1.69E-08 |
| BP | GO:0002819 | regulation of adaptive immune response | 22/402 | 6.88E-10 | 1.71E-08 |
| BP | GO:2000106 | regulation of leukocyte apoptotic process | 15/402 | 8.38E-10 | 2.07E-08 |
| BP | GO:0030183 | B cell differentiation | 19/402 | 8.67E-10 | 2.12E-08 |
| BP | GO:0048247 | lymphocyte chemotaxis | 13/402 | 8.88E-10 | 2.15E-08 |
| BP | GO:0045055 | regulated exocytosis | 23/402 | 9.57E-10 | 2.30E-08 |
| BP | GO:0002822 | regulation of adaptive immune response based on somatic recombination of immune receptors built from immunoglobulin superfamily domains | 21/402 | 9.84E-10 | 2.34E-08 |
| BP | GO:0006959 | humoral immune response | 24/402 | 1.13E-09 | 2.66E-08 |
| BP | GO:0045619 | regulation of lymphocyte differentiation | 22/402 | 1.31E-09 | 3.06E-08 |
| BP | GO:0071356 | cellular response to tumor necrosis factor | 23/402 | 1.73E-09 | 4.02E-08 |
| BP | GO:0001910 | regulation of leukocyte mediated cytotoxicity | 15/402 | 1.83E-09 | 4.22E-08 |
| BP | GO:0034612 | response to tumor necrosis factor | 24/402 | 1.97E-09 | 4.49E-08 |
| BP | GO:0002279 | mast cell activation involved in immune response | 12/402 | 2.07E-09 | 4.69E-08 |
| BP | GO:0031295 | T cell costimulation | 11/402 | 2.18E-09 | 4.91E-08 |
| BP | GO:0002448 | mast cell mediated immunity | 12/402 | 2.57E-09 | 5.74E-08 |
| BP | GO:0002440 | production of molecular mediator of immune response | 27/402 | 3.06E-09 | 6.77E-08 |
| BP | GO:0019882 | antigen processing and presentation | 16/402 | 3.11E-09 | 6.83E-08 |
| BP | GO:0048002 | antigen processing and presentation of peptide antigen | 13/402 | 3.43E-09 | 7.46E-08 |
| BP | GO:0031294 | lymphocyte costimulation | 11/402 | 3.60E-09 | 7.79E-08 |
| BP | GO:0046635 | positive regulation of alpha-beta T cell activation | 13/402 | 4.10E-09 | 8.78E-08 |
| BP | GO:0070372 | regulation of ERK1 and ERK2 cascade | 26/402 | 5.71E-09 | 1.21E-07 |
| BP | GO:0019884 | antigen processing and presentation of exogenous antigen | 11/402 | 5.80E-09 | 1.22E-07 |
| BP | GO:0002286 | T cell activation involved in immune response | 16/402 | 5.89E-09 | 1.23E-07 |
| BP | GO:0051651 | maintenance of location in cell | 22/402 | 7.65E-09 | 1.59E-07 |
| BP | GO:2000514 | regulation of CD4-positive, alpha-beta T cell activation | 13/402 | 9.63E-09 | 1.99E-07 |
| BP | GO:0032102 | negative regulation of response to external stimulus | 31/402 | 1.18E-08 | 2.41E-07 |
| BP | GO:0019886 | antigen processing and presentation of exogenous peptide antigen via MHC class II | 9/402 | 1.24E-08 | 2.52E-07 |
| BP | GO:0030888 | regulation of B cell proliferation | 12/402 | 1.25E-08 | 2.53E-07 |
| BP | GO:0002688 | regulation of leukocyte chemotaxis | 16/402 | 1.36E-08 | 2.73E-07 |
| BP | GO:0031341 | regulation of cell killing | 15/402 | 1.45E-08 | 2.89E-07 |
| BP | GO:0038093 | Fc receptor signaling pathway | 11/402 | 1.73E-08 | 3.39E-07 |
| BP | GO:0043303 | mast cell degranulation | 11/402 | 1.73E-08 | 3.39E-07 |
| BP | GO:0002706 | regulation of lymphocyte mediated immunity | 19/402 | 1.92E-08 | 3.73E-07 |
| BP | GO:0002687 | positive regulation of leukocyte migration | 17/402 | 2.00E-08 | 3.86E-07 |
| BP | GO:0022408 | negative regulation of cell-cell adhesion | 20/402 | 2.13E-08 | 4.09E-07 |
| BP | GO:0002705 | positive regulation of leukocyte mediated immunity | 17/402 | 2.21E-08 | 4.22E-07 |
| BP | GO:0036336 | dendritic cell migration | 9/402 | 2.28E-08 | 4.31E-07 |
| BP | GO:2000516 | positive regulation of CD4-positive, alpha-beta T cell activation | 10/402 | 2.54E-08 | 4.78E-07 |
| BP | GO:1903038 | negative regulation of leukocyte cell-cell adhesion | 17/402 | 2.71E-08 | 5.06E-07 |
| BP | GO:0002700 | regulation of production of molecular mediator of immune response | 19/402 | 2.97E-08 | 5.51E-07 |
| BP | GO:0030101 | natural killer cell activation | 14/402 | 3.14E-08 | 5.78E-07 |
| BP | GO:0032693 | negative regulation of interleukin-10 production | 8/402 | 3.46E-08 | 6.34E-07 |
| BP | GO:0050830 | defense response to Gram-positive bacterium | 15/402 | 3.82E-08 | 6.95E-07 |
| BP | GO:0002495 | antigen processing and presentation of peptide antigen via MHC class II | 9/402 | 4.01E-08 | 7.21E-07 |
| BP | GO:0050729 | positive regulation of inflammatory response | 17/402 | 4.02E-08 | 7.21E-07 |
| BP | GO:0001818 | negative regulation of cytokine production | 27/402 | 4.15E-08 | 7.40E-07 |
| BP | GO:0046632 | alpha-beta T cell differentiation | 15/402 | 4.29E-08 | 7.60E-07 |
| BP | GO:0001776 | leukocyte homeostasis | 14/402 | 4.61E-08 | 8.11E-07 |
| BP | GO:0002407 | dendritic cell chemotaxis | 8/402 | 4.91E-08 | 8.58E-07 |
| BP | GO:0006887 | exocytosis | 26/402 | 5.22E-08 | 9.07E-07 |
| BP | GO:0043367 | CD4-positive, alpha-beta T cell differentiation | 13/402 | 5.83E-08 | 1.01E-06 |
| BP | GO:0002698 | negative regulation of immune effector process | 15/402 | 6.01E-08 | 1.03E-06 |
| BP | GO:0031348 | negative regulation of defense response | 23/402 | 6.23E-08 | 1.06E-06 |
| BP | GO:0002504 | antigen processing and presentation of peptide or polysaccharide antigen via MHC class II | 9/402 | 6.81E-08 | 1.15E-06 |
| BP | GO:0010818 | T cell chemotaxis | 8/402 | 6.84E-08 | 1.15E-06 |
| BP | GO:0150076 | neuroinflammatory response | 12/402 | 7.80E-08 | 1.31E-06 |
| BP | GO:0031589 | cell-substrate adhesion | 26/402 | 8.18E-08 | 1.36E-06 |
| BP | GO:0002269 | leukocyte activation involved in inflammatory response | 10/402 | 9.38E-08 | 1.55E-06 |
| BP | GO:0042116 | macrophage activation | 14/402 | 9.54E-08 | 1.57E-06 |
| BP | GO:1902107 | positive regulation of leukocyte differentiation | 18/402 | 1.04E-07 | 1.70E-06 |
| BP | GO:1903708 | positive regulation of hemopoiesis | 18/402 | 1.04E-07 | 1.70E-06 |
| BP | GO:0045824 | negative regulation of innate immune response | 12/402 | 1.05E-07 | 1.70E-06 |
| BP | GO:0070227 | lymphocyte apoptotic process | 12/402 | 1.40E-07 | 2.25E-06 |
| BP | GO:0002690 | positive regulation of leukocyte chemotaxis | 13/402 | 1.46E-07 | 2.33E-06 |
| BP | GO:0032613 | interleukin-10 production | 11/402 | 1.54E-07 | 2.43E-06 |
| BP | GO:0032653 | regulation of interleukin-10 production | 11/402 | 1.54E-07 | 2.43E-06 |
| BP | GO:0050868 | negative regulation of T cell activation | 15/402 | 1.56E-07 | 2.45E-06 |
| BP | GO:0002526 | acute inflammatory response | 14/402 | 1.69E-07 | 2.63E-06 |
| BP | GO:0071706 | tumor necrosis factor superfamily cytokine production | 18/402 | 1.70E-07 | 2.63E-06 |
| BP | GO:1903555 | regulation of tumor necrosis factor superfamily cytokine production | 18/402 | 1.70E-07 | 2.63E-06 |
| BP | GO:0048015 | phosphatidylinositol-mediated signaling | 17/402 | 2.04E-07 | 3.13E-06 |
| BP | GO:0002704 | negative regulation of leukocyte mediated immunity | 11/402 | 2.11E-07 | 3.23E-06 |
| BP | GO:0048872 | homeostasis of number of cells | 23/402 | 2.14E-07 | 3.24E-06 |
| BP | GO:0042119 | neutrophil activation | 9/402 | 2.22E-07 | 3.35E-06 |
| BP | GO:0006816 | calcium ion transport | 29/402 | 2.25E-07 | 3.39E-06 |
| BP | GO:0061900 | glial cell activation | 10/402 | 2.43E-07 | 3.63E-06 |
| BP | GO:0007160 | cell-matrix adhesion | 20/402 | 2.70E-07 | 4.01E-06 |
| BP | GO:0048017 | inositol lipid-mediated signaling | 17/402 | 2.84E-07 | 4.20E-06 |
| BP | GO:2000107 | negative regulation of leukocyte apoptotic process | 10/402 | 2.90E-07 | 4.26E-06 |
| BP | GO:0001894 | tissue homeostasis | 21/402 | 2.98E-07 | 4.33E-06 |
| BP | GO:0060249 | anatomical structure homeostasis | 21/402 | 2.98E-07 | 4.33E-06 |
| BP | GO:0002548 | monocyte chemotaxis | 11/402 | 3.33E-07 | 4.82E-06 |
| BP | GO:0051924 | regulation of calcium ion transport | 21/402 | 3.59E-07 | 5.17E-06 |
| BP | GO:0070374 | positive regulation of ERK1 and ERK2 cascade | 19/402 | 3.80E-07 | 5.44E-06 |
| BP | GO:0038094 | Fc-gamma receptor signaling pathway | 8/402 | 3.82E-07 | 5.44E-06 |
| BP | GO:0071677 | positive regulation of mononuclear cell migration | 11/402 | 3.86E-07 | 5.46E-06 |
| BP | GO:0002824 | positive regulation of adaptive immune response based on somatic recombination of immune receptors built from immunoglobulin superfamily domains | 14/402 | 4.37E-07 | 6.16E-06 |
| BP | GO:0001913 | T cell mediated cytotoxicity | 10/402 | 4.83E-07 | 6.74E-06 |
| BP | GO:0070228 | regulation of lymphocyte apoptotic process | 10/402 | 4.83E-07 | 6.74E-06 |
| BP | GO:0043372 | positive regulation of CD4-positive, alpha-beta T cell differentiation | 8/402 | 4.90E-07 | 6.77E-06 |
| BP | GO:0050869 | negative regulation of B cell activation | 8/402 | 4.90E-07 | 6.77E-06 |
| BP | GO:0050921 | positive regulation of chemotaxis | 15/402 | 4.94E-07 | 6.80E-06 |
| BP | GO:0030099 | myeloid cell differentiation | 27/402 | 5.27E-07 | 7.21E-06 |
| BP | GO:0002456 | T cell mediated immunity | 14/402 | 5.33E-07 | 7.26E-06 |
| BP | GO:0014065 | phosphatidylinositol 3-kinase signaling | 15/402 | 5.41E-07 | 7.33E-06 |
| BP | GO:0002886 | regulation of myeloid leukocyte mediated immunity | 10/402 | 5.68E-07 | 7.66E-06 |
| BP | GO:0051235 | maintenance of location | 24/402 | 6.00E-07 | 8.05E-06 |
| BP | GO:0001774 | microglial cell activation | 9/402 | 6.16E-07 | 8.23E-06 |
| BP | GO:0052547 | regulation of peptidase activity | 27/402 | 6.34E-07 | 8.42E-06 |
| BP | GO:0002399 | MHC class II protein complex assembly | 6/402 | 6.52E-07 | 8.56E-06 |
| BP | GO:0002503 | peptide antigen assembly with MHC class II protein complex | 6/402 | 6.52E-07 | 8.56E-06 |
| BP | GO:0050920 | regulation of chemotaxis | 19/402 | 6.55E-07 | 8.56E-06 |
| BP | GO:1903557 | positive regulation of tumor necrosis factor superfamily cytokine production | 13/402 | 6.57E-07 | 8.56E-06 |
| BP | GO:0036230 | granulocyte activation | 9/402 | 7.44E-07 | 9.66E-06 |
| BP | GO:0002821 | positive regulation of adaptive immune response | 14/402 | 7.85E-07 | 1.01E-05 |
| BP | GO:0071347 | cellular response to interleukin-1 | 13/402 | 8.11E-07 | 1.04E-05 |
| BP | GO:0045621 | positive regulation of lymphocyte differentiation | 14/402 | 8.63E-07 | 1.10E-05 |
| BP | GO:0051209 | release of sequestered calcium ion into cytosol | 14/402 | 8.63E-07 | 1.10E-05 |
| BP | GO:0014066 | regulation of phosphatidylinositol 3-kinase signaling | 13/402 | 9.00E-07 | 1.14E-05 |
| BP | GO:0032623 | interleukin-2 production | 10/402 | 9.09E-07 | 1.14E-05 |
| BP | GO:0032663 | regulation of interleukin-2 production | 10/402 | 9.09E-07 | 1.14E-05 |
| BP | GO:0051283 | negative regulation of sequestering of calcium ion | 14/402 | 9.48E-07 | 1.18E-05 |
| BP | GO:0070304 | positive regulation of stress-activated protein kinase signaling cascade | 14/402 | 9.48E-07 | 1.18E-05 |
| BP | GO:0046330 | positive regulation of JNK cascade | 12/402 | 9.50E-07 | 1.18E-05 |
| BP | GO:0001911 | negative regulation of leukocyte mediated cytotoxicity | 7/402 | 9.60E-07 | 1.18E-05 |
| BP | GO:0008154 | actin polymerization or depolymerization | 17/402 | 9.75E-07 | 1.20E-05 |
| BP | GO:0032615 | interleukin-12 production | 10/402 | 1.06E-06 | 1.29E-05 |
| BP | GO:0032655 | regulation of interleukin-12 production | 10/402 | 1.06E-06 | 1.29E-05 |
| BP | GO:0051282 | regulation of sequestering of calcium ion | 14/402 | 1.14E-06 | 1.38E-05 |
| BP | GO:0050878 | regulation of body fluid levels | 24/402 | 1.21E-06 | 1.46E-05 |
| BP | GO:0002702 | positive regulation of production of molecular mediator of immune response | 14/402 | 1.25E-06 | 1.50E-05 |
| BP | GO:0045582 | positive regulation of T cell differentiation | 13/402 | 1.35E-06 | 1.61E-05 |
| BP | GO:0007015 | actin filament organization | 27/402 | 1.35E-06 | 1.61E-05 |
| BP | GO:0045730 | respiratory burst | 8/402 | 1.51E-06 | 1.79E-05 |
| BP | GO:0006968 | cellular defense response | 9/402 | 1.51E-06 | 1.79E-05 |
| BP | GO:0051208 | sequestering of calcium ion | 14/402 | 1.63E-06 | 1.91E-05 |
| BP | GO:0001912 | positive regulation of leukocyte mediated cytotoxicity | 10/402 | 1.63E-06 | 1.91E-05 |
| BP | GO:0014068 | positive regulation of phosphatidylinositol 3-kinase signaling | 11/402 | 1.66E-06 | 1.94E-05 |
| BP | GO:0008360 | regulation of cell shape | 14/402 | 1.78E-06 | 2.07E-05 |
| BP | GO:0010959 | regulation of metal ion transport | 26/402 | 1.90E-06 | 2.19E-05 |
| BP | GO:0045580 | regulation of T cell differentiation | 16/402 | 1.93E-06 | 2.22E-05 |
| BP | GO:0002283 | neutrophil activation involved in immune response | 6/402 | 2.09E-06 | 2.40E-05 |
| BP | GO:0031342 | negative regulation of cell killing | 7/402 | 2.15E-06 | 2.46E-05 |
| BP | GO:0002367 | cytokine production involved in immune response | 13/402 | 2.18E-06 | 2.48E-05 |
| BP | GO:0032941 | secretion by tissue | 8/402 | 2.26E-06 | 2.56E-05 |
| BP | GO:0070555 | response to interleukin-1 | 14/402 | 2.30E-06 | 2.60E-05 |
| BP | GO:0002573 | myeloid leukocyte differentiation | 18/402 | 2.32E-06 | 2.61E-05 |
| BP | GO:0032418 | lysosome localization | 11/402 | 2.38E-06 | 2.64E-05 |
| BP | GO:0033077 | T cell differentiation in thymus | 11/402 | 2.38E-06 | 2.64E-05 |
| BP | GO:1990849 | vacuolar localization | 11/402 | 2.38E-06 | 2.64E-05 |
| BP | GO:0030041 | actin filament polymerization | 15/402 | 2.43E-06 | 2.68E-05 |
| BP | GO:0043370 | regulation of CD4-positive, alpha-beta T cell differentiation | 9/402 | 2.47E-06 | 2.72E-05 |
| BP | GO:0033627 | cell adhesion mediated by integrin | 11/402 | 2.67E-06 | 2.93E-05 |
| BP | GO:0060142 | regulation of syncytium formation by plasma membrane fusion | 7/402 | 2.76E-06 | 3.00E-05 |
| BP | GO:0032640 | tumor necrosis factor production | 16/402 | 2.76E-06 | 3.00E-05 |
| BP | GO:0032680 | regulation of tumor necrosis factor production | 16/402 | 2.76E-06 | 3.00E-05 |
| BP | GO:0010720 | positive regulation of cell development | 26/402 | 2.79E-06 | 3.00E-05 |
| BP | GO:0032760 | positive regulation of tumor necrosis factor production | 12/402 | 2.79E-06 | 3.00E-05 |
| BP | GO:0002707 | negative regulation of lymphocyte mediated immunity | 9/402 | 2.89E-06 | 3.08E-05 |
| BP | GO:0070231 | T cell apoptotic process | 9/402 | 2.89E-06 | 3.08E-05 |
| BP | GO:0002396 | MHC protein complex assembly | 6/402 | 2.93E-06 | 3.10E-05 |
| BP | GO:0002501 | peptide antigen assembly with MHC protein complex | 6/402 | 2.93E-06 | 3.10E-05 |
| BP | GO:0043270 | positive regulation of monoatomic ion transport | 18/402 | 2.97E-06 | 3.14E-05 |
| BP | GO:0002832 | negative regulation of response to biotic stimulus | 13/402 | 3.15E-06 | 3.31E-05 |
| BP | GO:0002260 | lymphocyte homeostasis | 10/402 | 3.21E-06 | 3.35E-05 |
| BP | GO:0042130 | negative regulation of T cell proliferation | 10/402 | 3.21E-06 | 3.35E-05 |
| BP | GO:0002708 | positive regulation of lymphocyte mediated immunity | 13/402 | 3.44E-06 | 3.58E-05 |
| BP | GO:2000116 | regulation of cysteine-type endopeptidase activity | 18/402 | 3.57E-06 | 3.69E-05 |
| BP | GO:0042060 | wound healing | 26/402 | 3.73E-06 | 3.85E-05 |
| BP | GO:0046640 | regulation of alpha-beta T cell proliferation | 8/402 | 3.96E-06 | 4.06E-05 |
| BP | GO:0050856 | regulation of T cell receptor signaling pathway | 8/402 | 3.96E-06 | 4.06E-05 |
| BP | GO:1901739 | regulation of myoblast fusion | 6/402 | 4.03E-06 | 4.11E-05 |
| BP | GO:0031343 | positive regulation of cell killing | 10/402 | 4.14E-06 | 4.19E-05 |
| BP | GO:0045123 | cellular extravasation | 10/402 | 4.14E-06 | 4.19E-05 |
| BP | GO:0052548 | regulation of endopeptidase activity | 22/402 | 4.33E-06 | 4.37E-05 |
| BP | GO:0002431 | Fc receptor mediated stimulatory signaling pathway | 7/402 | 4.39E-06 | 4.41E-05 |
| BP | GO:0032874 | positive regulation of stress-activated MAPK cascade | 13/402 | 4.48E-06 | 4.49E-05 |
| BP | GO:0045061 | thymic T cell selection | 6/402 | 5.44E-06 | 5.41E-05 |
| BP | GO:0050858 | negative regulation of antigen receptor-mediated signaling pathway | 7/402 | 5.47E-06 | 5.41E-05 |
| BP | GO:0007259 | receptor signaling pathway via JAK-STAT | 15/402 | 5.48E-06 | 5.41E-05 |
| BP | GO:0031214 | biomineral tissue development | 15/402 | 5.48E-06 | 5.41E-05 |
| BP | GO:0070302 | regulation of stress-activated protein kinase signaling cascade | 16/402 | 5.83E-06 | 5.74E-05 |
| BP | GO:0002861 | regulation of inflammatory response to antigenic stimulus | 8/402 | 6.64E-06 | 6.49E-05 |
| BP | GO:0046633 | alpha-beta T cell proliferation | 8/402 | 6.64E-06 | 6.49E-05 |
| BP | GO:0002437 | inflammatory response to antigenic stimulus | 10/402 | 6.71E-06 | 6.53E-05 |
| BP | GO:0032743 | positive regulation of interleukin-2 production | 7/402 | 6.76E-06 | 6.53E-05 |
| BP | GO:0045577 | regulation of B cell differentiation | 7/402 | 6.76E-06 | 6.53E-05 |
| BP | GO:0031098 | stress-activated protein kinase signaling cascade | 18/402 | 6.78E-06 | 6.53E-05 |
| BP | GO:0043300 | regulation of leukocyte degranulation | 8/402 | 7.82E-06 | 7.49E-05 |
| BP | GO:0002709 | regulation of T cell mediated immunity | 11/402 | 7.83E-06 | 7.49E-05 |
| BP | GO:0097553 | calcium ion transmembrane import into cytosol | 16/402 | 8.04E-06 | 7.66E-05 |
| BP | GO:0002292 | T cell differentiation involved in immune response | 10/402 | 8.45E-06 | 8.03E-05 |
| BP | GO:0070233 | negative regulation of T cell apoptotic process | 6/402 | 9.46E-06 | 8.96E-05 |
| BP | GO:0002833 | positive regulation of response to biotic stimulus | 21/402 | 9.61E-06 | 9.07E-05 |
| BP | GO:0046328 | regulation of JNK cascade | 13/402 | 1.02E-05 | 9.59E-05 |
| BP | GO:0031663 | lipopolysaccharide-mediated signaling pathway | 9/402 | 1.03E-05 | 9.61E-05 |
| BP | GO:0043254 | regulation of protein-containing complex assembly | 24/402 | 1.03E-05 | 9.64E-05 |
| BP | GO:0002718 | regulation of cytokine production involved in immune response | 12/402 | 1.04E-05 | 9.64E-05 |
| BP | GO:0050848 | regulation of calcium-mediated signaling | 10/402 | 1.06E-05 | 9.81E-05 |
| BP | GO:0001914 | regulation of T cell mediated cytotoxicity | 8/402 | 1.07E-05 | 9.91E-05 |
| BP | GO:0097696 | receptor signaling pathway via STAT | 15/402 | 1.16E-05 | 0.000106373 |
| BP | GO:0050764 | regulation of phagocytosis | 11/402 | 1.16E-05 | 0.000106373 |
| BP | GO:0046641 | positive regulation of alpha-beta T cell proliferation | 6/402 | 1.22E-05 | 0.000111509 |
| BP | GO:0060333 | type II interferon-mediated signaling pathway | 6/402 | 1.22E-05 | 0.000111509 |
| BP | GO:0045058 | T cell selection | 8/402 | 1.25E-05 | 0.000113407 |
| BP | GO:0019722 | calcium-mediated signaling | 15/402 | 1.40E-05 | 0.000127293 |
| BP | GO:0002715 | regulation of natural killer cell mediated immunity | 8/402 | 1.45E-05 | 0.000130241 |
| BP | GO:0046638 | positive regulation of alpha-beta T cell differentiation | 8/402 | 1.45E-05 | 0.000130241 |
| BP | GO:0002335 | mature B cell differentiation | 7/402 | 1.47E-05 | 0.000132144 |
| BP | GO:2000401 | regulation of lymphocyte migration | 9/402 | 1.49E-05 | 0.000133631 |
| BP | GO:0046629 | gamma-delta T cell activation | 6/402 | 1.56E-05 | 0.000139121 |
| BP | GO:0002377 | immunoglobulin production | 16/402 | 1.57E-05 | 0.000139589 |
| BP | GO:0051403 | stress-activated MAPK cascade | 17/402 | 1.66E-05 | 0.000147424 |
| BP | GO:0042093 | T-helper cell differentiation | 9/402 | 1.69E-05 | 0.000148856 |
| BP | GO:0001580 | detection of chemical stimulus involved in sensory perception of bitter taste | 7/402 | 1.76E-05 | 0.000154572 |
| BP | GO:0070232 | regulation of T cell apoptotic process | 7/402 | 1.76E-05 | 0.000154572 |
| BP | GO:0009612 | response to mechanical stimulus | 16/402 | 1.87E-05 | 0.000163552 |
| BP | GO:0042832 | defense response to protozoan | 6/402 | 1.97E-05 | 0.000171752 |
| BP | GO:0007254 | JNK cascade | 14/402 | 2.02E-05 | 0.000175164 |
| BP | GO:0032872 | regulation of stress-activated MAPK cascade | 15/402 | 2.04E-05 | 0.00017706 |
| BP | GO:0019932 | second-messenger-mediated signaling | 19/402 | 2.12E-05 | 0.000183437 |
| BP | GO:0002294 | CD4-positive, alpha-beta T cell differentiation involved in immune response | 9/402 | 2.13E-05 | 0.000183628 |
| BP | GO:0032956 | regulation of actin cytoskeleton organization | 21/402 | 2.24E-05 | 0.000192678 |
| BP | GO:0030889 | negative regulation of B cell proliferation | 5/402 | 2.29E-05 | 0.000195886 |
| BP | GO:0002287 | alpha-beta T cell activation involved in immune response | 9/402 | 2.39E-05 | 0.000203377 |
| BP | GO:0002293 | alpha-beta T cell differentiation involved in immune response | 9/402 | 2.39E-05 | 0.000203377 |
| BP | GO:2000403 | positive regulation of lymphocyte migration | 7/402 | 2.48E-05 | 0.000210345 |
| BP | GO:0046637 | regulation of alpha-beta T cell differentiation | 9/402 | 2.68E-05 | 0.000225532 |
| BP | GO:0050766 | positive regulation of phagocytosis | 9/402 | 2.68E-05 | 0.000225532 |
| BP | GO:0010810 | regulation of cell-substrate adhesion | 16/402 | 2.77E-05 | 0.000232962 |
| BP | GO:0001562 | response to protozoan | 6/402 | 3.05E-05 | 0.00025449 |
| BP | GO:0090025 | regulation of monocyte chemotaxis | 6/402 | 3.05E-05 | 0.00025449 |
| BP | GO:0045089 | positive regulation of innate immune response | 19/402 | 3.08E-05 | 0.000256599 |
| BP | GO:0007229 | integrin-mediated signaling pathway | 11/402 | 3.09E-05 | 0.000256874 |
| BP | GO:0055074 | calcium ion homeostasis | 20/402 | 3.15E-05 | 0.00025978 |
| BP | GO:0071496 | cellular response to external stimulus | 20/402 | 3.15E-05 | 0.00025978 |
| BP | GO:0002381 | immunoglobulin production involved in immunoglobulin-mediated immune response | 9/402 | 3.33E-05 | 0.000274213 |
| BP | GO:0042554 | superoxide anion generation | 7/402 | 3.43E-05 | 0.000281018 |
| BP | GO:0007584 | response to nutrient | 13/402 | 3.50E-05 | 0.000286611 |
| BP | GO:0007204 | positive regulation of cytosolic calcium ion concentration | 14/402 | 3.58E-05 | 0.000292101 |
| BP | GO:0002637 | regulation of immunoglobulin production | 9/402 | 3.71E-05 | 0.00030161 |
| BP | GO:0002507 | tolerance induction | 6/402 | 3.74E-05 | 0.000303198 |
| BP | GO:0032970 | regulation of actin filament-based process | 22/402 | 3.89E-05 | 0.000314768 |
| BP | GO:0033003 | regulation of mast cell activation | 7/402 | 4.00E-05 | 0.000321362 |
| BP | GO:0050913 | sensory perception of bitter taste | 7/402 | 4.00E-05 | 0.000321362 |
| BP | GO:0001915 | negative regulation of T cell mediated cytotoxicity | 4/402 | 4.06E-05 | 0.000323411 |
| BP | GO:0002291 | T cell activation via T cell receptor contact with antigen bound to MHC molecule on antigen presenting cell | 4/402 | 4.06E-05 | 0.000323411 |
| BP | GO:0035747 | natural killer cell chemotaxis | 4/402 | 4.06E-05 | 0.000323411 |
| BP | GO:0050912 | detection of chemical stimulus involved in sensory perception of taste | 7/402 | 4.65E-05 | 0.000368211 |
| BP | GO:0097028 | dendritic cell differentiation | 7/402 | 4.65E-05 | 0.000368211 |
| BP | GO:0002643 | regulation of tolerance induction | 5/402 | 5.44E-05 | 0.000429347 |
| BP | GO:0043552 | positive regulation of phosphatidylinositol 3-kinase activity | 6/402 | 5.50E-05 | 0.000433434 |
| BP | GO:0002720 | positive regulation of cytokine production involved in immune response | 9/402 | 5.60E-05 | 0.000439558 |
| BP | GO:0030198 | extracellular matrix organization | 19/402 | 5.72E-05 | 0.000447628 |
| BP | GO:0050728 | negative regulation of inflammatory response | 14/402 | 5.79E-05 | 0.00045174 |
| BP | GO:0018108 | peptidyl-tyrosine phosphorylation | 21/402 | 5.96E-05 | 0.000463152 |
| BP | GO:0043062 | extracellular structure organization | 19/402 | 5.96E-05 | 0.000463152 |
| BP | GO:0032731 | positive regulation of interleukin-1 beta production | 8/402 | 6.05E-05 | 0.000468061 |
| BP | GO:0140888 | interferon-mediated signaling pathway | 10/402 | 6.06E-05 | 0.000468061 |
| BP | GO:0045060 | negative thymic T cell selection | 4/402 | 6.27E-05 | 0.00048286 |
| BP | GO:0018212 | peptidyl-tyrosine modification | 21/402 | 6.43E-05 | 0.000494066 |
| BP | GO:0045229 | external encapsulating structure organization | 19/402 | 6.49E-05 | 0.000497011 |
| BP | GO:0033006 | regulation of mast cell activation involved in immune response | 6/402 | 6.61E-05 | 0.000504372 |
| BP | GO:0045637 | regulation of myeloid cell differentiation | 15/402 | 6.75E-05 | 0.000514241 |
| BP | GO:0002716 | negative regulation of natural killer cell mediated immunity | 5/402 | 7.01E-05 | 0.000532281 |
| BP | GO:0002673 | regulation of acute inflammatory response | 7/402 | 7.13E-05 | 0.000537377 |
| BP | GO:0042269 | regulation of natural killer cell mediated cytotoxicity | 7/402 | 7.13E-05 | 0.000537377 |
| BP | GO:2000404 | regulation of T cell migration | 7/402 | 7.13E-05 | 0.000537377 |
| BP | GO:0002532 | production of molecular mediator involved in inflammatory response | 10/402 | 7.18E-05 | 0.000539314 |
| BP | GO:0042886 | amide transport | 20/402 | 7.80E-05 | 0.000582724 |
| BP | GO:0045765 | regulation of angiogenesis | 20/402 | 7.80E-05 | 0.000582724 |
| BP | GO:0110053 | regulation of actin filament organization | 17/402 | 7.85E-05 | 0.000583654 |
| BP | GO:0090322 | regulation of superoxide metabolic process | 6/402 | 7.88E-05 | 0.000583654 |
| BP | GO:2000406 | positive regulation of T cell migration | 6/402 | 7.88E-05 | 0.000583654 |
| BP | GO:0033628 | regulation of cell adhesion mediated by integrin | 7/402 | 8.17E-05 | 0.000603906 |
| BP | GO:0009615 | response to virus | 22/402 | 8.50E-05 | 0.000626389 |
| BP | GO:0032928 | regulation of superoxide anion generation | 5/402 | 8.91E-05 | 0.000655087 |
| BP | GO:0022604 | regulation of cell morphogenesis | 16/402 | 9.03E-05 | 0.000661972 |
| BP | GO:0043383 | negative T cell selection | 4/402 | 9.25E-05 | 0.000672613 |
| BP | GO:0097048 | dendritic cell apoptotic process | 4/402 | 9.25E-05 | 0.000672613 |
| BP | GO:2000668 | regulation of dendritic cell apoptotic process | 4/402 | 9.25E-05 | 0.000672613 |
| BP | GO:0032633 | interleukin-4 production | 6/402 | 9.33E-05 | 0.000675393 |
| BP | GO:0032673 | regulation of interleukin-4 production | 6/402 | 9.33E-05 | 0.000675393 |
| BP | GO:0050909 | sensory perception of taste | 8/402 | 9.46E-05 | 0.000682542 |
| BP | GO:0008064 | regulation of actin polymerization or depolymerization | 12/402 | 9.69E-05 | 0.000697761 |
| BP | GO:1901342 | regulation of vasculature development | 20/402 | 9.84E-05 | 0.000706549 |
| BP | GO:0090218 | positive regulation of lipid kinase activity | 6/402 | 0.000109981 | 0.000787596 |
| BP | GO:0050855 | regulation of B cell receptor signaling pathway | 5/402 | 0.000111853 | 0.000798925 |
| BP | GO:0051258 | protein polymerization | 17/402 | 0.000112383 | 0.000799461 |
| BP | GO:0006874 | intracellular calcium ion homeostasis | 18/402 | 0.000112572 | 0.000799461 |
| BP | GO:0007596 | blood coagulation | 15/402 | 0.000112795 | 0.000799461 |
| BP | GO:0051928 | positive regulation of calcium ion transport | 11/402 | 0.000113154 | 0.000799952 |
| BP | GO:0032963 | collagen metabolic process | 10/402 | 0.000116401 | 0.000820807 |
| BP | GO:0002711 | positive regulation of T cell mediated immunity | 8/402 | 0.000116773 | 0.000821337 |
| BP | GO:0030832 | regulation of actin filament length | 12/402 | 0.00011728 | 0.000822807 |
| BP | GO:0030833 | regulation of actin filament polymerization | 11/402 | 0.000121264 | 0.000846462 |
| BP | GO:1903305 | regulation of regulated secretory pathway | 11/402 | 0.000121264 | 0.000846462 |
| BP | GO:0033209 | tumor necrosis factor-mediated signaling pathway | 10/402 | 0.000125737 | 0.000875476 |
| BP | GO:0002664 | regulation of T cell tolerance induction | 4/402 | 0.000131288 | 0.00090727 |
| BP | GO:0030157 | pancreatic juice secretion | 4/402 | 0.000131288 | 0.00090727 |
| BP | GO:0046541 | saliva secretion | 4/402 | 0.000131288 | 0.00090727 |
| BP | GO:0043588 | skin development | 18/402 | 0.000138195 | 0.00095262 |
| BP | GO:0060143 | positive regulation of syncytium formation by plasma membrane fusion | 5/402 | 0.000138798 | 0.000954393 |
| BP | GO:0050817 | coagulation | 15/402 | 0.000144023 | 0.000987866 |
| BP | GO:0030168 | platelet activation | 11/402 | 0.000148629 | 0.001016938 |
| BP | GO:0043368 | positive T cell selection | 6/402 | 0.000150308 | 0.001023355 |
| BP | GO:0070229 | negative regulation of lymphocyte apoptotic process | 6/402 | 0.000150308 | 0.001023355 |
| BP | GO:0007599 | hemostasis | 15/402 | 0.000151099 | 0.001026212 |
| BP | GO:2000117 | negative regulation of cysteine-type endopeptidase activity | 9/402 | 0.000153599 | 0.00104064 |
| BP | GO:0042476 | odontogenesis | 11/402 | 0.000169652 | 0.001146583 |
| BP | GO:0002483 | antigen processing and presentation of endogenous peptide antigen | 5/402 | 0.000170441 | 0.001149111 |
| BP | GO:0015833 | peptide transport | 16/402 | 0.000171777 | 0.001155297 |
| BP | GO:0031529 | ruffle organization | 7/402 | 0.000172729 | 0.001158882 |
| BP | GO:0071260 | cellular response to mechanical stimulus | 8/402 | 0.000174169 | 0.001164432 |
| BP | GO:0001503 | ossification | 22/402 | 0.000174399 | 0.001164432 |
| BP | GO:1901654 | response to ketone | 14/402 | 0.000179689 | 0.001196864 |
| BP | GO:0033674 | positive regulation of kinase activity | 22/402 | 0.000180216 | 0.00119749 |
| BP | GO:0045059 | positive thymic T cell selection | 4/402 | 0.000180678 | 0.001197681 |
| BP | GO:0010951 | negative regulation of endopeptidase activity | 12/402 | 0.00019032 | 0.001258387 |
| BP | GO:0060348 | bone development | 15/402 | 0.000191192 | 0.001258387 |
| BP | GO:0006801 | superoxide metabolic process | 8/402 | 0.000191657 | 0.001258387 |
| BP | GO:0032732 | positive regulation of interleukin-1 production | 8/402 | 0.000191657 | 0.001258387 |
| BP | GO:0002823 | negative regulation of adaptive immune response based on somatic recombination of immune receptors built from immunoglobulin superfamily domains | 7/402 | 0.000193787 | 0.001269355 |
| BP | GO:0051346 | negative regulation of hydrolase activity | 19/402 | 0.000195712 | 0.001278933 |
| BP | GO:0010466 | negative regulation of peptidase activity | 15/402 | 0.000200231 | 0.001305382 |
| BP | GO:1904062 | regulation of monoatomic cation transmembrane transport | 19/402 | 0.000203058 | 0.001320694 |
| BP | GO:0002433 | immune response-regulating cell surface receptor signaling pathway involved in phagocytosis | 5/402 | 0.000207308 | 0.001332656 |
| BP | GO:0033622 | integrin activation | 5/402 | 0.000207308 | 0.001332656 |
| BP | GO:0038096 | Fc-gamma receptor signaling pathway involved in phagocytosis | 5/402 | 0.000207308 | 0.001332656 |
| BP | GO:0050857 | positive regulation of antigen receptor-mediated signaling pathway | 5/402 | 0.000207308 | 0.001332656 |
| BP | GO:1901623 | regulation of lymphocyte chemotaxis | 5/402 | 0.000207308 | 0.001332656 |
| BP | GO:0072593 | reactive oxygen species metabolic process | 15/402 | 0.000209639 | 0.001343327 |
| BP | GO:0043405 | regulation of MAP kinase activity | 13/402 | 0.00020994 | 0.001343327 |
| BP | GO:1902903 | regulation of supramolecular fiber organization | 20/402 | 0.000212001 | 0.001353385 |
| BP | GO:0001667 | ameboidal-type cell migration | 24/402 | 0.000214286 | 0.001364822 |
| BP | GO:0042063 | gliogenesis | 18/402 | 0.000221576 | 0.001408009 |
| BP | GO:0150077 | regulation of neuroinflammatory response | 6/402 | 0.000231811 | 0.001469666 |
| BP | GO:2001235 | positive regulation of apoptotic signaling pathway | 11/402 | 0.000233524 | 0.001477136 |
| BP | GO:0051702 | biological process involved in interaction with symbiont | 10/402 | 0.000242115 | 0.001507823 |
| BP | GO:0002468 | dendritic cell antigen processing and presentation | 4/402 | 0.000242193 | 0.001507823 |
| BP | GO:0002517 | T cell tolerance induction | 4/402 | 0.000242193 | 0.001507823 |
| BP | GO:0002679 | respiratory burst involved in defense response | 4/402 | 0.000242193 | 0.001507823 |
| BP | GO:0002864 | regulation of acute inflammatory response to antigenic stimulus | 4/402 | 0.000242193 | 0.001507823 |
| BP | GO:0098883 | synapse pruning | 4/402 | 0.000242193 | 0.001507823 |
| BP | GO:1902563 | regulation of neutrophil activation | 4/402 | 0.000242193 | 0.001507823 |
| BP | GO:0032740 | positive regulation of interleukin-17 production | 5/402 | 0.000249947 | 0.001549115 |
| BP | GO:0032753 | positive regulation of interleukin-4 production | 5/402 | 0.000249947 | 0.001549115 |
| BP | GO:0030278 | regulation of ossification | 10/402 | 0.000259342 | 0.001603748 |
| BP | GO:0043551 | regulation of phosphatidylinositol 3-kinase activity | 6/402 | 0.000265576 | 0.001634984 |
| BP | GO:0045622 | regulation of T-helper cell differentiation | 6/402 | 0.000265576 | 0.001634984 |
| BP | GO:0030856 | regulation of epithelial cell differentiation | 12/402 | 0.000267886 | 0.001645543 |
| BP | GO:0032535 | regulation of cellular component size | 19/402 | 0.000271067 | 0.001661388 |
| BP | GO:0030282 | bone mineralization | 10/402 | 0.000296886 | 0.00181561 |
| BP | GO:0002675 | positive regulation of acute inflammatory response | 5/402 | 0.000298932 | 0.001824085 |
| BP | GO:0032735 | positive regulation of interleukin-12 production | 6/402 | 0.000303087 | 0.001845364 |
| BP | GO:0034765 | regulation of monoatomic ion transmembrane transport | 23/402 | 0.000312834 | 0.001900524 |
| BP | GO:0050862 | positive regulation of T cell receptor signaling pathway | 4/402 | 0.000317442 | 0.001915886 |
| BP | GO:1901741 | positive regulation of myoblast fusion | 4/402 | 0.000317442 | 0.001915886 |
| BP | GO:2000696 | regulation of epithelial cell differentiation involved in kidney development | 4/402 | 0.000317442 | 0.001915886 |
| BP | GO:0002820 | negative regulation of adaptive immune response | 7/402 | 0.000332369 | 0.001997257 |
| BP | GO:0034113 | heterotypic cell-cell adhesion | 7/402 | 0.000332369 | 0.001997257 |
| BP | GO:0043029 | T cell homeostasis | 6/402 | 0.000344628 | 0.002066429 |
| BP | GO:0000768 | syncytium formation by plasma membrane fusion | 7/402 | 0.000367817 | 0.002186504 |
| BP | GO:0022617 | extracellular matrix disassembly | 7/402 | 0.000367817 | 0.002186504 |
| BP | GO:0043030 | regulation of macrophage activation | 7/402 | 0.000367817 | 0.002186504 |
| BP | GO:0140253 | cell-cell fusion | 7/402 | 0.000367817 | 0.002186504 |
| BP | GO:0001952 | regulation of cell-matrix adhesion | 10/402 | 0.00038569 | 0.002287827 |
| BP | GO:0051279 | regulation of release of sequestered calcium ion into cytosol | 8/402 | 0.000390236 | 0.002296619 |
| BP | GO:0030574 | collagen catabolic process | 6/402 | 0.000390495 | 0.002296619 |
| BP | GO:0030890 | positive regulation of B cell proliferation | 6/402 | 0.000390495 | 0.002296619 |
| BP | GO:1902622 | regulation of neutrophil migration | 6/402 | 0.000390495 | 0.002296619 |
| BP | GO:0033631 | cell-cell adhesion mediated by integrin | 4/402 | 0.000408073 | 0.002389832 |
| BP | GO:2001267 | regulation of cysteine-type endopeptidase activity involved in apoptotic signaling pathway | 4/402 | 0.000408073 | 0.002389832 |
| BP | GO:0045860 | positive regulation of protein kinase activity | 19/402 | 0.000410251 | 0.002397092 |
| BP | GO:0019730 | antimicrobial humoral response | 10/402 | 0.000411048 | 0.002397092 |
| BP | GO:0002825 | regulation of T-helper 1 type immune response | 5/402 | 0.000418352 | 0.002434554 |
| BP | GO:2000377 | regulation of reactive oxygen species metabolic process | 11/402 | 0.000423379 | 0.0024563 |
| BP | GO:0050871 | positive regulation of B cell activation | 8/402 | 0.000423866 | 0.0024563 |
| BP | GO:0034767 | positive regulation of monoatomic ion transmembrane transport | 12/402 | 0.000433716 | 0.002508124 |
| BP | GO:0002455 | humoral immune response mediated by circulating immunoglobulin | 6/402 | 0.000440996 | 0.002544895 |
| BP | GO:0050730 | regulation of peptidyl-tyrosine phosphorylation | 15/402 | 0.000457519 | 0.002634748 |
| BP | GO:0090066 | regulation of anatomical structure size | 23/402 | 0.000468385 | 0.002691714 |
| BP | GO:0043304 | regulation of mast cell degranulation | 5/402 | 0.000490047 | 0.002810356 |
| BP | GO:0044403 | biological process involved in symbiotic interaction | 17/402 | 0.000491519 | 0.002812967 |
| BP | GO:0002701 | negative regulation of production of molecular mediator of immune response | 6/402 | 0.000496446 | 0.002829448 |
| BP | GO:0002920 | regulation of humoral immune response | 6/402 | 0.000496446 | 0.002829448 |
| BP | GO:0002830 | positive regulation of type 2 immune response | 4/402 | 0.000515771 | 0.002915539 |
| BP | GO:0032604 | granulocyte macrophage colony-stimulating factor production | 4/402 | 0.000515771 | 0.002915539 |
| BP | GO:0032645 | regulation of granulocyte macrophage colony-stimulating factor production | 4/402 | 0.000515771 | 0.002915539 |
| BP | GO:0051770 | positive regulation of nitric-oxide synthase biosynthetic process | 4/402 | 0.000515771 | 0.002915539 |
| BP | GO:0022600 | digestive system process | 9/402 | 0.000519403 | 0.002930082 |
| BP | GO:0007260 | tyrosine phosphorylation of STAT protein | 8/402 | 0.00053913 | 0.003035172 |
| BP | GO:0006949 | syncytium formation | 7/402 | 0.000541032 | 0.003039685 |
| BP | GO:0006953 | acute-phase response | 6/402 | 0.000557175 | 0.003111412 |
| BP | GO:0007520 | myoblast fusion | 6/402 | 0.000557175 | 0.003111412 |
| BP | GO:0035987 | endodermal cell differentiation | 6/402 | 0.000557175 | 0.003111412 |
| BP | GO:0051047 | positive regulation of secretion | 17/402 | 0.000567332 | 0.003148241 |
| BP | GO:0019883 | antigen processing and presentation of endogenous antigen | 5/402 | 0.000570604 | 0.003148241 |
| BP | GO:0032770 | positive regulation of monooxygenase activity | 5/402 | 0.000570604 | 0.003148241 |
| BP | GO:0034694 | response to prostaglandin | 5/402 | 0.000570604 | 0.003148241 |
| BP | GO:0071295 | cellular response to vitamin | 5/402 | 0.000570604 | 0.003148241 |
| BP | GO:0071353 | cellular response to interleukin-4 | 5/402 | 0.000570604 | 0.003148241 |
| BP | GO:0048771 | tissue remodeling | 12/402 | 0.000586789 | 0.003224668 |
| BP | GO:0050731 | positive regulation of peptidyl-tyrosine phosphorylation | 12/402 | 0.000586789 | 0.003224668 |
| BP | GO:0042531 | positive regulation of tyrosine phosphorylation of STAT protein | 7/402 | 0.000593119 | 0.003252986 |
| BP | GO:0006911 | phagocytosis, engulfment | 6/402 | 0.000623519 | 0.003412946 |
| BP | GO:0033273 | response to vitamin | 8/402 | 0.000629169 | 0.003437068 |
| BP | GO:0032695 | negative regulation of interleukin-12 production | 4/402 | 0.000642241 | 0.0034878 |
| BP | GO:0032930 | positive regulation of superoxide anion generation | 4/402 | 0.000642241 | 0.0034878 |
| BP | GO:0043031 | negative regulation of macrophage activation | 4/402 | 0.000642241 | 0.0034878 |
| BP | GO:0072677 | eosinophil migration | 5/402 | 0.000660702 | 0.00358102 |
| BP | GO:0007589 | body fluid secretion | 8/402 | 0.000678532 | 0.003665024 |
| BP | GO:0051341 | regulation of oxidoreductase activity | 9/402 | 0.000678853 | 0.003665024 |
| BP | GO:0032611 | interleukin-1 beta production | 9/402 | 0.000724397 | 0.003895691 |
| BP | GO:0032651 | regulation of interleukin-1 beta production | 9/402 | 0.000724397 | 0.003895691 |
| BP | GO:1903169 | regulation of calcium ion transmembrane transport | 12/402 | 0.000746848 | 0.004008632 |
| BP | GO:0001782 | B cell homeostasis | 5/402 | 0.000761037 | 0.00406899 |
| BP | GO:0044546 | NLRP3 inflammasome complex assembly | 5/402 | 0.000761037 | 0.00406899 |
| BP | GO:0007200 | phospholipase C-activating G protein-coupled receptor signaling pathway | 9/402 | 0.000772402 | 0.004121781 |
| BP | GO:0045953 | negative regulation of natural killer cell mediated cytotoxicity | 4/402 | 0.000789211 | 0.004195279 |
| BP | GO:0072234 | metanephric nephron tubule development | 4/402 | 0.000789211 | 0.004195279 |
| BP | GO:0031532 | actin cytoskeleton reorganization | 9/402 | 0.000822968 | 0.004366327 |
| BP | GO:0002446 | neutrophil mediated immunity | 5/402 | 0.000872321 | 0.004601678 |
| BP | GO:0060416 | response to growth hormone | 5/402 | 0.000872321 | 0.004601678 |
| BP | GO:0070670 | response to interleukin-4 | 5/402 | 0.000872321 | 0.004601678 |
| BP | GO:0016042 | lipid catabolic process | 17/402 | 0.000887114 | 0.004657116 |
| BP | GO:0045861 | negative regulation of proteolysis | 17/402 | 0.000887114 | 0.004657116 |
| BP | GO:0007586 | digestion | 10/402 | 0.000887885 | 0.004657116 |
| BP | GO:0070588 | calcium ion transmembrane transport | 18/402 | 0.000891586 | 0.004667671 |
| BP | GO:0032271 | regulation of protein polymerization | 12/402 | 0.000941906 | 0.004921791 |
| BP | GO:0002639 | positive regulation of immunoglobulin production | 6/402 | 0.000952145 | 0.00495189 |
| BP | GO:0071622 | regulation of granulocyte chemotaxis | 6/402 | 0.000952145 | 0.00495189 |
| BP | GO:0002888 | positive regulation of myeloid leukocyte mediated immunity | 4/402 | 0.000958415 | 0.00495189 |
| BP | GO:0030502 | negative regulation of bone mineralization | 4/402 | 0.000958415 | 0.00495189 |
| BP | GO:0050849 | negative regulation of calcium-mediated signaling | 4/402 | 0.000958415 | 0.00495189 |
| BP | GO:0090026 | positive regulation of monocyte chemotaxis | 4/402 | 0.000958415 | 0.00495189 |
| BP | GO:0051607 | defense response to virus | 16/402 | 0.000961883 | 0.004960536 |
| BP | GO:0043406 | positive regulation of MAP kinase activity | 9/402 | 0.000991046 | 0.005087771 |
| BP | GO:0050673 | epithelial cell proliferation | 22/402 | 0.000994378 | 0.005087771 |
| BP | GO:0002474 | antigen processing and presentation of peptide antigen via MHC class I | 5/402 | 0.000995279 | 0.005087771 |
| BP | GO:0140632 | inflammasome complex assembly | 5/402 | 0.000995279 | 0.005087771 |
| BP | GO:0140546 | defense response to symbiont | 16/402 | 0.000995758 | 0.005087771 |
| BP | GO:0043550 | regulation of lipid kinase activity | 6/402 | 0.001051968 | 0.005359842 |
| BP | GO:0051897 | positive regulation of protein kinase B signaling | 9/402 | 0.001052885 | 0.005359842 |
| BP | GO:0002361 | CD4-positive, CD25-positive, alpha-beta regulatory T cell differentiation | 3/402 | 0.001071619 | 0.005415323 |
| BP | GO:0045625 | regulation of T-helper 1 cell differentiation | 3/402 | 0.001071619 | 0.005415323 |
| BP | GO:0060054 | positive regulation of epithelial cell proliferation involved in wound healing | 3/402 | 0.001071619 | 0.005415323 |
| BP | GO:0060368 | regulation of Fc receptor mediated stimulatory signaling pathway | 3/402 | 0.001071619 | 0.005415323 |
| BP | GO:0021782 | glial cell development | 9/402 | 0.001117811 | 0.005638438 |
| BP | GO:0033280 | response to vitamin D | 5/402 | 0.001130651 | 0.005692818 |
| BP | GO:0035739 | CD4-positive, alpha-beta T cell proliferation | 4/402 | 0.001151595 | 0.005745939 |
| BP | GO:0045624 | positive regulation of T-helper cell differentiation | 4/402 | 0.001151595 | 0.005745939 |
| BP | GO:0051767 | nitric-oxide synthase biosynthetic process | 4/402 | 0.001151595 | 0.005745939 |
| BP | GO:0051769 | regulation of nitric-oxide synthase biosynthetic process | 4/402 | 0.001151595 | 0.005745939 |
| BP | GO:2000561 | regulation of CD4-positive, alpha-beta T cell proliferation | 4/402 | 0.001151595 | 0.005745939 |
| BP | GO:0002686 | negative regulation of leukocyte migration | 6/402 | 0.00115963 | 0.005775604 |
| BP | GO:0043200 | response to amino acid | 9/402 | 0.001185937 | 0.005896008 |
| BP | GO:0034109 | homotypic cell-cell adhesion | 8/402 | 0.001196193 | 0.005936318 |
| BP | GO:0001822 | kidney development | 16/402 | 0.001221 | 0.006048569 |
| BP | GO:0002520 | immune system development | 12/402 | 0.001230244 | 0.006083457 |
| BP | GO:0072073 | kidney epithelium development | 10/402 | 0.001291732 | 0.006376107 |
| BP | GO:0032602 | chemokine production | 8/402 | 0.001365264 | 0.006704985 |
| BP | GO:0019835 | cytolysis | 4/402 | 0.001370488 | 0.006704985 |
| BP | GO:0019885 | antigen processing and presentation of endogenous peptide antigen via MHC class I | 4/402 | 0.001370488 | 0.006704985 |
| BP | GO:0072170 | metanephric tubule development | 4/402 | 0.001370488 | 0.006704985 |
| BP | GO:0072243 | metanephric nephron epithelium development | 4/402 | 0.001370488 | 0.006704985 |
| BP | GO:0001706 | endoderm formation | 6/402 | 0.001400079 | 0.006837656 |
| BP | GO:0002761 | regulation of myeloid leukocyte differentiation | 9/402 | 0.001410678 | 0.006877267 |
| BP | GO:0030279 | negative regulation of ossification | 5/402 | 0.001441651 | 0.007006984 |
| BP | GO:0002604 | regulation of dendritic cell antigen processing and presentation | 3/402 | 0.00144996 | 0.007006984 |
| BP | GO:0033089 | positive regulation of T cell differentiation in thymus | 3/402 | 0.00144996 | 0.007006984 |
| BP | GO:0033632 | regulation of cell-cell adhesion mediated by integrin | 3/402 | 0.00144996 | 0.007006984 |
| BP | GO:0045628 | regulation of T-helper 2 cell differentiation | 3/402 | 0.00144996 | 0.007006984 |
| BP | GO:0050709 | negative regulation of protein secretion | 7/402 | 0.001471773 | 0.007099984 |
| BP | GO:0099024 | plasma membrane invagination | 6/402 | 0.00153369 | 0.007385787 |
| BP | GO:0002922 | positive regulation of humoral immune response | 4/402 | 0.001616826 | 0.007701792 |
| BP | GO:0034695 | response to prostaglandin E | 4/402 | 0.001616826 | 0.007701792 |
| BP | GO:0051043 | regulation of membrane protein ectodomain proteolysis | 4/402 | 0.001616826 | 0.007701792 |
| BP | GO:0071676 | negative regulation of mononuclear cell migration | 4/402 | 0.001616826 | 0.007701792 |
| BP | GO:0001916 | positive regulation of T cell mediated cytotoxicity | 5/402 | 0.001618813 | 0.007701792 |
| BP | GO:0010863 | positive regulation of phospholipase C activity | 5/402 | 0.001618813 | 0.007701792 |
| BP | GO:0050850 | positive regulation of calcium-mediated signaling | 5/402 | 0.001618813 | 0.007701792 |
| BP | GO:0030316 | osteoclast differentiation | 8/402 | 0.001653918 | 0.00785529 |
| BP | GO:0072001 | renal system development | 16/402 | 0.001691813 | 0.008020208 |
| BP | GO:0031668 | cellular response to extracellular stimulus | 14/402 | 0.001694445 | 0.008020208 |
| BP | GO:0001895 | retina homeostasis | 7/402 | 0.001705929 | 0.0080333 |
| BP | GO:0030500 | regulation of bone mineralization | 7/402 | 0.001705929 | 0.0080333 |
| BP | GO:0071230 | cellular response to amino acid stimulus | 7/402 | 0.001705929 | 0.0080333 |
| BP | GO:0002218 | activation of innate immune response | 13/402 | 0.001732226 | 0.008143262 |
| BP | GO:1904064 | positive regulation of cation transmembrane transport | 10/402 | 0.001746927 | 0.008198426 |
| BP | GO:0019233 | sensory perception of pain | 8/402 | 0.00176018 | 0.008246625 |
| BP | GO:0002691 | regulation of cellular extravasation | 5/402 | 0.001811453 | 0.008429691 |
| BP | GO:0048246 | macrophage chemotaxis | 5/402 | 0.001811453 | 0.008429691 |
| BP | GO:0051281 | positive regulation of release of sequestered calcium ion into cytosol | 5/402 | 0.001811453 | 0.008429691 |
| BP | GO:0098751 | bone cell development | 5/402 | 0.001811453 | 0.008429691 |
| BP | GO:0046579 | positive regulation of Ras protein signal transduction | 6/402 | 0.001829793 | 0.008500729 |
| BP | GO:0010001 | glial cell differentiation | 13/402 | 0.001868275 | 0.008620976 |
| BP | GO:0034764 | positive regulation of transmembrane transport | 13/402 | 0.001868275 | 0.008620976 |
| BP | GO:0051048 | negative regulation of secretion | 11/402 | 0.001891518 | 0.008620976 |
| BP | GO:0001779 | natural killer cell differentiation | 4/402 | 0.001892328 | 0.008620976 |

**Table S5b. Analysis of KEGG.**

| ID | Description | GeneRatio | pvalue | qvalue |
| --- | --- | --- | --- | --- |
| hsa04640 | Hematopoietic cell lineage | 21/238 | 1.99E-13 | 3.76E-11 |
| hsa04514 | Cell adhesion molecules | 25/238 | 1.06E-12 | 1.00E-10 |
| hsa04061 | Viral protein interaction with cytokine and cytokine receptor | 20/238 | 2.47E-12 | 1.55E-10 |
| hsa04658 | Th1 and Th2 cell differentiation | 19/238 | 4.87E-12 | 2.29E-10 |
| hsa05150 | Staphylococcus aureus infection | 19/238 | 1.08E-11 | 3.48E-10 |
| hsa04659 | Th17 cell differentiation | 20/238 | 1.11E-11 | 3.48E-10 |
| hsa04062 | Chemokine signaling pathway | 26/238 | 1.45E-11 | 3.91E-10 |
| hsa05332 | Graft-versus-host disease | 13/238 | 5.21E-11 | 1.20E-09 |
| hsa05323 | Rheumatoid arthritis | 18/238 | 5.73E-11 | 1.20E-09 |
| hsa04940 | Type I diabetes mellitus | 13/238 | 7.29E-11 | 1.37E-09 |
| hsa05330 | Allograft rejection | 12/238 | 2.26E-10 | 3.28E-09 |
| hsa05340 | Primary immunodeficiency | 12/238 | 2.26E-10 | 3.28E-09 |
| hsa05140 | Leishmaniasis | 16/238 | 2.26E-10 | 3.28E-09 |
| hsa05169 | Epstein-Barr virus infection | 25/238 | 2.66E-10 | 3.57E-09 |
| hsa04672 | Intestinal immune network for IgA production | 13/238 | 4.51E-10 | 5.67E-09 |
| hsa05416 | Viral myocarditis | 14/238 | 6.11E-10 | 6.93E-09 |
| hsa04060 | Cytokine-cytokine receptor interaction | 30/238 | 6.25E-10 | 6.93E-09 |
| hsa04145 | Phagosome | 21/238 | 1.01E-09 | 1.06E-08 |
| hsa05320 | Autoimmune thyroid disease | 12/238 | 1.54E-08 | 1.53E-07 |
| hsa05321 | Inflammatory bowel disease | 13/238 | 1.90E-08 | 1.79E-07 |
| hsa05152 | Tuberculosis | 21/238 | 2.24E-08 | 2.01E-07 |
| hsa04650 | Natural killer cell mediated cytotoxicity | 17/238 | 1.22E-07 | 1.04E-06 |
| hsa05166 | Human T-cell leukemia virus 1 infection | 22/238 | 1.97E-07 | 1.61E-06 |
| hsa05235 | PD-L1 expression and PD-1 checkpoint pathway in cancer | 13/238 | 9.01E-07 | 7.08E-06 |
| hsa04612 | Antigen processing and presentation | 12/238 | 1.36E-06 | 1.02E-05 |
| hsa05145 | Toxoplasmosis | 14/238 | 2.10E-06 | 1.52E-05 |
| hsa04670 | Leukocyte transendothelial migration | 14/238 | 3.23E-06 | 2.25E-05 |
| hsa05142 | Chagas disease | 13/238 | 4.35E-06 | 2.93E-05 |
| hsa05164 | Influenza A | 17/238 | 4.86E-06 | 3.16E-05 |
| hsa04064 | NF-kappa B signaling pathway | 13/238 | 5.42E-06 | 3.30E-05 |
| hsa04620 | Toll-like receptor signaling pathway | 13/238 | 5.42E-06 | 3.30E-05 |
| hsa05310 | Asthma | 7/238 | 1.73E-05 | 0.00010163 |
| hsa04933 | AGE-RAGE signaling pathway in diabetic complications | 12/238 | 1.91E-05 | 0.000109086 |
| hsa05162 | Measles | 14/238 | 2.71E-05 | 0.00015013 |
| hsa04611 | Platelet activation | 13/238 | 3.72E-05 | 0.000200176 |
| hsa04970 | Salivary secretion | 11/238 | 4.89E-05 | 0.000255722 |
| hsa05143 | African trypanosomiasis | 7/238 | 5.86E-05 | 0.000298567 |
| hsa05144 | Malaria | 8/238 | 6.13E-05 | 0.000303721 |
| hsa04662 | B cell receptor signaling pathway | 10/238 | 0.00010208 | 0.000493181 |
| hsa04660 | T cell receptor signaling pathway | 12/238 | 0.000127285 | 0.000599578 |
| hsa05163 | Human cytomegalovirus infection | 17/238 | 0.000166413 | 0.000764775 |
| hsa04630 | JAK-STAT signaling pathway | 14/238 | 0.000203318 | 0.000912128 |
| hsa04380 | Osteoclast differentiation | 12/238 | 0.000217448 | 0.000952833 |
| hsa05133 | Pertussis | 9/238 | 0.000236423 | 0.001012435 |
| hsa05146 | Amoebiasis | 10/238 | 0.000509339 | 0.002132671 |
| hsa04610 | Complement and coagulation cascades | 9/238 | 0.000600153 | 0.002458291 |
| hsa05170 | Human immunodeficiency virus 1 infection | 15/238 | 0.000802031 | 0.00321531 |
| hsa05417 | Lipid and atherosclerosis | 15/238 | 0.000926488 | 0.003636871 |
| hsa04151 | PI3K-Akt signaling pathway | 21/238 | 0.000976776 | 0.003756022 |
| hsa05135 | Yersinia infection | 11/238 | 0.00145533 | 0.005376762 |
| hsa05322 | Systemic lupus erythematosus | 11/238 | 0.00145533 | 0.005376762 |
| hsa04810 | Regulation of actin cytoskeleton | 15/238 | 0.001745675 | 0.006325421 |
| hsa04664 | Fc epsilon RI signaling pathway | 7/238 | 0.00266314 | 0.009467767 |
| hsa05202 | Transcriptional misregulation in cancer | 13/238 | 0.002740093 | 0.009560949 |
| hsa05167 | Kaposi sarcoma-associated herpesvirus infection | 13/238 | 0.002866142 | 0.009818935 |
| hsa04210 | Apoptosis | 10/238 | 0.004481848 | 0.015079902 |
| hsa04623 | Cytosolic DNA-sensing pathway | 7/238 | 0.004630648 | 0.015307219 |
| hsa04621 | NOD-like receptor signaling pathway | 12/238 | 0.005564573 | 0.017932296 |
| hsa04015 | Rap1 signaling pathway | 13/238 | 0.005615113 | 0.017932296 |
| hsa05130 | Pathogenic Escherichia coli infection | 12/238 | 0.009012741 | 0.02830317 |
| hsa04142 | Lysosome | 9/238 | 0.011054445 | 0.034145741 |
| hsa04512 | ECM-receptor interaction | 7/238 | 0.011611914 | 0.035289177 |

# Appendix 6

**GSEA analysis**

**Table 6a. GO of GSEA analysis.**

| ID | enrichmentScore | NES | pvalue | qvalue |
| --- | --- | --- | --- | --- |
| GOBP_ADAPTIVE_IMMUNE_RESPONSE | 0.761252007 | 3.023057035 | 1.00E-10 | 2.88E-09 |
| GOBP_ADAPTIVE_IMMUNE_RESPONSE_BASED_ON_SOMATIC_RECOMBINATION_OF_IMMUNE_RECEPTORS_BUILT_FROM_IMMUNOGLOBULIN_SUPERFAMILY_DOMAINS | 0.758560038 | 2.924684846 | 1.00E-10 | 2.88E-09 |
| GOBP_LYMPHOCYTE_MEDIATED_IMMUNITY | 0.762414023 | 2.91974071 | 1.00E-10 | 2.88E-09 |
| GOBP_LEUKOCYTE_MEDIATED_IMMUNITY | 0.737544155 | 2.894381251 | 1.00E-10 | 2.88E-09 |
| GOBP_POSITIVE_REGULATION_OF_LEUKOCYTE_CELL_CELL_ADHESION | 0.74035352 | 2.822734036 | 1.00E-10 | 2.88E-09 |
| GOBP_POSITIVE_REGULATION_OF_LYMPHOCYTE_ACTIVATION | 0.727268558 | 2.812385215 | 1.00E-10 | 2.88E-09 |
| GOBP_POSITIVE_REGULATION_OF_CELL_ACTIVATION | 0.715641766 | 2.804645764 | 1.00E-10 | 2.88E-09 |
| GOBP_IMMUNE_RESPONSE_REGULATING_SIGNALING_PATHWAY | 0.705940668 | 2.787867868 | 1.00E-10 | 2.88E-09 |
| GOBP_IMMUNE_RESPONSE_REGULATING_CELL_SURFACE_RECEPTOR_SIGNALING_PATHWAY | 0.719823108 | 2.776714657 | 1.00E-10 | 2.88E-09 |
| GOBP_ACTIVATION_OF_IMMUNE_RESPONSE | 0.695204456 | 2.769065634 | 1.00E-10 | 2.88E-09 |
| GOBP_LEUKOCYTE_MEDIATED_CYTOTOXICITY | 0.789066272 | 2.768488002 | 1.00E-10 | 2.88E-09 |
| GOBP_REGULATION_OF_LYMPHOCYTE_ACTIVATION | 0.693685044 | 2.756976704 | 1.00E-10 | 2.88E-09 |
| GOBP_REGULATION_OF_T_CELL_ACTIVATION | 0.701943967 | 2.752311129 | 1.00E-10 | 2.88E-09 |
| GOBP_ANTIGEN_RECEPTOR_MEDIATED_SIGNALING_PATHWAY | 0.755449499 | 2.751357076 | 1.00E-10 | 2.88E-09 |
| GOBP_LEUKOCYTE_CELL_CELL_ADHESION | 0.696262358 | 2.743775643 | 1.00E-10 | 2.88E-09 |
| GOBP_ANTIGEN_PROCESSING_AND_PRESENTATION | 0.808100919 | 2.742000284 | 1.00E-10 | 2.88E-09 |
| GOBP_ALPHA_BETA_T_CELL_ACTIVATION | 0.752059244 | 2.714176889 | 1.00E-10 | 2.88E-09 |
| GOBP_POSITIVE_REGULATION_OF_CELL_CELL_ADHESION | 0.697125526 | 2.698623121 | 1.00E-10 | 2.88E-09 |
| GOBP_REGULATION_OF_ADAPTIVE_IMMUNE_RESPONSE | 0.73523438 | 2.689897957 | 1.00E-10 | 2.88E-09 |
| GOBP_REGULATION_OF_LEUKOCYTE_MEDIATED_IMMUNITY | 0.710483367 | 2.679689668 | 1.00E-10 | 2.88E-09 |
| GOBP_B_CELL_MEDIATED_IMMUNITY | 0.756093402 | 2.667105846 | 1.00E-10 | 2.88E-09 |
| GOBP_LEUKOCYTE_PROLIFERATION | 0.683592864 | 2.660527523 | 1.00E-10 | 2.88E-09 |
| GOBP_T_CELL_DIFFERENTIATION | 0.688012145 | 2.652380764 | 1.00E-10 | 2.88E-09 |
| GOBP_CELL_ACTIVATION_INVOLVED_IN_IMMUNE_RESPONSE | 0.690708274 | 2.651539142 | 1.00E-10 | 2.88E-09 |
| GOBP_RESPONSE_TO_TYPE_II_INTERFERON | 0.756732756 | 2.648249638 | 1.00E-10 | 2.88E-09 |
| GOBP_POSITIVE_REGULATION_OF_ADAPTIVE_IMMUNE_RESPONSE | 0.763555235 | 2.640906028 | 1.00E-10 | 2.88E-09 |
| GOBP_REGULATION_OF_LEUKOCYTE_PROLIFERATION | 0.691792049 | 2.626763537 | 1.00E-10 | 2.88E-09 |
| GOBP_B_CELL_ACTIVATION | 0.688572196 | 2.625053638 | 1.00E-10 | 2.88E-09 |
| GOBP_SENSORY_PERCEPTION_OF_BITTER_TASTE | -0.830603544 | -2.62104009 | 1.00E-10 | 2.88E-09 |
| GOBP_TYPE_II_INTERFERON_PRODUCTION | 0.76616582 | 2.614181218 | 1.00E-10 | 2.88E-09 |
| GOBP_DETECTION_OF_CHEMICAL_STIMULUS_INVOLVED_IN_SENSORY_PERCEPTION_OF_TASTE | -0.828040973 | -2.611696675 | 1.00E-10 | 2.88E-09 |
| GOBP_REGULATION_OF_IMMUNE_EFFECTOR_PROCESS | 0.666973496 | 2.611408818 | 1.00E-10 | 2.88E-09 |
| GOBP_POSITIVE_REGULATION_OF_T_CELL_PROLIFERATION | 0.766905636 | 2.608835064 | 1.00E-10 | 2.88E-09 |
| GOBP_REGULATION_OF_LEUKOCYTE_MEDIATED_CYTOTOXICITY | 0.78052961 | 2.603416288 | 1.00E-10 | 2.88E-09 |
| GOBP_POSITIVE_REGULATION_OF_LEUKOCYTE_PROLIFERATION | 0.723482585 | 2.601775609 | 1.00E-10 | 2.88E-09 |
| GOBP_LYMPHOCYTE_ACTIVATION_INVOLVED_IN_IMMUNE_RESPONSE | 0.703317304 | 2.591924032 | 1.00E-10 | 2.88E-09 |
| GOBP_REGULATION_OF_LYMPHOCYTE_MEDIATED_IMMUNITY | 0.716281647 | 2.589780177 | 1.00E-10 | 2.88E-09 |
| GOBP_ANTIGEN_PROCESSING_AND_PRESENTATION_OF_PEPTIDE_ANTIGEN | 0.815865521 | 2.5825555 | 1.00E-10 | 2.88E-09 |
| GOBP_T_CELL_MEDIATED_IMMUNITY | 0.74428027 | 2.582091533 | 1.00E-10 | 2.88E-09 |
| GOBP_REGULATION_OF_INNATE_IMMUNE_RESPONSE | 0.661650418 | 2.581558572 | 1.00E-10 | 2.88E-09 |
| GOBP_MYELOID_LEUKOCYTE_ACTIVATION | 0.688607178 | 2.579189573 | 1.00E-10 | 2.88E-09 |
| GOBP_T_CELL_PROLIFERATION | 0.696166958 | 2.572527773 | 1.00E-10 | 2.88E-09 |
| GOBP_MONONUCLEAR_CELL_DIFFERENTIATION | 0.647195453 | 2.572003524 | 1.00E-10 | 2.88E-09 |
| GOBP_POSITIVE_REGULATION_OF_IMMUNE_EFFECTOR_PROCESS | 0.679030423 | 2.569598845 | 1.00E-10 | 2.88E-09 |
| GOBP_T_CELL_RECEPTOR_SIGNALING_PATHWAY | 0.726492125 | 2.564367593 | 1.00E-10 | 2.88E-09 |
| GOBP_POSITIVE_REGULATION_OF_LEUKOCYTE_MEDIATED_IMMUNITY | 0.726457026 | 2.5642437 | 1.00E-10 | 2.88E-09 |
| GOBP_POSITIVE_REGULATION_OF_CYTOKINE_PRODUCTION | 0.644130005 | 2.56324902 | 1.00E-10 | 2.88E-09 |
| GOBP_B_CELL_RECEPTOR_SIGNALING_PATHWAY | 0.800526763 | 2.549508139 | 1.00E-10 | 2.88E-09 |
| GOBP_B_CELL_PROLIFERATION | 0.749371061 | 2.549186507 | 1.00E-10 | 2.88E-09 |
| GOBP_NEUTROPHIL_MIGRATION | 0.733497219 | 2.548706062 | 1.00E-10 | 2.88E-09 |
| GOBP_REGULATION_OF_CELL_KILLING | 0.744306782 | 2.536826573 | 1.00E-10 | 2.88E-09 |
| GOBP_POSITIVE_REGULATION_OF_LYMPHOCYTE_MEDIATED_IMMUNITY | 0.739031322 | 2.534835507 | 1.00E-10 | 2.88E-09 |
| GOMF_IMMUNE_RECEPTOR_ACTIVITY | 0.718431872 | 2.534255478 | 1.00E-10 | 2.88E-09 |
| GOBP_REGULATION_OF_B_CELL_ACTIVATION | 0.728576781 | 2.532359912 | 1.00E-10 | 2.88E-09 |
| GOBP_POSITIVE_REGULATION_OF_RESPONSE_TO_BIOTIC_STIMULUS | 0.65431769 | 2.531778602 | 1.00E-10 | 2.88E-09 |
| GOBP_CELLULAR_RESPONSE_TO_TYPE_II_INTERFERON | 0.741921759 | 2.528697679 | 1.00E-10 | 2.88E-09 |
| GOBP_ALPHA_BETA_T_CELL_DIFFERENTIATION | 0.730114659 | 2.526977635 | 1.00E-10 | 2.88E-09 |
| GOBP_REGULATION_OF_T_CELL_MEDIATED_IMMUNITY | 0.751167768 | 2.524825096 | 1.00E-10 | 2.88E-09 |
| GOBP_POSITIVE_REGULATION_OF_CELL_KILLING | 0.794566399 | 2.524637139 | 1.00E-10 | 2.88E-09 |
| GOBP_REGULATION_OF_ALPHA_BETA_T_CELL_ACTIVATION | 0.740856524 | 2.523746865 | 1.00E-10 | 2.88E-09 |
| GOBP_NEGATIVE_REGULATION_OF_CELL_ACTIVATION | 0.681423783 | 2.521705563 | 1.00E-10 | 2.88E-09 |
| GOBP_POSITIVE_REGULATION_OF_TYPE_II_INTERFERON_PRODUCTION | 0.780537828 | 2.517126237 | 1.00E-10 | 2.88E-09 |
| GOBP_CELL_KILLING | 0.690011466 | 2.509980236 | 1.00E-10 | 2.88E-09 |
| GOBP_REGULATION_OF_T_CELL_DIFFERENTIATION | 0.689546888 | 2.508551862 | 1.00E-10 | 2.88E-09 |
| GOBP_POSITIVE_REGULATION_OF_LYMPHOCYTE_DIFFERENTIATION | 0.713520376 | 2.506589219 | 1.00E-10 | 2.88E-09 |
| GOBP_PRODUCTION_OF_MOLECULAR_MEDIATOR_OF_IMMUNE_RESPONSE | 0.665787905 | 2.500290655 | 1.00E-10 | 2.88E-09 |
| GOBP_NEUTROPHIL_CHEMOTAXIS | 0.73894161 | 2.499244077 | 1.00E-10 | 2.88E-09 |
| GOBP_NEGATIVE_REGULATION_OF_LYMPHOCYTE_ACTIVATION | 0.691992222 | 2.49136635 | 1.00E-10 | 2.88E-09 |
| GOBP_REGULATION_OF_ANTIGEN_RECEPTOR_MEDIATED_SIGNALING_PATHWAY | 0.786245909 | 2.488797044 | 1.00E-10 | 2.88E-09 |
| GOBP_T_CELL_ACTIVATION_INVOLVED_IN_IMMUNE_RESPONSE | 0.718481738 | 2.486715286 | 1.00E-10 | 2.88E-09 |
| GOBP_NATURAL_KILLER_CELL_MEDIATED_IMMUNITY | 0.772032667 | 2.483301968 | 1.00E-10 | 2.88E-09 |
| GOBP_POSITIVE_REGULATION_OF_DEFENSE_RESPONSE | 0.628102827 | 2.481386226 | 1.00E-10 | 2.88E-09 |
| GOCC_EXTERNAL_SIDE_OF_PLASMA_MEMBRANE | 0.635003712 | 2.480039479 | 1.00E-10 | 2.88E-09 |
| GOBP_ANTIGEN_PROCESSING_AND_PRESENTATION_OF_EXOGENOUS_ANTIGEN | 0.849877519 | 2.479629295 | 1.00E-10 | 2.88E-09 |
| GOBP_REGULATION_OF_RESPONSE_TO_BIOTIC_STIMULUS | 0.624180334 | 2.476281943 | 1.00E-10 | 2.88E-09 |
| GOBP_POSITIVE_REGULATION_OF_ALPHA_BETA_T_CELL_ACTIVATION | 0.774371442 | 2.476180791 | 1.00E-10 | 2.88E-09 |
| GOBP_NEGATIVE_REGULATION_OF_IMMUNE_SYSTEM_PROCESS | 0.621242834 | 2.469299751 | 1.00E-10 | 2.88E-09 |
| GOBP_PHAGOCYTOSIS | 0.662260708 | 2.469291577 | 1.00E-10 | 2.88E-09 |
| GOBP_REGULATION_OF_CELL_CELL_ADHESION | 0.62210841 | 2.469196852 | 1.00E-10 | 2.88E-09 |
| GOBP_INTERLEUKIN_12_PRODUCTION | 0.777436607 | 2.46021612 | 1.00E-10 | 2.88E-09 |
| GOBP_REGULATION_OF_LYMPHOCYTE_DIFFERENTIATION | 0.665037194 | 2.459867605 | 1.00E-10 | 2.88E-09 |
| GOBP_NEGATIVE_REGULATION_OF_IMMUNE_RESPONSE | 0.675276322 | 2.453144857 | 1.00E-10 | 2.88E-09 |
| GOBP_NATURAL_KILLER_CELL_ACTIVATION | 0.726892621 | 2.451723092 | 1.00E-10 | 2.88E-09 |
| GOBP_ACTIVATION_OF_INNATE_IMMUNE_RESPONSE | 0.659494872 | 2.448717619 | 1.00E-10 | 2.88E-09 |
| GOBP_CD4_POSITIVE_ALPHA_BETA_T_CELL_ACTIVATION | 0.717089983 | 2.4454875 | 1.00E-10 | 2.88E-09 |
| GOBP_GRANULOCYTE_MIGRATION | 0.681686385 | 2.443656263 | 1.00E-10 | 2.88E-09 |
| GOBP_TUMOR_NECROSIS_FACTOR_SUPERFAMILY_CYTOKINE_PRODUCTION | 0.677711538 | 2.437531604 | 1.00E-10 | 2.88E-09 |
| GOBP_IMMUNOGLOBULIN_PRODUCTION | 0.704371493 | 2.43620741 | 1.00E-10 | 2.88E-09 |
| GOBP_REGULATION_OF_LEUKOCYTE_DIFFERENTIATION | 0.628894411 | 2.433023332 | 1.00E-10 | 2.88E-09 |
| GOBP_CD4_POSITIVE_ALPHA_BETA_T_CELL_DIFFERENTIATION | 0.729307602 | 2.429840071 | 1.00E-10 | 2.88E-09 |
| GOBP_REGULATION_OF_PHAGOCYTOSIS | 0.720673249 | 2.429299246 | 1.00E-10 | 2.88E-09 |
| GOBP_RESPONSE_TO_VIRUS | 0.618315278 | 2.425183954 | 1.00E-10 | 2.88E-09 |
| GOMF_ANTIGEN_BINDING | 0.773579546 | 2.424038086 | 1.00E-10 | 2.88E-09 |
| GOCC_MHC_PROTEIN_COMPLEX | 0.939738441 | 2.418198421 | 1.00E-10 | 2.88E-09 |
| GOBP_LEUKOCYTE_HOMEOSTASIS | 0.712740453 | 2.417615032 | 1.00E-10 | 2.88E-09 |
| GOBP_T_CELL_MEDIATED_CYTOTOXICITY | 0.799054974 | 2.414063537 | 1.00E-10 | 2.88E-09 |
| GOBP_INNATE_IMMUNE_RESPONSE_ACTIVATING_SIGNALING_PATHWAY | 0.657862453 | 2.4118232 | 1.00E-10 | 2.88E-09 |
| GOBP_POSITIVE_REGULATION_OF_HEMOPOIESIS | 0.662000044 | 2.411012921 | 1.00E-10 | 2.88E-09 |
| GOBP_POSITIVE_REGULATION_OF_CELL_ADHESION | 0.607413428 | 2.410871322 | 1.00E-10 | 2.88E-09 |
| GOBP_T_CELL_SELECTION | 0.800884322 | 2.406584894 | 1.00E-10 | 2.88E-09 |
| GOBP_CYTOKINE_MEDIATED_SIGNALING_PATHWAY | 0.605574194 | 2.405070636 | 1.00E-10 | 2.88E-09 |
| GOBP_REGULATION_OF_B_CELL_PROLIFERATION | 0.759067156 | 2.402764926 | 1.00E-10 | 2.88E-09 |
| GOBP_INTERLEUKIN_6_PRODUCTION | 0.670748833 | 2.397766617 | 1.00E-10 | 2.88E-09 |
| GOBP_POSITIVE_REGULATION_OF_TUMOR_NECROSIS_FACTOR_SUPERFAMILY_CYTOKINE_PRODUCTION | 0.705904605 | 2.396026984 | 1.00E-10 | 2.88E-09 |
| GOBP_POSITIVE_REGULATION_OF_T_CELL_MEDIATED_IMMUNITY | 0.760215274 | 2.394703645 | 1.00E-10 | 2.88E-09 |
| GOBP_ANTIGEN_PROCESSING_AND_PRESENTATION_OF_EXOGENOUS_PEPTIDE_ANTIGEN | 0.855768597 | 2.393970482 | 1.00E-10 | 2.88E-09 |
| GOBP_REGULATION_OF_ALPHA_BETA_T_CELL_DIFFERENTIATION | 0.744948537 | 2.386256498 | 1.00E-10 | 2.88E-09 |
| GOBP_INTERFERON_MEDIATED_SIGNALING_PATHWAY | 0.705612272 | 2.379946985 | 1.00E-10 | 2.88E-09 |
| GOBP_IMMUNOGLOBULIN_PRODUCTION_INVOLVED_IN_IMMUNOGLOBULIN_MEDIATED_IMMUNE_RESPONSE | 0.75114357 | 2.377011197 | 1.00E-10 | 2.88E-09 |
| GOBP_POSITIVE_REGULATION_OF_PHAGOCYTOSIS | 0.746260954 | 2.371560089 | 1.00E-10 | 2.88E-09 |
| GOBP_POSITIVE_REGULATION_OF_B_CELL_ACTIVATION | 0.732457969 | 2.371373823 | 1.00E-10 | 2.88E-09 |
| GOBP_LEUKOCYTE_CHEMOTAXIS | 0.635472245 | 2.36940867 | 1.00E-10 | 2.88E-09 |
| GOBP_NEGATIVE_REGULATION_OF_IMMUNE_EFFECTOR_PROCESS | 0.680026646 | 2.363611167 | 1.00E-10 | 2.88E-09 |
| GOBP_MACROPHAGE_ACTIVATION | 0.701180917 | 2.363593037 | 1.00E-10 | 2.88E-09 |
| GOBP_REGULATION_OF_PRODUCTION_OF_MOLECULAR_MEDIATOR_OF_IMMUNE_RESPONSE | 0.64968252 | 2.362515546 | 1.00E-10 | 2.88E-09 |
| GOBP_NEGATIVE_REGULATION_OF_LEUKOCYTE_MEDIATED_IMMUNITY | 0.743290953 | 2.362121654 | 1.00E-10 | 2.88E-09 |
| GOBP_MYELOID_CELL_ACTIVATION_INVOLVED_IN_IMMUNE_RESPONSE | 0.698980926 | 2.360903876 | 1.00E-10 | 2.88E-09 |
| GOBP_GRANULOCYTE_CHEMOTAXIS | 0.679675629 | 2.35674822 | 1.00E-10 | 2.88E-09 |
| GOBP_INTERLEUKIN_10_PRODUCTION | 0.761423038 | 2.35332264 | 1.00E-10 | 2.88E-09 |
| GOBP_REGULATION_OF_T_CELL_MEDIATED_CYTOTOXICITY | 0.808705958 | 2.351653155 | 1.00E-10 | 2.88E-09 |
| GOBP_POSITIVE_REGULATION_OF_T_CELL_MEDIATED_CYTOTOXICITY | 0.845880037 | 2.351491527 | 1.00E-10 | 2.88E-09 |
| GOBP_CELLULAR_DEFENSE_RESPONSE | 0.796399243 | 2.350770755 | 1.00E-10 | 2.88E-09 |
| GOCC_IMMUNOLOGICAL_SYNAPSE | 0.818197041 | 2.346336207 | 1.00E-10 | 2.88E-09 |
| GOBP_MONONUCLEAR_CELL_MIGRATION | 0.644578949 | 2.345421822 | 1.00E-10 | 2.88E-09 |
| GOBP_LEUKOCYTE_MIGRATION | 0.597369427 | 2.343029196 | 1.00E-10 | 2.88E-09 |
| GOBP_MYELOID_LEUKOCYTE_MIGRATION | 0.622419467 | 2.331282406 | 1.00E-10 | 2.88E-09 |
| GOBP_REGULATION_OF_INFLAMMATORY_RESPONSE | 0.594479602 | 2.330192647 | 1.00E-10 | 2.88E-09 |
| GOBP_B_CELL_DIFFERENTIATION | 0.649768771 | 2.328744925 | 1.00E-10 | 2.88E-09 |
| GOBP_LYMPHOCYTE_MIGRATION | 0.681906064 | 2.325500002 | 1.00E-10 | 2.88E-09 |
| GOBP_LYMPHOCYTE_COSTIMULATION | 0.794251152 | 2.325434209 | 1.00E-10 | 2.88E-09 |
| GOBP_REGULATION_OF_HEMOPOIESIS | 0.589632872 | 2.323578596 | 1.00E-10 | 2.88E-09 |
| GOBP_INTERLEUKIN_1_PRODUCTION | 0.681130622 | 2.322855516 | 1.00E-10 | 2.88E-09 |
| GOBP_LEUKOCYTE_DEGRANULATION | 0.711381149 | 2.318051344 | 1.00E-10 | 2.88E-09 |
| GOBP_DEFENSE_RESPONSE_TO_SYMBIONT | 0.603908221 | 2.31470636 | 1.00E-10 | 2.88E-09 |
| GOBP_RESPONSE_TO_MOLECULE_OF_BACTERIAL_ORIGIN | 0.594358696 | 2.314185485 | 1.00E-10 | 2.88E-09 |
| GOBP_CYTOKINE_PRODUCTION_INVOLVED_IN_IMMUNE_RESPONSE | 0.666496804 | 2.312241534 | 1.00E-10 | 2.88E-09 |
| GOBP_CELLULAR_RESPONSE_TO_BIOTIC_STIMULUS | 0.612019447 | 2.306807803 | 1.00E-10 | 2.88E-09 |
| GOBP_CELL_CHEMOTAXIS | 0.598274508 | 2.298346034 | 1.00E-10 | 2.88E-09 |
| GOBP_MYELOID_LEUKOCYTE_MEDIATED_IMMUNITY | 0.674510731 | 2.297738211 | 1.00E-10 | 2.88E-09 |
| GOBP_NEGATIVE_REGULATION_OF_CYTOKINE_PRODUCTION | 0.599075416 | 2.288506931 | 1.00E-10 | 2.88E-09 |
| GOBP_POSITIVE_REGULATION_OF_PRODUCTION_OF_MOLECULAR_MEDIATOR_OF_IMMUNE_RESPONSE | 0.652148281 | 2.275442444 | 1.00E-10 | 2.88E-09 |
| GOBP_NEGATIVE_REGULATION_OF_DEFENSE_RESPONSE | 0.602831068 | 2.272175203 | 1.00E-10 | 2.88E-09 |
| GOBP_NEGATIVE_REGULATION_OF_LEUKOCYTE_CELL_CELL_ADHESION | 0.643640435 | 2.270430031 | 1.00E-10 | 2.88E-09 |
| GOBP_PEPTIDE_ANTIGEN_ASSEMBLY_WITH_MHC_PROTEIN_COMPLEX | 0.947095816 | 2.269311418 | 1.00E-10 | 2.88E-09 |
| GOBP_CELLULAR_RESPONSE_TO_MOLECULE_OF_BACTERIAL_ORIGIN | 0.612488011 | 2.265693372 | 1.00E-10 | 2.88E-09 |
| GOCC_FICOLIN_1_RICH_GRANULE | 0.62226702 | 2.262821398 | 1.00E-10 | 2.88E-09 |
| GOMF_CYTOKINE_RECEPTOR_BINDING | 0.5917849 | 2.234823814 | 1.00E-10 | 2.88E-09 |
| GOBP_REGULATION_OF_RESPONSE_TO_CYTOKINE_STIMULUS | 0.617301017 | 2.21238181 | 1.00E-10 | 2.88E-09 |
| GOBP_RECEPTOR_SIGNALING_PATHWAY_VIA_STAT | 0.608923723 | 2.206870793 | 1.00E-10 | 2.88E-09 |
| GOCC_MHC_CLASS_II_PROTEIN_COMPLEX | 0.964835266 | 2.206022745 | 1.00E-10 | 2.88E-09 |
| GOCC_TERTIARY_GRANULE | 0.609665807 | 2.192439567 | 1.00E-10 | 2.88E-09 |
| GOCC_CHROMOSOMAL_REGION | 0.560298281 | 2.187934463 | 1.00E-10 | 2.88E-09 |
| GOBP_REGULATION_OF_CHROMOSOME_ORGANIZATION | 0.57339842 | 2.1641429 | 1.00E-10 | 2.88E-09 |
| GOBP_REGULATION_OF_LEUKOCYTE_MIGRATION | 0.580968655 | 2.163922716 | 1.00E-10 | 2.88E-09 |
| GOBP_PEPTIDE_ANTIGEN_ASSEMBLY_WITH_MHC_CLASS_II_PROTEIN_COMPLEX | 0.964790685 | 2.160926898 | 1.00E-10 | 2.88E-09 |
| GOBP_RESPONSE_TO_TUMOR_NECROSIS_FACTOR | 0.569938267 | 2.149603716 | 1.00E-10 | 2.88E-09 |
| GOBP_DNA_RECOMBINATION | 0.557720444 | 2.143823266 | 1.00E-10 | 2.88E-09 |
| GOBP_I_KAPPAB_KINASE_NF_KAPPAB_SIGNALING | 0.557143786 | 2.128732104 | 1.00E-10 | 2.88E-09 |
| GOBP_HOMEOSTASIS_OF_NUMBER_OF_CELLS | 0.541872727 | 2.088993354 | 1.00E-10 | 2.88E-09 |
| GOBP_CHROMOSOME_SEGREGATION | 0.520956417 | 2.053317102 | 1.00E-10 | 2.88E-09 |
| GOCC_SECRETORY_GRANULE_MEMBRANE | 0.529738669 | 2.05065654 | 1.00E-10 | 2.88E-09 |
| GOCC_ENDOCYTIC_VESICLE | 0.526219945 | 2.048035783 | 1.00E-10 | 2.88E-09 |
| GOBP_MYELOID_CELL_DIFFERENTIATION | 0.519108683 | 2.045662448 | 1.00E-10 | 2.88E-09 |
| GOCC_VESICLE_LUMEN | 0.522632413 | 2.022646501 | 1.00E-10 | 2.88E-09 |
| GOBP_NEGATIVE_REGULATION_OF_RESPONSE_TO_EXTERNAL_STIMULUS | 0.510522629 | 2.012775265 | 1.00E-10 | 2.88E-09 |
| GOCC_COLLAGEN_CONTAINING_EXTRACELLULAR_MATRIX | 0.490955057 | 1.937401852 | 1.00E-10 | 2.88E-09 |
| GOBP_POSITIVE_REGULATION_OF_CELL_DEVELOPMENT | 0.487320635 | 1.932718757 | 1.00E-10 | 2.88E-09 |
| GOBP_T_CELL_DIFFERENTIATION_INVOLVED_IN_IMMUNE_RESPONSE | 0.709657016 | 2.312433217 | 1.07E-10 | 3.06E-09 |
| GOBP_POSITIVE_REGULATION_OF_LEUKOCYTE_MIGRATION | 0.620224701 | 2.211851746 | 1.12E-10 | 3.18E-09 |
| GOBP_VIRAL_PROCESS | 0.482032167 | 1.900114693 | 1.39E-10 | 3.94E-09 |
| GOBP_TYPE_I_INTERFERON_PRODUCTION | 0.679484072 | 2.291819647 | 1.58E-10 | 4.46E-09 |
| GOBP_HUMORAL_IMMUNE_RESPONSE | 0.557782757 | 2.08579183 | 1.61E-10 | 4.50E-09 |
| GOBP_POSITIVE_REGULATION_OF_INTERLEUKIN_1_PRODUCTION | 0.734685424 | 2.339817422 | 1.71E-10 | 4.75E-09 |
| GOBP_NEGATIVE_REGULATION_OF_LEUKOCYTE_PROLIFERATION | 0.684631544 | 2.301183541 | 1.78E-10 | 4.92E-09 |
| GOBP_T_CELL_DIFFERENTIATION_IN_THYMUS | 0.701794149 | 2.311570719 | 2.00E-10 | 5.50E-09 |
| GOCC_LUMENAL_SIDE_OF_ENDOPLASMIC_RETICULUM_MEMBRANE | 0.889368066 | 2.333586682 | 2.03E-10 | 5.56E-09 |
| GOBP_REGULATION_OF_CD4_POSITIVE_ALPHA_BETA_T_CELL_ACTIVATION | 0.730367223 | 2.335470021 | 2.30E-10 | 6.24E-09 |
| GOBP_REGULATION_OF_VESICLE_MEDIATED_TRANSPORT | 0.46111471 | 1.844619667 | 2.31E-10 | 6.24E-09 |
| GOBP_ANTIGEN_PROCESSING_AND_PRESENTATION_OF_ENDOGENOUS_ANTIGEN | 0.86816947 | 2.344541282 | 2.37E-10 | 6.37E-09 |
| GOBP_NUCLEAR_CHROMOSOME_SEGREGATION | 0.514554089 | 1.986703305 | 2.45E-10 | 6.56E-09 |
| GOMF_MHC_PROTEIN_COMPLEX_BINDING | 0.867633317 | 2.343093371 | 2.53E-10 | 6.74E-09 |
| GOBP_RESPONSE_TO_CHEMOKINE | 0.675933607 | 2.28358376 | 2.71E-10 | 7.16E-09 |
| GOMF_CYTOKINE_RECEPTOR_ACTIVITY | 0.681185586 | 2.296516873 | 3.28E-10 | 8.62E-09 |
| GOBP_POSITIVE_REGULATION_OF_INFLAMMATORY_RESPONSE | 0.616464122 | 2.189023201 | 3.34E-10 | 8.75E-09 |
| GOBP_POSITIVE_T_CELL_SELECTION | 0.811896165 | 2.304611878 | 3.55E-10 | 9.23E-09 |
| GOBP_ANTIGEN_PROCESSING_AND_PRESENTATION_OF_PEPTIDE_OR_POLYSACCHARIDE_ANTIGEN_VIA_MHC_CLASS_II | 0.845763277 | 2.324152735 | 4.48E-10 | 1.16E-08 |
| GOBP_NEUROINFLAMMATORY_RESPONSE | 0.728302011 | 2.309723662 | 4.54E-10 | 1.17E-08 |
| GOCC_LUMENAL_SIDE_OF_MEMBRANE | 0.85142518 | 2.323026852 | 4.74E-10 | 1.21E-08 |
| GOCC_MEMBRANE_MICRODOMAIN | 0.503292584 | 1.950530247 | 4.91E-10 | 1.25E-08 |
| GOBP_DENDRITIC_CELL_MIGRATION | 0.833976028 | 2.316292917 | 5.06E-10 | 1.28E-08 |
| GOBP_REGULATION_OF_MONONUCLEAR_CELL_MIGRATION | 0.632123933 | 2.187824913 | 5.69E-10 | 1.43E-08 |
| GOBP_REGULATION_OF_CHEMOTAXIS | 0.550507913 | 2.047821033 | 5.91E-10 | 1.48E-08 |
| GOBP_NEGATIVE_REGULATION_OF_INTERLEUKIN_6_PRODUCTION | 0.77231716 | 2.268920049 | 6.96E-10 | 1.74E-08 |
| GOCC_PLASMA_MEMBRANE_SIGNALING_RECEPTOR_COMPLEX | 0.566628494 | 2.070005981 | 7.25E-10 | 1.80E-08 |
| GOBP_IMMUNE_SYSTEM_DEVELOPMENT | 0.574845369 | 2.096865457 | 7.49E-10 | 1.85E-08 |
| GOBP_MAST_CELL_ACTIVATION | 0.716848594 | 2.296245353 | 7.84E-10 | 1.93E-08 |
| GOBP_ANTIGEN_PROCESSING_AND_PRESENTATION_OF_ENDOGENOUS_PEPTIDE_ANTIGEN | 0.895125092 | 2.303396339 | 8.81E-10 | 2.15E-08 |
| GOBP_CELLULAR_RESPONSE_TO_VIRUS | 0.72004816 | 2.316089835 | 8.90E-10 | 2.16E-08 |
| GOBP_MYELOID_LEUKOCYTE_DIFFERENTIATION | 0.553471825 | 2.066919406 | 9.67E-10 | 2.34E-08 |
| GOBP_LEUKOCYTE_APOPTOTIC_PROCESS | 0.640042209 | 2.210437015 | 9.80E-10 | 2.36E-08 |
| GOBP_REGULATION_OF_CD4_POSITIVE_ALPHA_BETA_T_CELL_DIFFERENTIATION | 0.761700924 | 2.288842331 | 1.16E-09 | 2.78E-08 |
| GOBP_SENSORY_PERCEPTION_OF_TASTE | -0.733331327 | -2.545368485 | 1.31E-09 | 3.11E-08 |
| GOMF_CHEMOKINE_RECEPTOR_BINDING | 0.75586105 | 2.297527559 | 1.47E-09 | 3.49E-08 |
| GOCC_TERTIARY_GRANULE_MEMBRANE | 0.715565909 | 2.301672332 | 1.53E-09 | 3.60E-08 |
| GOBP_LYMPHOCYTE_HOMEOSTASIS | 0.713986536 | 2.296592158 | 1.77E-09 | 4.13E-08 |
| GOBP_REGULATION_OF_MYELOID_LEUKOCYTE_MEDIATED_IMMUNITY | 0.734968721 | 2.283422816 | 1.76E-09 | 4.13E-08 |
| GOBP_RNA_LOCALIZATION | 0.566627981 | 2.058224492 | 1.99E-09 | 4.63E-08 |
| GOBP_ANTIGEN_PROCESSING_AND_PRESENTATION_OF_EXOGENOUS_PEPTIDE_ANTIGEN_VIA_MHC_CLASS_II | 0.863095459 | 2.302554625 | 2.12E-09 | 4.91E-08 |
| GOBP_INFLAMMATORY_RESPONSE_TO_ANTIGENIC_STIMULUS | 0.713532718 | 2.272450535 | 2.16E-09 | 4.97E-08 |
| GOBP_NEGATIVE_REGULATION_OF_INFLAMMATORY_RESPONSE | 0.592441667 | 2.114760223 | 2.28E-09 | 5.23E-08 |
| GOMF_PEPTIDE_ANTIGEN_BINDING | 0.862116535 | 2.299943063 | 2.36E-09 | 5.38E-08 |
| GOBP_NEGATIVE_REGULATION_OF_INNATE_IMMUNE_RESPONSE | 0.710499358 | 2.285375386 | 2.49E-09 | 5.62E-08 |
| GOBP_NEGATIVE_REGULATION_OF_LYMPHOCYTE_MEDIATED_IMMUNITY | 0.734866221 | 2.256037581 | 2.49E-09 | 5.62E-08 |
| GOCC_ENDOCYTIC_VESICLE_MEMBRANE | 0.559224849 | 2.041831019 | 2.55E-09 | 5.71E-08 |
| GOCC_ENDOSOME_MEMBRANE | 0.443864839 | 1.777041887 | 2.55E-09 | 5.71E-08 |
| GOBP_REGULATION_OF_LEUKOCYTE_APOPTOTIC_PROCESS | 0.672390483 | 2.242723804 | 2.81E-09 | 6.25E-08 |
| GOCC_SPECIFIC_GRANULE_MEMBRANE | 0.66389372 | 2.226389138 | 2.87E-09 | 6.38E-08 |
| GOCC_PHAGOCYTIC_VESICLE | 0.595816712 | 2.111516741 | 3.44E-09 | 7.59E-08 |
| GOCC_CHROMOSOME_CENTROMERIC_REGION | 0.538870025 | 2.009219631 | 3.55E-09 | 7.80E-08 |
| GOBP_REGULATION_OF_LEUKOCYTE_CHEMOTAXIS | 0.62632548 | 2.172877319 | 3.59E-09 | 7.85E-08 |
| GOBP_POSITIVE_REGULATION_OF_I_KAPPAB_KINASE_NF_KAPPAB_SIGNALING | 0.567753923 | 2.062537321 | 4.08E-09 | 8.89E-08 |
| GOBP_POSITIVE_REGULATION_OF_ALPHA_BETA_T_CELL_DIFFERENTIATION | 0.759861248 | 2.260624917 | 4.62E-09 | 1.00E-07 |
| GOMF_CCR_CHEMOKINE_RECEPTOR_BINDING | 0.823696477 | 2.271428621 | 4.81E-09 | 1.04E-07 |
| GOBP_B_CELL_ACTIVATION_INVOLVED_IN_IMMUNE_RESPONSE | 0.677904449 | 2.221395485 | 5.73E-09 | 1.23E-07 |
| GOBP_SMALL_GTPASE_MEDIATED_SIGNAL_TRANSDUCTION | 0.445314597 | 1.767282546 | 5.77E-09 | 1.24E-07 |
| GOMF_MHC_CLASS_II_PROTEIN_COMPLEX_BINDING | 0.876868677 | 2.282654477 | 5.86E-09 | 1.25E-07 |
| GOCC_T_CELL_RECEPTOR_COMPLEX | 0.898947999 | 2.249924923 | 6.02E-09 | 1.28E-07 |
| GOBP_NEGATIVE_REGULATION_OF_CELL_CELL_ADHESION | 0.557446159 | 2.028372764 | 6.14E-09 | 1.30E-07 |
| GOBP_SISTER_CHROMATID_SEGREGATION | 0.523914809 | 1.982723148 | 6.52E-09 | 1.37E-07 |
| GOBP_NIK_NF_KAPPAB_SIGNALING | 0.613012799 | 2.130686947 | 6.56E-09 | 1.37E-07 |
| GOBP_GLIAL_CELL_ACTIVATION | 0.751257544 | 2.207050927 | 7.13E-09 | 1.49E-07 |
| GOBP_FC_RECEPTOR_MEDIATED_STIMULATORY_SIGNALING_PATHWAY | 0.819462943 | 2.259754212 | 7.55E-09 | 1.56E-07 |
| GOCC_VACUOLAR_MEMBRANE | 0.456599817 | 1.809885071 | 7.54E-09 | 1.56E-07 |
| GOBP_DOUBLE_STRAND_BREAK_REPAIR | 0.502918558 | 1.921548631 | 9.52E-09 | 1.96E-07 |
| GOBP_POSITIVE_REGULATION_OF_NF_KAPPAB_TRANSCRIPTION_FACTOR_ACTIVITY | 0.580418691 | 2.086107752 | 9.67E-09 | 1.98E-07 |
| GOBP_NEGATIVE_REGULATION_OF_ANTIGEN_RECEPTOR_MEDIATED_SIGNALING_PATHWAY | 0.811245374 | 2.253160582 | 1.08E-08 | 2.20E-07 |
| GOBP_CYTOSOLIC_PATTERN_RECOGNITION_RECEPTOR_SIGNALING_PATHWAY | 0.659775379 | 2.200646774 | 1.23E-08 | 2.49E-07 |
| GOCC_CHROMOSOME_TELOMERIC_REGION | 0.593434343 | 2.098709702 | 1.36E-08 | 2.76E-07 |
| GOBP_ANTIGEN_PROCESSING_AND_PRESENTATION_OF_PEPTIDE_ANTIGEN_VIA_MHC_CLASS_I | 0.808180507 | 2.244648192 | 1.44E-08 | 2.91E-07 |
| GOBP_REGULATION_OF_T_CELL_RECEPTOR_SIGNALING_PATHWAY | 0.766130382 | 2.227846739 | 1.48E-08 | 2.97E-07 |
| GOBP_LEUKOCYTE_ACTIVATION_INVOLVED_IN_INFLAMMATORY_RESPONSE | 0.761298098 | 2.221187199 | 1.50E-08 | 2.99E-07 |
| GOBP_TOLERANCE_INDUCTION | 0.83654004 | 2.259124199 | 1.54E-08 | 3.06E-07 |
| GOBP_POSITIVE_REGULATION_OF_CYTOKINE_PRODUCTION_INVOLVED_IN_IMMUNE_RESPONSE | 0.674940027 | 2.185156256 | 1.58E-08 | 3.12E-07 |
| GOBP_NEGATIVE_REGULATION_OF_CELL_ADHESION | 0.504908237 | 1.93808336 | 1.60E-08 | 3.16E-07 |
| GOBP_INTERLEUKIN_2_PRODUCTION | 0.720830278 | 2.265382776 | 1.74E-08 | 3.40E-07 |
| GOBP_POSITIVE_REGULATION_OF_CHEMOKINE_PRODUCTION | 0.695453759 | 2.214872881 | 1.73E-08 | 3.40E-07 |
| GOBP_NEGATIVE_REGULATION_OF_INTERLEUKIN_10_PRODUCTION | 0.875329955 | 2.222194023 | 1.84E-08 | 3.58E-07 |
| GOBP_PROTEIN_LOCALIZATION_TO_CHROMOSOME | 0.648062621 | 2.159155508 | 1.91E-08 | 3.70E-07 |
| GOBP_REGULATION_OF_DNA_METABOLIC_PROCESS | 0.438808035 | 1.750196058 | 1.98E-08 | 3.83E-07 |
| GOBP_DENDRITIC_CELL_CHEMOTAXIS | 0.844198206 | 2.252140782 | 2.43E-08 | 4.68E-07 |
| GOBP_POSITIVE_REGULATION_OF_MONONUCLEAR_CELL_MIGRATION | 0.685524667 | 2.195906979 | 2.57E-08 | 4.93E-07 |
| GOCC_CONDENSED_CHROMOSOME | 0.49879031 | 1.886825041 | 2.72E-08 | 5.20E-07 |
| GOCC_FICOLIN_1_RICH_GRANULE_LUMEN | 0.603566881 | 2.09108877 | 2.80E-08 | 5.32E-07 |
| GOBP_FC_GAMMA_RECEPTOR_SIGNALING_PATHWAY | 0.806418662 | 2.223783251 | 3.05E-08 | 5.78E-07 |
| GOCC_APICAL_JUNCTION_COMPLEX | -0.538328057 | -2.125876254 | 3.34E-08 | 6.30E-07 |
| GOBP_HEMOSTASIS | 0.513453286 | 1.923147772 | 3.45E-08 | 6.48E-07 |
| GOMF_NON_MEMBRANE_SPANNING_PROTEIN_TYROSINE_KINASE_ACTIVITY | 0.75446689 | 2.175541325 | 3.49E-08 | 6.55E-07 |
| GOBP_PRODUCTION_OF_MOLECULAR_MEDIATOR_INVOLVED_IN_INFLAMMATORY_RESPONSE | 0.658067801 | 2.151124668 | 3.63E-08 | 6.77E-07 |
| GOBP_TYPE_II_INTERFERON_MEDIATED_SIGNALING_PATHWAY | 0.846761681 | 2.221793042 | 3.66E-08 | 6.80E-07 |
| GOBP_REGULATION_OF_IMMUNOGLOBULIN_PRODUCTION | 0.689945165 | 2.192592831 | 3.73E-08 | 6.90E-07 |
| GOBP_REGULATION_OF_INFLAMMATORY_RESPONSE_TO_ANTIGENIC_STIMULUS | 0.773437037 | 2.197854338 | 3.76E-08 | 6.93E-07 |
| GOBP_NEGATIVE_REGULATION_OF_CELL_KILLING | 0.829852513 | 2.241064149 | 4.01E-08 | 7.36E-07 |
| GOBP_REGULATION_OF_NATURAL_KILLER_CELL_MEDIATED_IMMUNITY | 0.743214061 | 2.185880938 | 4.40E-08 | 8.04E-07 |
| GOBP_BIOLOGICAL_PROCESS_INVOLVED_IN_SYMBIOTIC_INTERACTION | 0.485731531 | 1.869926143 | 4.45E-08 | 8.11E-07 |
| GOBP_GAMMA_DELTA_T_CELL_ACTIVATION | 0.863548613 | 2.222141612 | 4.66E-08 | 8.46E-07 |
| GOBP_POSITIVE_REGULATION_OF_INTERLEUKIN_12_PRODUCTION | 0.75366492 | 2.191598158 | 4.78E-08 | 8.64E-07 |
| GOBP_POSITIVE_REGULATION_OF_CHEMOTAXIS | 0.579557607 | 2.057970614 | 4.94E-08 | 8.91E-07 |
| GOMF_CHEMOKINE_ACTIVITY | 0.770141676 | 2.208528276 | 5.14E-08 | 9.23E-07 |
| GOBP_KIDNEY_EPITHELIUM_DEVELOPMENT | -0.523388762 | -2.095608291 | 5.34E-08 | 9.55E-07 |
| GOBP_INNATE_IMMUNE_RESPONSE_ACTIVATING_CELL_SURFACE_RECEPTOR_SIGNALING_PATHWAY | 0.684013741 | 2.200182376 | 5.47E-08 | 9.76E-07 |
| GOBP_VIRAL_LIFE_CYCLE | 0.478218069 | 1.844160627 | 5.54E-08 | 9.85E-07 |
| GOBP_REGULATION_OF_CHROMOSOME_SEGREGATION | 0.585728033 | 2.055063165 | 5.97E-08 | 1.06E-06 |
| GOBP_NEGATIVE_REGULATION_OF_ADAPTIVE_IMMUNE_RESPONSE | 0.696860155 | 2.183635239 | 6.07E-08 | 1.07E-06 |
| GOBP_POSITIVE_REGULATION_OF_LEUKOCYTE_CHEMOTAXIS | 0.639111896 | 2.159145466 | 6.33E-08 | 1.11E-06 |
| GOMF_G_PROTEIN_COUPLED_CHEMOATTRACTANT_RECEPTOR_ACTIVITY | 0.835633184 | 2.206915978 | 6.54E-08 | 1.14E-06 |
| GOBP_RESPONSE_TO_TYPE_I_INTERFERON | 0.658873734 | 2.150774643 | 7.01E-08 | 1.22E-06 |
| GOBP_POSITIVE_REGULATION_OF_ALPHA_BETA_T_CELL_PROLIFERATION | 0.834506823 | 2.203941248 | 7.51E-08 | 1.30E-06 |
| GOBP_REGULATORY_T_CELL_DIFFERENTIATION | 0.778526588 | 2.177889769 | 7.67E-08 | 1.33E-06 |
| GOBP_REGULATION_OF_NATURAL_KILLER_CELL_ACTIVATION | 0.765665959 | 2.175771484 | 7.89E-08 | 1.36E-06 |
| GOBP_REGULATION_OF_VIRAL_PROCESS | 0.562548233 | 2.02187877 | 7.99E-08 | 1.37E-06 |
| GOBP_NEGATIVE_REGULATION_OF_RESPONSE_TO_BIOTIC_STIMULUS | 0.591205567 | 2.0462036 | 8.74E-08 | 1.50E-06 |
| GOBP_NEGATIVE_REGULATION_OF_PRODUCTION_OF_MOLECULAR_MEDIATOR_OF_IMMUNE_RESPONSE | 0.726878542 | 2.135430083 | 8.88E-08 | 1.52E-06 |
| GOBP_POSITIVE_REGULATION_OF_TYPE_I_INTERFERON_PRODUCTION | 0.703083294 | 2.173012571 | 9.09E-08 | 1.55E-06 |
| GOBP_ANTIGEN_PROCESSING_AND_PRESENTATION_OF_PEPTIDE_ANTIGEN_VIA_MHC_CLASS_IB | 0.909175603 | 2.159358151 | 9.12E-08 | 1.55E-06 |
| GOBP_POSITIVE_REGULATION_OF_INTERLEUKIN_6_PRODUCTION | 0.635242768 | 2.146074188 | 9.31E-08 | 1.57E-06 |
| GOBP_REGULATION_OF_T_HELPER_1_TYPE_IMMUNE_RESPONSE | 0.802207442 | 2.204461545 | 9.36E-08 | 1.58E-06 |
| GOBP_ALPHA_BETA_T_CELL_PROLIFERATION | 0.744978931 | 2.173573884 | 9.57E-08 | 1.60E-06 |
| GOBP_REGULATION_OF_DNA_BINDING_TRANSCRIPTION_FACTOR_ACTIVITY | 0.444373804 | 1.762328656 | 1.03E-07 | 1.72E-06 |
| GOBP_NEGATIVE_REGULATION_OF_INTERLEUKIN_12_PRODUCTION | 0.87401954 | 2.152684752 | 1.10E-07 | 1.83E-06 |
| GOBP_MAINTENANCE_OF_LOCATION | 0.46413478 | 1.803394531 | 1.18E-07 | 1.96E-06 |
| GOBP_VACUOLAR_LOCALIZATION | 0.646628975 | 2.129867579 | 1.19E-07 | 1.97E-06 |
| GOBP_T_HELPER_1_TYPE_IMMUNE_RESPONSE | 0.74213394 | 2.165273251 | 1.20E-07 | 1.97E-06 |
| GOBP_EPIDERMIS_DEVELOPMENT | -0.404730564 | -1.769928225 | 1.26E-07 | 2.06E-06 |
| GOCC_CELL_CELL_JUNCTION | -0.370295145 | -1.687740789 | 1.26E-07 | 2.06E-06 |
| GOBP_POSITIVE_REGULATION_OF_CD4_POSITIVE_ALPHA_BETA_T_CELL_ACTIVATION | 0.760684028 | 2.181406664 | 1.28E-07 | 2.08E-06 |
| GOBP_MAST_CELL_MEDIATED_IMMUNITY | 0.707516114 | 2.181149561 | 1.29E-07 | 2.10E-06 |
| GOBP_T_CELL_MIGRATION | 0.679398363 | 2.154631529 | 1.37E-07 | 2.22E-06 |
| GOBP_REGULATION_OF_ANTIGEN_PROCESSING_AND_PRESENTATION | 0.875104327 | 2.190247977 | 1.43E-07 | 2.30E-06 |
| GOBP_DENDRITIC_CELL_DIFFERENTIATION | 0.739807862 | 2.158486612 | 1.46E-07 | 2.34E-06 |
| GOBP_REGULATION_OF_ENDOPEPTIDASE_ACTIVITY | 0.472902797 | 1.826064394 | 1.50E-07 | 2.40E-06 |
| GOBP_ANTIGEN_PROCESSING_AND_PRESENTATION_VIA_MHC_CLASS_IB | 0.851689382 | 2.217108025 | 1.51E-07 | 2.42E-06 |
| GOBP_REGULATION_OF_LEUKOCYTE_DEGRANULATION | 0.731612733 | 2.151760056 | 1.52E-07 | 2.42E-06 |
| GOBP_T_CELL_ACTIVATION_VIA_T_CELL_RECEPTOR_CONTACT_WITH_ANTIGEN_BOUND_TO_MHC_MOLECULE_ON_ANTIGEN_PRESENTING_CELL | 0.944491683 | 2.033407552 | 1.58E-07 | 2.51E-06 |
| GOBP_INTERMEDIATE_FILAMENT_BASED_PROCESS | -0.605119666 | -2.250354892 | 1.64E-07 | 2.59E-06 |
| GOBP_COLLAGEN_METABOLIC_PROCESS | 0.62737891 | 2.108745987 | 1.70E-07 | 2.69E-06 |
| GOBP_ESTABLISHMENT_OF_RNA_LOCALIZATION | 0.558122698 | 1.997210943 | 1.84E-07 | 2.90E-06 |
| GOBP_MITOTIC_SISTER_CHROMATID_SEGREGATION | 0.527706922 | 1.931028 | 1.85E-07 | 2.90E-06 |
| GOBP_NEGATIVE_REGULATION_OF_TYPE_II_INTERFERON_PRODUCTION | 0.768565873 | 2.169501394 | 1.88E-07 | 2.93E-06 |
| GOBP_DENDRITIC_CELL_ANTIGEN_PROCESSING_AND_PRESENTATION | 0.917299353 | 2.054556369 | 1.91E-07 | 2.97E-06 |
| GOCC_INTERMEDIATE_FILAMENT | -0.523508278 | -2.065124838 | 1.93E-07 | 3.00E-06 |
| GOMF_MHC_PROTEIN_BINDING | 0.830229322 | 2.178414272 | 2.01E-07 | 3.11E-06 |
| GOBP_INTERLEUKIN_17_PRODUCTION | 0.750839553 | 2.125951566 | 2.25E-07 | 3.47E-06 |
| GOBP_POSITIVE_REGULATION_OF_B_CELL_PROLIFERATION | 0.736073734 | 2.140444375 | 2.28E-07 | 3.50E-06 |
| GOBP_IMMUNOLOGICAL_SYNAPSE_FORMATION | 0.897154148 | 2.149647499 | 2.31E-07 | 3.51E-06 |
| GOBP_REGULATION_OF_NEUTROPHIL_MIGRATION | 0.735972077 | 2.140148766 | 2.31E-07 | 3.51E-06 |
| GOBP_PLATELET_ACTIVATION | 0.577607789 | 2.037501064 | 2.29E-07 | 3.51E-06 |
| GOBP_TELOMERE_ORGANIZATION | 0.562229844 | 2.009837193 | 2.30E-07 | 3.51E-06 |
| GOCC_RECEPTOR_COMPLEX | 0.442641171 | 1.744642604 | 2.32E-07 | 3.51E-06 |
| GOBP_INTERLEUKIN_8_PRODUCTION | 0.638880433 | 2.104345417 | 2.55E-07 | 3.85E-06 |
| GOCC_VACUOLAR_LUMEN | 0.548614898 | 1.975500065 | 2.58E-07 | 3.88E-06 |
| GOBP_RAS_PROTEIN_SIGNAL_TRANSDUCTION | 0.45716356 | 1.778816147 | 2.62E-07 | 3.93E-06 |
| GOBP_NEGATIVE_T_CELL_SELECTION | 0.913508875 | 2.046066501 | 2.76E-07 | 4.12E-06 |
| GOBP_POSITIVE_REGULATION_OF_PROTEOLYSIS | 0.457208981 | 1.785376327 | 2.88E-07 | 4.29E-06 |
| GOBP_POSITIVE_REGULATION_OF_PEPTIDASE_ACTIVITY | 0.531062193 | 1.925839099 | 2.99E-07 | 4.44E-06 |
| GOBP_REGULATION_OF_MACROPHAGE_ACTIVATION | 0.725878173 | 2.172146874 | 3.00E-07 | 4.44E-06 |
| GOBP_REGULATION_OF_DNA_RECOMBINATION | 0.590329605 | 2.040459362 | 3.01E-07 | 4.44E-06 |
| GOBP_RESPONSE_TO_INTERFERON_BETA | 0.8113564 | 2.183787402 | 3.06E-07 | 4.51E-06 |
| GOBP_POSITIVE_REGULATION_OF_RESPONSE_TO_CYTOKINE_STIMULUS | 0.688412079 | 2.12766839 | 3.14E-07 | 4.61E-06 |
| GOBP_POSITIVE_REGULATION_OF_PROTEIN_CONTAINING_COMPLEX_ASSEMBLY | 0.525547492 | 1.912303462 | 3.23E-07 | 4.73E-06 |
| GOBP_TOLL_LIKE_RECEPTOR_SIGNALING_PATHWAY | 0.669982675 | 2.124770788 | 3.46E-07 | 5.05E-06 |
| GOBP_POSITIVE_REGULATION_OF_B_CELL_MEDIATED_IMMUNITY | 0.771459786 | 2.144608065 | 3.55E-07 | 5.17E-06 |
| GOBP_REGULATION_OF_B_CELL_MEDIATED_IMMUNITY | 0.68855287 | 2.113855703 | 3.83E-07 | 5.55E-06 |
| GOBP_REGULATION_OF_APOPTOTIC_SIGNALING_PATHWAY | 0.450756355 | 1.762444653 | 3.92E-07 | 5.66E-06 |
| GOBP_POSITIVE_REGULATION_OF_MAPK_CASCADE | 0.424070225 | 1.684217814 | 4.06E-07 | 5.85E-06 |
| GOBP_CD8_POSITIVE_ALPHA_BETA_T_CELL_ACTIVATION | 0.81999492 | 2.165615159 | 4.17E-07 | 5.99E-06 |
| GOBP_MRNA_TRANSPORT | 0.573396431 | 1.984564942 | 4.25E-07 | 6.09E-06 |
| GOBP_NEGATIVE_REGULATION_OF_TUMOR_NECROSIS_FACTOR_SUPERFAMILY_CYTOKINE_PRODUCTION | 0.685766723 | 2.119492417 | 4.27E-07 | 6.09E-06 |
| GOCC_ADHERENS_JUNCTION | -0.477754914 | -1.946413321 | 4.58E-07 | 6.52E-06 |
| GOBP_SOMATIC_DIVERSIFICATION_OF_IMMUNE_RECEPTORS | 0.66098259 | 2.11360118 | 4.64E-07 | 6.59E-06 |
| GOBP_RNA_SPLICING | 0.433801479 | 1.711862985 | 4.99E-07 | 7.06E-06 |
| GOBP_POSITIVE_REGULATION_OF_CD4_POSITIVE_ALPHA_BETA_T_CELL_DIFFERENTIATION | 0.773392224 | 2.142731545 | 5.25E-07 | 7.41E-06 |
| GOBP_NEGATIVE_REGULATION_OF_CHROMOSOME_ORGANIZATION | 0.61492799 | 2.05862836 | 5.63E-07 | 7.92E-06 |
| GOBP_MONOCYTE_CHEMOTAXIS | 0.686417498 | 2.157232325 | 5.77E-07 | 8.09E-06 |
| GOBP_POSITIVE_REGULATION_OF_CHROMOSOME_ORGANIZATION | 0.600829457 | 2.036266109 | 5.84E-07 | 8.18E-06 |
| GOMF_CYTOKINE_ACTIVITY | 0.502700357 | 1.869982866 | 6.13E-07 | 8.55E-06 |
| GOBP_MYELOID_CELL_HOMEOSTASIS | 0.541025847 | 1.948172754 | 6.19E-07 | 8.61E-06 |
| GOBP_MRNA_PROCESSING | 0.428572832 | 1.7006165 | 6.29E-07 | 8.72E-06 |
| GOCC_EARLY_ENDOSOME | 0.436332872 | 1.718609989 | 6.38E-07 | 8.82E-06 |
| GOBP_POSITIVE_REGULATION_OF_CYSTEINE_TYPE_ENDOPEPTIDASE_ACTIVITY | 0.552173733 | 1.951122133 | 6.48E-07 | 8.94E-06 |
| GOBP_RECOMBINATIONAL_REPAIR | 0.537537114 | 1.933949353 | 6.58E-07 | 9.05E-06 |
| GOBP_NUCLEAR_EXPORT | 0.532671934 | 1.917963015 | 7.30E-07 | 1.00E-05 |
| GOBP_PEPTIDYL_TYROSINE_MODIFICATION | 0.444506903 | 1.742341897 | 7.40E-07 | 1.01E-05 |
| GOMF_DOUBLE_STRANDED_RNA_BINDING | 0.658036956 | 2.091190989 | 7.65E-07 | 1.04E-05 |
| GOBP_B_CELL_HOMEOSTASIS | 0.762412238 | 2.11945647 | 7.78E-07 | 1.06E-05 |
| GOCC_LATERAL_PLASMA_MEMBRANE | -0.635120478 | -2.218569723 | 8.10E-07 | 1.10E-05 |
| GOCC_DESMOSOME | -0.774115526 | -2.255951079 | 8.17E-07 | 1.10E-05 |
| GOBP_NEGATIVE_REGULATION_OF_CYTOKINE_PRODUCTION_INVOLVED_IN_IMMUNE_RESPONSE | 0.75437447 | 2.110325408 | 8.27E-07 | 1.11E-05 |
| GOBP_NEGATIVE_REGULATION_OF_T_CELL_PROLIFERATION | 0.657020667 | 2.087602472 | 8.81E-07 | 1.18E-05 |
| GOBP_TELOMERE_MAINTENANCE | 0.555104703 | 1.970010352 | 8.89E-07 | 1.19E-05 |
| GOBP_POSITIVE_REGULATION_OF_NATURAL_KILLER_CELL_ACTIVATION | 0.816589465 | 2.127080584 | 9.59E-07 | 1.28E-05 |
| GOBP_INTERLEUKIN_4_PRODUCTION | 0.769108126 | 2.120895572 | 1.00E-06 | 1.33E-05 |
| GOBP_REGULATION_OF_MITOTIC_CELL_CYCLE_PHASE_TRANSITION | 0.447089039 | 1.737163344 | 1.00E-06 | 1.33E-05 |
| GOBP_REGULATION_OF_PEPTIDASE_ACTIVITY | 0.435884553 | 1.713697153 | 1.01E-06 | 1.33E-05 |
| GOBP_NUCLEAR_TRANSPORT | 0.461130243 | 1.783214557 | 1.01E-06 | 1.34E-05 |
| GOBP_REGULATION_OF_NEUROINFLAMMATORY_RESPONSE | 0.768085179 | 2.11807469 | 1.08E-06 | 1.43E-05 |
| GOBP_REGULATION_OF_B_CELL_RECEPTOR_SIGNALING_PATHWAY | 0.828130601 | 2.13100159 | 1.21E-06 | 1.59E-05 |
| GOBP_MITOTIC_CELL_CYCLE_PHASE_TRANSITION | 0.421503572 | 1.67344961 | 1.24E-06 | 1.63E-05 |
| GOBP_RNA_EXPORT_FROM_NUCLEUS | 0.626236378 | 2.052086637 | 1.26E-06 | 1.65E-05 |
| GOBP_POSITIVE_REGULATION_OF_ERK1_AND_ERK2_CASCADE | 0.502535562 | 1.852863259 | 1.35E-06 | 1.75E-05 |
| GOBP_ERK1_AND_ERK2_CASCADE | 0.45971755 | 1.777751597 | 1.37E-06 | 1.79E-05 |
| GOBP_REGULATION_OF_NIK_NF_KAPPAB_SIGNALING | 0.600007126 | 2.02375328 | 1.38E-06 | 1.79E-05 |
| GOBP_SENSORY_PERCEPTION_OF_CHEMICAL_STIMULUS | -0.46669741 | -1.896051141 | 1.45E-06 | 1.87E-05 |
| GOBP_POSITIVE_REGULATION_OF_INTERLEUKIN_17_PRODUCTION | 0.806747596 | 2.130628838 | 1.48E-06 | 1.91E-05 |
| GOBP_NEGATIVE_REGULATION_OF_VIRAL_PROCESS | 0.614709303 | 2.03315308 | 1.49E-06 | 1.92E-05 |
| GOBP_MESONEPHROS_DEVELOPMENT | -0.550689732 | -2.111001166 | 1.51E-06 | 1.94E-05 |
| GOBP_REGULATION_OF_MAST_CELL_ACTIVATION | 0.710193881 | 2.047877989 | 1.51E-06 | 1.94E-05 |
| GOBP_NEGATIVE_REGULATION_OF_NATURAL_KILLER_CELL_MEDIATED_IMMUNITY | 0.832191352 | 2.112678354 | 1.54E-06 | 1.97E-05 |
| GOCC_CLATHRIN_COATED_VESICLE_MEMBRANE | 0.551271306 | 1.929220092 | 1.56E-06 | 1.98E-05 |
| GOMF_HISTONE_BINDING | 0.485086306 | 1.816898779 | 1.60E-06 | 2.03E-05 |
| GOBP_REGULATION_OF_PATTERN_RECOGNITION_RECEPTOR_SIGNALING_PATHWAY | 0.606747962 | 2.045561387 | 1.64E-06 | 2.08E-05 |
| GOBP_CELL_JUNCTION_ASSEMBLY | -0.36649467 | -1.635603065 | 1.66E-06 | 2.10E-05 |
| GOMF_CYTOKINE_BINDING | 0.548203504 | 1.935045475 | 1.90E-06 | 2.39E-05 |
| GOBP_CHROMOSOME_SEPARATION | 0.618496041 | 2.021770538 | 2.32E-06 | 2.91E-05 |
| GOBP_INTRINSIC_APOPTOTIC_SIGNALING_PATHWAY | 0.464449513 | 1.7827831 | 2.38E-06 | 2.99E-05 |
| GOBP_EXOCYTOSIS | 0.439923986 | 1.712173919 | 2.47E-06 | 3.09E-05 |
| GOMF_SINGLE_STRANDED_DNA_BINDING | 0.578615032 | 1.984616732 | 2.54E-06 | 3.16E-05 |
| GOBP_VIRAL_GENOME_REPLICATION | 0.55855431 | 1.933195328 | 2.57E-06 | 3.20E-05 |
| GOBP_REGULATION_OF_SMALL_GTPASE_MEDIATED_SIGNAL_TRANSDUCTION | 0.445383639 | 1.718453347 | 2.65E-06 | 3.28E-05 |
| GOBP_INTERFERON_BETA_PRODUCTION | 0.682160368 | 2.060907603 | 2.66E-06 | 3.29E-05 |
| GOBP_REGULATED_EXOCYTOSIS | 0.485753027 | 1.811168689 | 2.73E-06 | 3.37E-05 |
| GOBP_CHEMOKINE_PRODUCTION | 0.598447312 | 2.021766155 | 2.81E-06 | 3.46E-05 |
| GOBP_BONE_RESORPTION | 0.643503029 | 2.036380215 | 2.86E-06 | 3.52E-05 |
| GOBP_POSITIVE_REGULATION_OF_INTERLEUKIN_2_PRODUCTION | 0.751529348 | 2.087304728 | 2.88E-06 | 3.53E-05 |
| GOBP_REGULATION_OF_T_HELPER_CELL_DIFFERENTIATION | 0.724466167 | 2.058695191 | 2.90E-06 | 3.53E-05 |
| GOBP_POSITIVE_REGULATION_OF_INTERLEUKIN_10_PRODUCTION | 0.724325807 | 2.058296334 | 2.90E-06 | 3.53E-05 |
| GOBP_NEGATIVE_REGULATION_OF_NF_KAPPAB_TRANSCRIPTION_FACTOR_ACTIVITY | 0.610368912 | 2.01256336 | 2.96E-06 | 3.60E-05 |
| GOBP_REGULATION_OF_LYMPHOCYTE_MIGRATION | 0.658186709 | 2.073310238 | 3.27E-06 | 3.95E-05 |
| GOMF_INTEGRIN_BINDING | 0.533422242 | 1.904086431 | 3.26E-06 | 3.95E-05 |
| GOCC_TIGHT_JUNCTION | -0.507843314 | -2.001800554 | 3.45E-06 | 4.17E-05 |
| GOBP_REGULATION_OF_PEPTIDYL_TYROSINE_PHOSPHORYLATION | 0.473707381 | 1.792459649 | 3.49E-06 | 4.20E-05 |
| GOBP_RNA_SPLICING_VIA_TRANSESTERIFICATION_REACTIONS | 0.455866715 | 1.745787152 | 3.54E-06 | 4.25E-05 |
| GOBP_REGULATION_OF_DENDRITIC_CELL_ANTIGEN_PROCESSING_AND_PRESENTATION | 0.921951698 | 1.984880945 | 3.64E-06 | 4.35E-05 |
| GOBP_NEGATIVE_REGULATION_OF_T_CELL_RECEPTOR_SIGNALING_PATHWAY | 0.793960485 | 2.096857944 | 3.73E-06 | 4.46E-05 |
| GOBP_T_HELPER_17_TYPE_IMMUNE_RESPONSE | 0.720953738 | 2.04871402 | 3.80E-06 | 4.52E-05 |
| GOBP_POSITIVE_REGULATION_OF_LYMPHOCYTE_MIGRATION | 0.718917994 | 2.04068823 | 3.88E-06 | 4.61E-05 |
| GOBP_CALCIUM_ION_TRANSPORT | 0.415873714 | 1.648129535 | 4.31E-06 | 5.12E-05 |
| GOBP_CELLULAR_RESPONSE_TO_INTERLEUKIN_1 | 0.581625904 | 1.978523147 | 4.41E-06 | 5.20E-05 |
| GOCC_SPECIFIC_GRANULE | 0.514456748 | 1.850910884 | 4.40E-06 | 5.20E-05 |
| GOCC_SITE_OF_DNA_DAMAGE | 0.59377261 | 2.001816897 | 4.57E-06 | 5.38E-05 |
| GOBP_IMMUNE_RESPONSE_REGULATING_CELL_SURFACE_RECEPTOR_SIGNALING_PATHWAY_INVOLVED_IN_PHAGOCYTOSIS | 0.79042567 | 2.08752246 | 4.60E-06 | 5.40E-05 |
| GOBP_LYMPHOCYTE_CHEMOTAXIS | 0.69501193 | 2.079781495 | 4.62E-06 | 5.41E-05 |
| GOBP_T_CELL_LINEAGE_COMMITMENT | 0.757845154 | 2.082554226 | 4.65E-06 | 5.43E-05 |
| GOBP_MAINTENANCE_OF_LOCATION_IN_CELL | 0.481682432 | 1.795991153 | 4.68E-06 | 5.45E-05 |
| GOBP_REGULATION_OF_CALCIUM_ION_TRANSPORT | 0.469224536 | 1.775647723 | 4.79E-06 | 5.57E-05 |
| GOBP_T_CELL_CYTOKINE_PRODUCTION | 0.715970754 | 2.032322329 | 4.83E-06 | 5.60E-05 |
| GOMF_PROTEASE_BINDING | 0.544636804 | 1.921196507 | 4.84E-06 | 5.60E-05 |
| GOBP_POSITIVE_REGULATION_OF_INTERLEUKIN_4_PRODUCTION | 0.789333918 | 2.084639132 | 5.06E-06 | 5.84E-05 |
| GOBP_RESPONSE_TO_INTERLEUKIN_1 | 0.555403761 | 1.939511076 | 5.15E-06 | 5.93E-05 |
| GOBP_POSITIVE_REGULATION_OF_NITRIC_OXIDE_SYNTHASE_BIOSYNTHETIC_PROCESS | 0.845260489 | 2.053508461 | 5.20E-06 | 5.97E-05 |
| GOBP_POSITIVE_REGULATION_OF_NEUTROPHIL_MIGRATION | 0.747468668 | 2.07090871 | 5.52E-06 | 6.33E-05 |
| GOBP_POSITIVE_REGULATION_OF_REGULATORY_T_CELL_DIFFERENTIATION | 0.808114975 | 2.079496031 | 5.58E-06 | 6.38E-05 |
| GOCC_PHAGOCYTIC_CUP | 0.788084778 | 2.081340141 | 5.76E-06 | 6.57E-05 |
| GOBP_THYMIC_T_CELL_SELECTION | 0.815517411 | 2.122945567 | 5.78E-06 | 6.57E-05 |
| GOBP_NEGATIVE_REGULATION_OF_INTRACELLULAR_SIGNAL_TRANSDUCTION | 0.405302672 | 1.610318225 | 5.79E-06 | 6.58E-05 |
| GOBP_RESPONSE_TO_INTERLEUKIN_4 | 0.738854274 | 2.053966861 | 5.97E-06 | 6.76E-05 |
| GOCC_FICOLIN_1_RICH_GRANULE_MEMBRANE | 0.656674738 | 2.029578105 | 6.10E-06 | 6.89E-05 |
| GOBP_REGULATION_OF_DEFENSE_RESPONSE_TO_VIRUS_BY_HOST | 0.728991254 | 2.039317114 | 6.25E-06 | 7.03E-05 |
| GOCC_I_BAND | -0.480176354 | -1.896969896 | 6.24E-06 | 7.03E-05 |
| GOBP_POSITIVE_REGULATION_OF_IMMUNOGLOBULIN_PRODUCTION | 0.687919769 | 2.023253851 | 6.28E-06 | 7.04E-05 |
| GOBP_REGULATION_OF_CELL_CYCLE_PHASE_TRANSITION | 0.421207911 | 1.664612067 | 6.29E-06 | 7.04E-05 |
| GOBP_MUSCLE_TISSUE_DEVELOPMENT | -0.356981506 | -1.592977057 | 6.35E-06 | 7.10E-05 |
| GOBP_ACUTE_INFLAMMATORY_RESPONSE_TO_ANTIGENIC_STIMULUS | 0.792439196 | 2.064173126 | 6.38E-06 | 7.11E-05 |
| GOBP_NEPHRON_EPITHELIUM_DEVELOPMENT | -0.511511608 | -1.990317876 | 6.44E-06 | 7.16E-05 |
| GOBP_POSITIVE_REGULATION_OF_T_HELPER_1_TYPE_IMMUNE_RESPONSE | 0.831524066 | 2.048019632 | 6.53E-06 | 7.24E-05 |
| GOMF_SH3_DOMAIN_BINDING | 0.547619918 | 1.903396819 | 6.56E-06 | 7.26E-05 |
| GOBP_CELL_SURFACE_PATTERN_RECOGNITION_RECEPTOR_SIGNALING_PATHWAY | 0.666159396 | 2.024868951 | 6.61E-06 | 7.30E-05 |
| GOBP_ENDODERMAL_CELL_DIFFERENTIATION | 0.683450439 | 2.001028298 | 6.68E-06 | 7.36E-05 |
| GOCC_SECONDARY_LYSOSOME | 0.830069415 | 2.044436868 | 6.77E-06 | 7.44E-05 |
| GOBP_MONOCARBOXYLIC_ACID_CATABOLIC_PROCESS | -0.492668321 | -1.935646564 | 6.85E-06 | 7.51E-05 |
| GOCC_AZUROPHIL_GRANULE | 0.523714847 | 1.865212133 | 7.26E-06 | 7.94E-05 |
| GOMF_CATALYTIC_ACTIVITY_ACTING_ON_DNA | 0.465793209 | 1.756652321 | 7.28E-06 | 7.94E-05 |
| GOCC_CONTRACTILE_FIBER | -0.415857382 | -1.738958067 | 7.43E-06 | 8.09E-05 |
| GOBP_MATURE_B_CELL_DIFFERENTIATION | 0.727587951 | 2.053829256 | 7.51E-06 | 8.16E-05 |
| GOBP_SENSORY_ORGAN_MORPHOGENESIS | -0.405272165 | -1.713494621 | 7.78E-06 | 8.43E-05 |
| GOCC_SITE_OF_DOUBLE_STRAND_BREAK | 0.630817319 | 2.004344551 | 7.89E-06 | 8.54E-05 |
| GOBP_FC_RECEPTOR_SIGNALING_PATHWAY | 0.669379121 | 2.011423669 | 7.93E-06 | 8.55E-05 |
| GOBP_HUMORAL_IMMUNE_RESPONSE_MEDIATED_BY_CIRCULATING_IMMUNOGLOBULIN | 0.687688829 | 1.982983596 | 7.94E-06 | 8.55E-05 |
| GOBP_CHRONIC_INFLAMMATORY_RESPONSE | 0.817794263 | 2.076128565 | 7.97E-06 | 8.56E-05 |
| GOBP_REGULATION_OF_RESPONSE_TO_DNA_DAMAGE_STIMULUS | 0.452856126 | 1.738282044 | 8.05E-06 | 8.63E-05 |
| GOBP_CAMERA_TYPE_EYE_MORPHOGENESIS | -0.489748105 | -1.924173316 | 8.14E-06 | 8.71E-05 |
| GOBP_EXTERNAL_ENCAPSULATING_STRUCTURE_ORGANIZATION | 0.434369076 | 1.675191726 | 8.17E-06 | 8.72E-05 |
| GOBP_DEFENSE_RESPONSE_TO_GRAM_POSITIVE_BACTERIUM | 0.596638181 | 1.978164131 | 8.23E-06 | 8.77E-05 |
| GOBP_REGULATION_OF_SISTER_CHROMATID_SEGREGATION | 0.567248465 | 1.924106326 | 8.59E-06 | 9.12E-05 |
| GOCC_SPLICEOSOMAL_COMPLEX | 0.499220007 | 1.815371342 | 8.63E-06 | 9.15E-05 |
| GOBP_ENDOLYSOSOMAL_TOLL_LIKE_RECEPTOR_SIGNALING_PATHWAY | 0.710883339 | 2.020097248 | 8.65E-06 | 9.15E-05 |
| GOBP_REGULATION_OF_TOLERANCE_INDUCTION | 0.822394442 | 2.058323456 | 8.81E-06 | 9.30E-05 |
| GOMF_HELICASE_ACTIVITY | 0.520893274 | 1.863987528 | 9.09E-06 | 9.57E-05 |
| GOCC_KERATIN_FILAMENT | -0.60663532 | -2.124728405 | 9.15E-06 | 9.62E-05 |
| GOBP_EOSINOPHIL_MIGRATION | 0.775873307 | 2.069864524 | 9.22E-06 | 9.67E-05 |
| GOBP_DIGESTIVE_SYSTEM_DEVELOPMENT | -0.484690473 | -1.928598196 | 9.56E-06 | 0.000100009 |
| GOBP_NEGATIVE_REGULATION_OF_MITOTIC_CELL_CYCLE_PHASE_TRANSITION | 0.500647192 | 1.812506253 | 9.62E-06 | 0.000100464 |
| GOBP_CELL_ADHESION_MEDIATED_BY_INTEGRIN | 0.59846974 | 1.971240612 | 9.89E-06 | 0.000103073 |
| GOBP_NEGATIVE_REGULATION_OF_HEMOPOIESIS | 0.560580217 | 1.911743222 | 1.00E-05 | 0.000104045 |
| GOMF_UBIQUITIN_LIKE_PROTEIN_LIGASE_BINDING | 0.440747524 | 1.699860726 | 1.02E-05 | 0.000105641 |
| GOCC_MIDBODY | 0.490471919 | 1.784674388 | 1.02E-05 | 0.000105806 |
| GOBP_T_CELL_HOMEOSTASIS | 0.690175913 | 2.006977133 | 1.03E-05 | 0.000106572 |
| GOMF_ATP_DEPENDENT_ACTIVITY_ACTING_ON_DNA | 0.55120867 | 1.915302258 | 1.06E-05 | 0.000109567 |
| GOBP_RESPONSE_TO_PROTOZOAN | 0.775012418 | 2.033532265 | 1.07E-05 | 0.000109822 |
| GOBP_ORGANELLE_FISSION | 0.39955882 | 1.586405509 | 1.10E-05 | 0.000113008 |
| GOCC_CONDENSED_CHROMOSOME_CENTROMERIC_REGION | 0.511406751 | 1.839381579 | 1.11E-05 | 0.000113994 |
| GOBP_REGULATION_OF_LYMPHOCYTE_APOPTOTIC_PROCESS | 0.646643695 | 1.998575262 | 1.14E-05 | 0.000116418 |
| GOCC_RIBONUCLEOPROTEIN_GRANULE | 0.461059802 | 1.744627248 | 1.15E-05 | 0.000117276 |
| GOBP_REGULATION_OF_TELOMERE_MAINTENANCE | 0.576954304 | 1.949155189 | 1.17E-05 | 0.000119204 |
| GOCC_NUCLEAR_SPECK | 0.414225838 | 1.632348427 | 1.17E-05 | 0.000119204 |
| GOBP_LYMPHOCYTE_APOPTOTIC_PROCESS | 0.597260909 | 1.952356085 | 1.20E-05 | 0.000121791 |
| GOBP_CELLULAR_EXTRAVASATION | 0.606229427 | 1.961239356 | 1.23E-05 | 0.000124477 |
| GOBP_NEGATIVE_REGULATION_OF_T_CELL_MEDIATED_IMMUNITY | 0.771221597 | 2.057454752 | 1.25E-05 | 0.000126643 |
| GOCC_PHAGOCYTIC_VESICLE_MEMBRANE | 0.603251439 | 1.953060424 | 1.26E-05 | 0.00012685 |
| GOMF_GLYCOSAMINOGLYCAN_BINDING | 0.472110692 | 1.758461567 | 1.27E-05 | 0.000127813 |
| GOBP_ACTIVATED_T_CELL_PROLIFERATION | 0.677758982 | 1.993369768 | 1.28E-05 | 0.000128497 |
| GOBP_POSITIVE_REGULATION_OF_APOPTOTIC_SIGNALING_PATHWAY | 0.528290336 | 1.848796265 | 1.28E-05 | 0.000128497 |
| GOBP_MORPHOGENESIS_OF_EMBRYONIC_EPITHELIUM | -0.454386517 | -1.814456182 | 1.32E-05 | 0.00013133 |
| GOBP_POSITIVE_REGULATION_OF_LEUKOCYTE_APOPTOTIC_PROCESS | 0.777209186 | 2.052617589 | 1.32E-05 | 0.000131621 |
| GOBP_REGULATION_OF_MYELOID_CELL_DIFFERENTIATION | 0.487823213 | 1.781130739 | 1.34E-05 | 0.000132857 |
| GOCC_CORNIFIED_ENVELOPE | -0.622132896 | -2.124871579 | 1.35E-05 | 0.000133543 |
| GOCC_CATALYTIC_STEP_2_SPLICEOSOME | 0.58593828 | 1.937992826 | 1.35E-05 | 0.000133707 |
| GOBP_REGULATION_OF_RECEPTOR_SIGNALING_PATHWAY_VIA_STAT | 0.547294902 | 1.865349456 | 1.35E-05 | 0.000133707 |
| GOBP_DEFENSE_RESPONSE_TO_BACTERIUM | 0.45164992 | 1.714933761 | 1.37E-05 | 0.000134896 |
| GOMF_EXTRACELLULAR_MATRIX_STRUCTURAL_CONSTITUENT | 0.508432874 | 1.830809154 | 1.38E-05 | 0.000135393 |
| GOBP_MACROPHAGE_DIFFERENTIATION | 0.657221114 | 1.997700004 | 1.40E-05 | 0.000137061 |
| GOCC_COLLAGEN_TRIMER | 0.600930201 | 1.950402077 | 1.40E-05 | 0.000137163 |
| GOCC_AZUROPHIL_GRANULE_LUMEN | 0.593609757 | 1.955232792 | 1.44E-05 | 0.000140662 |
| GOCC_CLATHRIN_COATED_ENDOCYTIC_VESICLE | 0.572899842 | 1.908735064 | 1.51E-05 | 0.000147048 |
| GOBP_POSITIVE_REGULATION_OF_INTERLEUKIN_8_PRODUCTION | 0.647840753 | 2.035995614 | 1.55E-05 | 0.000151381 |
| GOBP_HYPERSENSITIVITY | 0.897029681 | 1.962016546 | 1.56E-05 | 0.000151537 |
| GOBP_POSITIVE_REGULATION_OF_T_CELL_APOPTOTIC_PROCESS | 0.870659708 | 1.950093437 | 1.56E-05 | 0.000151537 |
| GOBP_NEPHRON_DEVELOPMENT | -0.444732616 | -1.778425402 | 1.56E-05 | 0.000151537 |
| GOBP_NUCLEOBASE_CONTAINING_COMPOUND_TRANSPORT | 0.478185033 | 1.767419295 | 1.57E-05 | 0.00015207 |
| GOBP_CELL_SURFACE_TOLL_LIKE_RECEPTOR_SIGNALING_PATHWAY | 0.676016574 | 1.988245138 | 1.58E-05 | 0.00015236 |
| GOCC_SPINDLE | 0.413330239 | 1.629924693 | 1.64E-05 | 0.000158049 |
| GOBP_SOMATIC_DIVERSIFICATION_OF_IMMUNOGLOBULINS | 0.641017741 | 1.981187181 | 1.70E-05 | 0.000163648 |
| GOBP_MUSCLE_CONTRACTION | -0.37140423 | -1.62037571 | 1.80E-05 | 0.00017213 |
| GOCC_INTERMEDIATE_FILAMENT_CYTOSKELETON | -0.437924905 | -1.778422119 | 1.82E-05 | 0.000174076 |
| GOCC_CLATHRIN_COATED_ENDOCYTIC_VESICLE_MEMBRANE | 0.616333394 | 1.958660266 | 1.91E-05 | 0.00018234 |
| GOBP_NITRIC_OXIDE_SYNTHASE_BIOSYNTHETIC_PROCESS | 0.812782271 | 2.034265709 | 2.03E-05 | 0.000193378 |
| GOCC_ER_TO_GOLGI_TRANSPORT_VESICLE_MEMBRANE | 0.642358298 | 1.980279305 | 2.04E-05 | 0.0001939 |
| GOBP_CELL_CELL_JUNCTION_ORGANIZATION | -0.418597955 | -1.748221688 | 2.06E-05 | 0.000195834 |
| GOBP_REGULATION_OF_CELL_ADHESION_MEDIATED_BY_INTEGRIN | 0.658456642 | 1.934419633 | 2.07E-05 | 0.000195841 |
| GOBP_OSTEOCLAST_DIFFERENTIATION | 0.564722621 | 1.91681798 | 2.08E-05 | 0.000197142 |
| GOBP_REGULATION_OF_VASCULATURE_DEVELOPMENT | 0.440250305 | 1.681784707 | 2.18E-05 | 0.000205434 |
| GOCC_PEPTIDASE_INHIBITOR_COMPLEX | 0.903358068 | 1.944850496 | 2.20E-05 | 0.00020687 |
| GOMF_PEPTIDE_BINDING | 0.432632604 | 1.667203719 | 2.20E-05 | 0.00020687 |
| GOBP_REGULATION_OF_VENTRICULAR_CARDIAC_MUSCLE_CELL_ACTION_POTENTIAL | -0.852950913 | -2.068674137 | 2.22E-05 | 0.000207944 |
| GOBP_RENAL_SYSTEM_DEVELOPMENT | -0.365432083 | -1.591853835 | 2.24E-05 | 0.000209954 |
| GOCC_NUCLEAR_CHROMOSOME | 0.468216991 | 1.733938708 | 2.27E-05 | 0.000211894 |
| GOBP_GRANULOCYTE_ACTIVATION | 0.665839132 | 1.949465342 | 2.38E-05 | 0.000221613 |
| GOMF_CARBOHYDRATE_BINDING | 0.453609844 | 1.71070503 | 2.40E-05 | 0.000223325 |
| GOBP_MRNA_EXPORT_FROM_NUCLEUS | 0.633682818 | 1.968744743 | 2.44E-05 | 0.000227193 |
| GOBP_POSITIVE_REGULATION_OF_SMOOTH_MUSCLE_CELL_PROLIFERATION | 0.575603367 | 1.903810065 | 2.51E-05 | 0.000232322 |
| GOCC_BASAL_PART_OF_CELL | -0.377564812 | -1.615264328 | 2.51E-05 | 0.000232322 |
| GOBP_REGULATION_OF_SODIUM_ION_TRANSMEMBRANE_TRANSPORT | -0.578120074 | -2.027355419 | 2.59E-05 | 0.000238853 |
| GOBP_REGULATION_OF_ACUTE_INFLAMMATORY_RESPONSE_TO_ANTIGENIC_STIMULUS | 0.871663246 | 1.992991979 | 2.59E-05 | 0.000238856 |
| GOBP_REGULATION_OF_PROTEIN_CONTAINING_COMPLEX_ASSEMBLY | 0.408993493 | 1.60924928 | 2.64E-05 | 0.000242864 |
| GOBP_NEPHRON_TUBULE_FORMATION | -0.780480304 | -2.177125438 | 2.68E-05 | 0.000246423 |
| GOBP_POSITIVE_REGULATION_OF_TOLERANCE_INDUCTION | 0.861324662 | 1.929184911 | 2.73E-05 | 0.000250679 |
| GOMF_PROTEIN_PHOSPHORYLATED_AMINO_ACID_BINDING | 0.631587787 | 1.962235836 | 2.75E-05 | 0.000251457 |
| GOBP_MACROPHAGE_CYTOKINE_PRODUCTION | 0.705673244 | 1.974086129 | 2.76E-05 | 0.000251463 |
| GOBP_DNA_CONFORMATION_CHANGE | 0.579524277 | 1.921422689 | 2.75E-05 | 0.000251463 |
| GOCC_CLATHRIN_COATED_VESICLE | 0.460778536 | 1.702703018 | 2.81E-05 | 0.00025539 |
| GOBP_ENDODERM_FORMATION | 0.640104453 | 1.958769171 | 2.81E-05 | 0.000255492 |
| GOBP_REGULATION_OF_TYPE_2_IMMUNE_RESPONSE | 0.717900829 | 1.993904561 | 2.89E-05 | 0.000262215 |
| GOMF_T_CELL_RECEPTOR_BINDING | 0.899331449 | 1.936181538 | 2.90E-05 | 0.000262384 |
| GOBP_NEGATIVE_REGULATION_OF_PHOSPHORUS_METABOLIC_PROCESS | 0.402387159 | 1.585382788 | 2.91E-05 | 0.000262587 |
| GOBP_IMMUNE_RESPONSE_INHIBITING_SIGNAL_TRANSDUCTION | 0.898834159 | 1.935110918 | 2.95E-05 | 0.000266111 |
| GOBP_INTRINSIC_APOPTOTIC_SIGNALING_PATHWAY_IN_RESPONSE_TO_DNA_DAMAGE | 0.560462269 | 1.902357218 | 3.08E-05 | 0.000276874 |
| GOBP_POSITIVE_REGULATION_OF_LEUKOCYTE_ADHESION_TO_VASCULAR_ENDOTHELIAL_CELL | 0.767638712 | 1.999571963 | 3.10E-05 | 0.000278226 |
| GOBP_POSITIVE_REGULATION_OF_PATTERN_RECOGNITION_RECEPTOR_SIGNALING_PATHWAY | 0.692978718 | 1.96921819 | 3.13E-05 | 0.000280461 |
| GOBP_DETECTION_OF_CHEMICAL_STIMULUS | -0.440705295 | -1.774989703 | 3.14E-05 | 0.000281361 |
| GOBP_CD4_POSITIVE_ALPHA_BETA_T_CELL_CYTOKINE_PRODUCTION | 0.805860137 | 2.0169407 | 3.18E-05 | 0.000283637 |
| GOBP_CELL_CYCLE_CHECKPOINT_SIGNALING | 0.481270856 | 1.75010077 | 3.18E-05 | 0.000283637 |
| GOBP_POSITIVE_REGULATION_OF_PEPTIDYL_TYROSINE_PHOSPHORYLATION | 0.481193614 | 1.749973599 | 3.20E-05 | 0.00028478 |
| GOMF_ATP_HYDROLYSIS_ACTIVITY | 0.412088082 | 1.61145278 | 3.21E-05 | 0.000284814 |
| GOBP_POSITIVE_REGULATION_OF_GTPASE_ACTIVITY | 0.448440177 | 1.69556012 | 3.25E-05 | 0.000288031 |
| GOBP_POSITIVE_THYMIC_T_CELL_SELECTION | 0.85889358 | 2.002569546 | 3.34E-05 | 0.000294766 |
| GOBP_SUPEROXIDE_ANION_GENERATION | 0.689700451 | 1.968052841 | 3.33E-05 | 0.000294766 |
| GOBP_REGULATION_OF_DENDRITIC_CELL_CHEMOTAXIS | 0.856515583 | 1.918413591 | 3.40E-05 | 0.000299397 |
| GOBP_EXTRINSIC_APOPTOTIC_SIGNALING_PATHWAY | 0.461946933 | 1.720604809 | 3.40E-05 | 0.000299397 |
| GOCC_COATED_VESICLE_MEMBRANE | 0.478429746 | 1.746833491 | 3.41E-05 | 0.000299401 |
| GOBP_NEGATIVE_REGULATION_OF_LEUKOCYTE_APOPTOTIC_PROCESS | 0.636284268 | 1.947079109 | 3.43E-05 | 0.000301028 |
| GOBP_LIPOPOLYSACCHARIDE_MEDIATED_SIGNALING_PATHWAY | 0.62487582 | 1.968379819 | 3.46E-05 | 0.000302301 |
| GOBP_ENTRY_INTO_HOST | 0.495814511 | 1.776277384 | 3.46E-05 | 0.000302301 |
| GOBP_NEGATIVE_REGULATION_OF_CELL_CYCLE_PROCESS | 0.429845763 | 1.650121925 | 3.60E-05 | 0.000313983 |
| GOBP_REGULATION_OF_T_CELL_MIGRATION | 0.669671063 | 1.953853289 | 3.74E-05 | 0.000325628 |
| GOBP_MUSCLE_SYSTEM_PROCESS | -0.33578193 | -1.501961695 | 3.76E-05 | 0.000326543 |
| GOBP_TYPE_2_IMMUNE_RESPONSE | 0.689616248 | 1.959663154 | 3.86E-05 | 0.000334749 |
| GOBP_REGULATION_OF_CD8_POSITIVE_ALPHA_BETA_T_CELL_ACTIVATION | 0.804863511 | 1.98235546 | 3.87E-05 | 0.000335438 |
| GOBP_RIBONUCLEOPROTEIN_COMPLEX_BIOGENESIS | 0.399572627 | 1.576789437 | 4.03E-05 | 0.000348486 |
| GOCC_APICAL_PART_OF_CELL | -0.344709085 | -1.53899481 | 4.05E-05 | 0.000349193 |
| GOBP_HISTONE_MODIFICATION | 0.39355916 | 1.563667666 | 4.06E-05 | 0.000349633 |
| GOMF_NUCLEOTIDE_RECEPTOR_ACTIVITY | 0.801031485 | 2.004855347 | 4.12E-05 | 0.000353284 |
| GOCC_LYSOSOMAL_LUMEN | 0.563100339 | 1.898409853 | 4.11E-05 | 0.000353284 |
| GOBP_DNA_TEMPLATED_DNA_REPLICATION | 0.484668011 | 1.745117887 | 4.12E-05 | 0.000353284 |
| GOBP_BODY_FLUID_SECRETION | -0.524190558 | -1.954073248 | 4.26E-05 | 0.000364146 |
| GOBP_REGULATION_OF_MYELOID_LEUKOCYTE_DIFFERENTIATION | 0.528039089 | 1.827580026 | 4.28E-05 | 0.000364146 |
| GOBP_ACTIN_POLYMERIZATION_OR_DEPOLYMERIZATION | 0.477536831 | 1.737265336 | 4.27E-05 | 0.000364146 |
| GOBP_SODIUM_ION_TRANSPORT | -0.395176494 | -1.67359143 | 4.28E-05 | 0.000364146 |
| GOBP_VESICLE_ORGANIZATION | 0.412432018 | 1.60751443 | 4.31E-05 | 0.000366227 |
| GOBP_T_HELPER_CELL_LINEAGE_COMMITMENT | 0.802023294 | 1.975360087 | 4.40E-05 | 0.000372668 |
| GOBP_EMBRYONIC_ORGAN_DEVELOPMENT | -0.332376225 | -1.489832645 | 4.47E-05 | 0.000377992 |
| GOBP_POSITIVE_REGULATION_OF_T_CELL_MIGRATION | 0.717346463 | 1.987453259 | 4.53E-05 | 0.000382331 |
| GOBP_REGULATION_OF_VIRAL_GENOME_REPLICATION | 0.575989282 | 1.880213276 | 4.54E-05 | 0.000382983 |
| GOBP_TUMOR_NECROSIS_FACTOR_MEDIATED_SIGNALING_PATHWAY | 0.553867237 | 1.877106841 | 4.58E-05 | 0.000385477 |
| GOBP_CALCIUM_ION_TRANSMEMBRANE_IMPORT_INTO_CYTOSOL | 0.474239126 | 1.725608316 | 4.59E-05 | 0.000385753 |
| GOMF_CHEMOKINE_BINDING | 0.783696031 | 2.040108515 | 4.64E-05 | 0.000389368 |
| GOBP_REGULATION_OF_TELOMERE_MAINTENANCE_VIA_TELOMERE_LENGTHENING | 0.63704063 | 1.936359075 | 4.75E-05 | 0.000397523 |
| GOBP_CD4_POSITIVE_OR_CD8_POSITIVE_ALPHA_BETA_T_CELL_LINEAGE_COMMITMENT | 0.751717016 | 1.972408144 | 4.78E-05 | 0.000399848 |
| GOMF_PHOSPHOTYROSINE_RESIDUE_BINDING | 0.64719678 | 1.901340311 | 4.84E-05 | 0.00040406 |
| GOBP_REGULATION_OF_NEUTROPHIL_CHEMOTAXIS | 0.716621782 | 1.985445483 | 4.90E-05 | 0.000407837 |
| GOBP_NEGATIVE_REGULATION_OF_DNA_BINDING_TRANSCRIPTION_FACTOR_ACTIVITY | 0.48890835 | 1.764764289 | 4.91E-05 | 0.000407837 |
| GOBP_RNA_CATABOLIC_PROCESS | 0.435930547 | 1.662917408 | 4.91E-05 | 0.000407837 |
| GOBP_POSITIVE_REGULATION_OF_DNA_BINDING_TRANSCRIPTION_FACTOR_ACTIVITY | 0.434219327 | 1.650474014 | 4.93E-05 | 0.000408787 |
| GOBP_DETECTION_OF_STIMULUS_INVOLVED_IN_SENSORY_PERCEPTION | -0.40969778 | -1.695289587 | 4.96E-05 | 0.000410176 |
| GOBP_MITOTIC_NUCLEAR_DIVISION | 0.434402376 | 1.665011186 | 4.97E-05 | 0.000410176 |
| GOBP_T_HELPER_17_CELL_LINEAGE_COMMITMENT | 0.851546855 | 1.985440148 | 5.04E-05 | 0.000415685 |
| GOBP_NEGATIVE_REGULATION_OF_ALPHA_BETA_T_CELL_ACTIVATION | 0.681340956 | 1.934023744 | 5.05E-05 | 0.000415685 |
| GOMF_PHOSPHOPROTEIN_BINDING | 0.558920063 | 1.888229161 | 5.06E-05 | 0.000415685 |
| GOBP_POSITIVE_REGULATION_OF_INTERFERON_BETA_PRODUCTION | 0.695203331 | 1.944797062 | 5.08E-05 | 0.000416409 |
| GOBP_CD8_POSITIVE_ALPHA_BETA_T_CELL_DIFFERENTIATION | 0.862618024 | 1.972310764 | 5.21E-05 | 0.000426185 |
| GOBP_SKIN_DEVELOPMENT | -0.367622025 | -1.569789154 | 5.25E-05 | 0.00042887 |
| GOBP_SEQUESTERING_OF_CALCIUM_ION | 0.512897338 | 1.793291466 | 5.28E-05 | 0.00043054 |
| GOBP_SECRETION_BY_TISSUE | -0.633559841 | -2.054770351 | 5.33E-05 | 0.000433799 |
| GOBP_PYROPTOSIS | 0.754573873 | 1.992837491 | 5.33E-05 | 0.000433799 |
| GOBP_SALIVA_SECRETION | -0.838437622 | -2.033474844 | 5.44E-05 | 0.00044135 |
| GOBP_REGULATION_OF_GTPASE_ACTIVITY | 0.415354434 | 1.61795855 | 5.46E-05 | 0.00044249 |
| GOBP_COLLAGEN_FIBRIL_ORGANIZATION | 0.614251137 | 1.934911712 | 5.50E-05 | 0.000443382 |
| GOBP_POSITIVE_REGULATION_OF_REACTIVE_OXYGEN_SPECIES_METABOLIC_PROCESS | 0.61365283 | 1.933027023 | 5.50E-05 | 0.000443382 |
| GOBP_NUCLEOTIDE_BINDING_OLIGOMERIZATION_DOMAIN_CONTAINING_2_SIGNALING_PATHWAY | 0.846726924 | 1.896489067 | 5.50E-05 | 0.000443382 |
| GOBP_REGULATION_OF_CYSTEINE_TYPE_ENDOPEPTIDASE_ACTIVITY | 0.459177812 | 1.717065139 | 5.56E-05 | 0.000447505 |
| GOBP_RESPONSE_TO_PEPTIDE | 0.381455227 | 1.52087376 | 5.69E-05 | 0.000457375 |

**Table 6b. KEGG of GSEA analysis.**

| ID | enrichmentScore | NES | pvalue | qvalue |
| --- | --- | --- | --- | --- |
| KEGG_AUTOIMMUNE_THYROID_DISEASE | 0.898128019 | 2.665075646 | 1.00E-10 | 6.69E-10 |
| KEGG_ANTIGEN_PROCESSING_AND_PRESENTATION | 0.815971394 | 2.632046932 | 1.00E-10 | 6.69E-10 |
| KEGG_ALLOGRAFT_REJECTION | 0.917166272 | 2.575008723 | 1.00E-10 | 6.69E-10 |
| KEGG_SYSTEMIC_LUPUS_ERYTHEMATOSUS | 0.851605364 | 2.572172944 | 1.00E-10 | 6.69E-10 |
| KEGG_LEISHMANIA_INFECTION | 0.804218144 | 2.563840357 | 1.00E-10 | 6.69E-10 |
| KEGG_PRIMARY_IMMUNODEFICIENCY | 0.899139993 | 2.55605361 | 1.00E-10 | 6.69E-10 |
| KEGG_GRAFT_VERSUS_HOST_DISEASE | 0.894403245 | 2.542588099 | 1.00E-10 | 6.69E-10 |
| KEGG_TYPE_I_DIABETES_MELLITUS | 0.88144619 | 2.538454086 | 1.00E-10 | 6.69E-10 |
| KEGG_NATURAL_KILLER_CELL_MEDIATED_CYTOTOXICITY | 0.705109253 | 2.458295147 | 1.00E-10 | 6.69E-10 |
| KEGG_ASTHMA | 0.898130113 | 2.442258975 | 1.00E-10 | 6.69E-10 |
| KEGG_HEMATOPOIETIC_CELL_LINEAGE | 0.737725137 | 2.422141327 | 1.00E-10 | 6.69E-10 |
| KEGG_CHEMOKINE_SIGNALING_PATHWAY | 0.657772995 | 2.407073617 | 1.00E-10 | 6.69E-10 |
| KEGG_TOLL_LIKE_RECEPTOR_SIGNALING_PATHWAY | 0.709736166 | 2.386729899 | 1.00E-10 | 6.69E-10 |
| KEGG_CYTOKINE_CYTOKINE_RECEPTOR_INTERACTION | 0.617215736 | 2.334815562 | 1.00E-10 | 6.69E-10 |
| KEGG_T_CELL_RECEPTOR_SIGNALING_PATHWAY | 0.678962263 | 2.319494761 | 1.00E-10 | 6.69E-10 |
| KEGG_CELL_ADHESION_MOLECULES_CAMS | 0.658273427 | 2.286882693 | 1.00E-10 | 6.69E-10 |
| KEGG_JAK_STAT_SIGNALING_PATHWAY | 0.610325348 | 2.183291473 | 1.00E-10 | 6.69E-10 |
| KEGG_VIRAL_MYOCARDITIS | 0.733303555 | 2.314185462 | 1.68E-10 | 1.06E-09 |
| KEGG_INTESTINAL_IMMUNE_NETWORK_FOR_IGA_PRODUCTION | 0.776046039 | 2.296226715 | 3.61E-09 | 2.16E-08 |
| KEGG_CYTOSOLIC_DNA_SENSING_PATHWAY | 0.750314652 | 2.264218169 | 7.95E-09 | 4.52E-08 |
| KEGG_B_CELL_RECEPTOR_SIGNALING_PATHWAY | 0.682109053 | 2.204728614 | 9.67E-09 | 5.23E-08 |
| KEGG_NOD_LIKE_RECEPTOR_SIGNALING_PATHWAY | 0.701467842 | 2.16976162 | 8.60E-08 | 4.44E-07 |
| KEGG_FC_GAMMA_R_MEDIATED_PHAGOCYTOSIS | 0.625694055 | 2.074413474 | 1.27E-07 | 6.29E-07 |
| KEGG_SPLICEOSOME | 0.589773633 | 2.027856493 | 3.13E-07 | 1.44E-06 |
| KEGG_LYSOSOME | 0.57504921 | 1.981027368 | 3.16E-07 | 1.44E-06 |
| KEGG_LEUKOCYTE_TRANSENDOTHELIAL_MIGRATION | 0.584455876 | 2.000930503 | 6.07E-07 | 2.65E-06 |
| KEGG_APOPTOSIS | 0.596754784 | 1.975648013 | 2.03E-06 | 8.23E-06 |
| KEGG_CELL_CYCLE | 0.561283646 | 1.944180394 | 1.97E-06 | 8.23E-06 |
| KEGG_FC_EPSILON_RI_SIGNALING_PATHWAY | 0.630491694 | 2.024672405 | 2.74E-06 | 1.07E-05 |
| KEGG_DRUG_METABOLISM_CYTOCHROME_P450 | -0.605862994 | -2.075299488 | 3.71E-05 | 0.000140691 |
| KEGG_RNA_DEGRADATION | 0.642197669 | 1.94544553 | 5.27E-05 | 0.000193157 |
| KEGG_COMPLEMENT_AND_COAGULATION_CASCADES | 0.58618997 | 1.868768459 | 0.000122738 | 0.000436045 |
| KEGG_ECM_RECEPTOR_INTERACTION | 0.547225217 | 1.788437117 | 0.000138012 | 0.000475449 |
| KEGG_PROTEASOME | 0.668558144 | 1.939444454 | 0.000172143 | 0.000575588 |
| KEGG_DNA_REPLICATION | 0.675234386 | 1.930295954 | 0.000183201 | 0.000595059 |
| KEGG_PATHWAYS_IN_CANCER | 0.401611991 | 1.540098263 | 0.00023367 | 0.000737905 |
| KEGG_RIG_I_LIKE_RECEPTOR_SIGNALING_PATHWAY | 0.57018018 | 1.809200307 | 0.00040188 | 0.001234796 |
| KEGG_GLYCINE_SERINE_AND_THREONINE_METABOLISM | -0.662254438 | -1.933799694 | 0.000445803 | 0.001317599 |
| KEGG_P53_SIGNALING_PATHWAY | 0.571817274 | 1.80456131 | 0.00045201 | 0.001317599 |
| KEGG_PRION_DISEASES | 0.649695806 | 1.84132047 | 0.000531817 | 0.00151148 |
| KEGG_UBIQUITIN_MEDIATED_PROTEOLYSIS | 0.475334605 | 1.651341883 | 0.000712783 | 0.001976396 |
| KEGG_METABOLISM_OF_XENOBIOTICS_BY_CYTOCHROME_P450 | -0.53256366 | -1.821441026 | 0.000809094 | 0.002190028 |
| KEGG_FOCAL_ADHESION | 0.426367709 | 1.572591827 | 0.000845065 | 0.002234199 |
| KEGG_TYROSINE_METABOLISM | -0.589837475 | -1.840939698 | 0.001081652 | 0.0027947 |
| KEGG_PATHOGENIC_ESCHERICHIA_COLI_INFECTION | 0.581560376 | 1.761753566 | 0.001111064 | 0.002806899 |
| KEGG_PROXIMAL_TUBULE_BICARBONATE_RECLAMATION | -0.6849369 | -1.86051189 | 0.001461745 | 0.00361255 |
| KEGG_NEUROTROPHIN_SIGNALING_PATHWAY | 0.464520792 | 1.609012171 | 0.001524699 | 0.003687962 |
| KEGG_VALINE_LEUCINE_AND_ISOLEUCINE_DEGRADATION | -0.554844129 | -1.774564955 | 0.001861466 | 0.004408736 |
| KEGG_SMALL_CELL_LUNG_CANCER | 0.493319226 | 1.625258168 | 0.002235579 | 0.005186736 |
| KEGG_ENDOCYTOSIS | 0.431772303 | 1.568682698 | 0.00265603 | 0.006038973 |
| KEGG_BUTANOATE_METABOLISM | -0.590618477 | -1.762314352 | 0.003670543 | 0.008182015 |
| KEGG_MAPK_SIGNALING_PATHWAY | 0.376426357 | 1.423873059 | 0.005034596 | 0.011006809 |
| KEGG_REGULATION_OF_ACTIN_CYTOSKELETON | 0.396367019 | 1.472899858 | 0.006089369 | 0.013061606 |
| KEGG_COLORECTAL_CANCER | 0.508550935 | 1.588644992 | 0.007135396 | 0.015021885 |
| KEGG_EPITHELIAL_CELL_SIGNALING_IN_HELICOBACTER_PYLORI_INFECTION | 0.500411931 | 1.595308825 | 0.007979299 | 0.016198577 |
| KEGG_PANCREATIC_CANCER | 0.49664007 | 1.588881032 | 0.007844182 | 0.016198577 |
| KEGG_CHRONIC_MYELOID_LEUKEMIA | 0.486816915 | 1.56310968 | 0.008351653 | 0.016657037 |
| KEGG_HISTIDINE_METABOLISM | -0.593264601 | -1.718354227 | 0.010676583 | 0.020926877 |
| KEGG_O_GLYCAN_BIOSYNTHESIS | -0.581314054 | -1.683740207 | 0.013270825 | 0.02514472 |
| KEGG_ARGININE_AND_PROLINE_METABOLISM | -0.491802175 | -1.61355979 | 0.013105008 | 0.02514472 |
| KEGG_RENAL_CELL_CARCINOMA | 0.48517495 | 1.539475939 | 0.013631466 | 0.02540463 |
| KEGG_MISMATCH_REPAIR | 0.622541391 | 1.618556352 | 0.016064455 | 0.029456047 |
| KEGG_PROPANOATE_METABOLISM | -0.546044494 | -1.594463721 | 0.019938209 | 0.035416555 |
| KEGG_RETINOL_METABOLISM | -0.495396974 | -1.592873086 | 0.019856529 | 0.035416555 |
| KEGG_CARDIAC_MUSCLE_CONTRACTION | -0.411424278 | -1.455160269 | 0.026651763 | 0.046613609 |
| KEGG_ASCORBATE_AND_ALDARATE_METABOLISM | -0.63445811 | -1.561855893 | 0.030684027 | 0.052852869 |
| KEGG_PHENYLALANINE_METABOLISM | -0.602446305 | -1.533100476 | 0.033869903 | 0.057469749 |
| KEGG_FATTY_ACID_METABOLISM | -0.479663963 | -1.497077532 | 0.035070543 | 0.058631867 |
| KEGG_ARRHYTHMOGENIC_RIGHT_VENTRICULAR_CARDIOMYOPATHY_ARVC | -0.38752719 | -1.377196765 | 0.038287072 | 0.063081674 |
| KEGG_HOMOLOGOUS_RECOMBINATION | 0.549720058 | 1.494837693 | 0.041204437 | 0.066918485 |
| KEGG_VASCULAR_SMOOTH_MUSCLE_CONTRACTION | -0.346782069 | -1.311663425 | 0.042092077 | 0.067397247 |
| KEGG_NON_HOMOLOGOUS_END_JOINING | 0.688022765 | 1.538116705 | 0.043665745 | 0.068945914 |
| KEGG_ETHER_LIPID_METABOLISM | 0.553792345 | 1.491919839 | 0.046474359 | 0.071508208 |
| KEGG_VEGF_SIGNALING_PATHWAY | 0.435197444 | 1.396998048 | 0.046546547 | 0.071508208 |
| KEGG_AMYOTROPHIC_LATERAL_SCLEROSIS_ALS | 0.473831524 | 1.440549125 | 0.047619048 | 0.072180451 |
| KEGG_TIGHT_JUNCTION | -0.343758653 | -1.335002894 | 0.048460774 | 0.072489801 |

# Appendix 7

**Immune Correlation Analysis**

**Table 7. Immune Correlation Analysis.**

| Gene | Cell | cor | pvalue |
| --- | --- | --- | --- |
| IRF8 | B cells naive | 0.535864811 | 8.57E-19 |
| IRF8 | B cells memory | -0.107927353 | 0.099569528 |
| IRF8 | Plasma cells | -0.695547318 | 3.65E-35 |
| IRF8 | T cells CD8 | -0.354975722 | 2.35E-08 |
| IRF8 | T cells CD4 naive | 0.273411435 | 2.22E-05 |
| IRF8 | T cells CD4 memory resting | 0.22407329 | 0.000553728 |
| IRF8 | T cells CD4 memory activated | 0.397374588 | 2.83E-10 |
| IRF8 | T cells follicular helper | 0.056067271 | 0.393247246 |
| IRF8 | T cells regulatory (Tregs) | -0.295232594 | 4.33E-06 |
| IRF8 | T cells gamma delta | 0.264725495 | 4.11E-05 |
| IRF8 | NK cells resting | 0.076486713 | 0.243831115 |
| IRF8 | NK cells activated | -0.369386411 | 5.62E-09 |
| IRF8 | Monocytes | -0.383821121 | 1.25E-09 |
| IRF8 | Macrophages M0 | 0.543723852 | 2.11E-19 |
| IRF8 | Macrophages M1 | 0.396723678 | 3.04E-10 |
| IRF8 | Macrophages M2 | -0.005962925 | 0.927709077 |
| IRF8 | Dendritic cells resting | 0.070123338 | 0.285406802 |
| IRF8 | Dendritic cells activated | -0.124386719 | 0.057440117 |
| IRF8 | Mast cells resting | -0.144688226 | 0.026890044 |
| IRF8 | Mast cells activated | 0.05330533 | 0.41700662 |
| IRF8 | Eosinophils | 0.049464591 | 0.451406707 |
| IRF8 | Neutrophils | 0.056351718 | 0.390847476 |

# Appendix 8

**MiRNA and LncRNA**

**Table 8a. Gene-miRNA.**

| Gene | miRNA | miRanda | miRDB | TargetScan | Sum |
| --- | --- | --- | --- | --- | --- |
| IRF8 | hsa-miR-155-5p | 1 | 1 | 1 | 3 |
| IRF8 | hsa-miR-1276 | 1 | 1 | 1 | 3 |
| IRF8 | hsa-miR-1286 | 1 | 1 | 1 | 3 |
| IRF8 | hsa-miR-545-3p | 1 | 1 | 1 | 3 |
| IRF8 | hsa-miR-548b-5p | 1 | 1 | 1 | 3 |
| IRF8 | hsa-miR-618 | 1 | 1 | 1 | 3 |
| IRF8 | hsa-miR-452-5p | 1 | 1 | 1 | 3 |
| IRF8 | hsa-miR-4291 | 1 | 1 | 1 | 3 |
| IRF8 | hsa-miR-548d-5p | 1 | 1 | 1 | 3 |
| IRF8 | hsa-miR-194-5p | 1 | 1 | 1 | 3 |
| IRF8 | hsa-miR-3168 | 1 | 1 | 1 | 3 |
| IRF8 | hsa-miR-938 | 1 | 1 | 1 | 3 |
| IRF8 | hsa-miR-205-3p | 1 | 1 | 1 | 3 |
| IRF8 | hsa-miR-559 | 1 | 1 | 1 | 3 |
| IRF8 | hsa-miR-548h-5p | 1 | 1 | 1 | 3 |
| IRF8 | hsa-miR-548a-5p | 1 | 1 | 1 | 3 |
| IRF8 | hsa-miR-4288 | 1 | 1 | 1 | 3 |
| IRF8 | hsa-miR-4270 | 1 | 1 | 1 | 3 |
| IRF8 | hsa-miR-186-5p | 1 | 1 | 1 | 3 |
| IRF8 | hsa-miR-30c-2-3p | 1 | 1 | 1 | 3 |
| IRF8 | hsa-miR-548w | 1 | 1 | 1 | 3 |
| IRF8 | hsa-miR-4282 | 1 | 1 | 1 | 3 |
| IRF8 | hsa-miR-548c-5p | 1 | 1 | 1 | 3 |
| IRF8 | hsa-miR-302a-5p | 1 | 1 | 1 | 3 |
| IRF8 | hsa-miR-548i | 1 | 1 | 1 | 3 |
| IRF8 | hsa-miR-578 | 1 | 1 | 1 | 3 |
| IRF8 | hsa-miR-548n | 1 | 1 | 1 | 3 |
| IRF8 | hsa-miR-3116 | 1 | 1 | 1 | 3 |
| IRF8 | hsa-miR-3126-3p | 1 | 1 | 1 | 3 |
| IRF8 | hsa-miR-346 | 1 | 1 | 1 | 3 |

**Table 8b. Gene-miRNA.**

| miRNA | lncRNA |
| --- | --- |
| hsa-miR-194-5p | CTA-414D7.1 |
| hsa-miR-545-3p | LINC01070 |
| hsa-miR-186-5p | RP11-99L13.2 |
| hsa-miR-186-5p | MIR325HG |
| hsa-miR-545-3p | LINC01165 |
| hsa-miR-186-5p | LINC00613 |
| hsa-miR-186-5p | DYX1C1-CCPG1 |
| hsa-miR-302a-5p | RP11-343D2.11 |
| hsa-miR-186-5p | RP11-154D6.1 |
| hsa-miR-186-5p | RP11-22A3.2 |
| hsa-miR-186-5p | SFTPD-AS1 |
| hsa-miR-186-5p | RP1-288H2.2 |
| hsa-miR-186-5p | AC124997.1 |
| hsa-miR-186-5p | CTD-2410N18.4 |
| hsa-miR-186-5p | AJ003147.8 |
| hsa-miR-186-5p | CTD-3046C4.1 |
| hsa-miR-186-5p | RP11-227H15.4 |
| hsa-miR-938 | RP11-989E6.10 |
| hsa-miR-186-5p | LINC00662 |
| hsa-miR-186-5p | CTB-181F24.1 |
| hsa-miR-618 | RP11-627J17.1 |
| hsa-miR-186-5p | SNHG14 |
